# The Genome of *Tolypocladium inflatum*: Evolution, Organization, and Expression of the Cyclosporin Biosynthetic Gene Cluster

**DOI:** 10.1371/journal.pgen.1003496

**Published:** 2013-06-20

**Authors:** Kathryn E. Bushley, Rajani Raja, Pankaj Jaiswal, Jason S. Cumbie, Mariko Nonogaki, Alexander E. Boyd, C. Alisha Owensby, Brian J. Knaus, Justin Elser, Daniel Miller, Yanming Di, Kerry L. McPhail, Joseph W. Spatafora

**Affiliations:** 1 Department of Botany and Plant Pathology, Oregon State University, Corvallis, Oregon, United States of America; 2 College of Pharmacy, Oregon State University, Corvallis, Oregon, United States of America; 3 Center for Genome Research & Biocomputing, Oregon State University, Corvallis, Oregon, United States of America; 4 Department of Statistics, Oregon State University, Corvallis, Oregon, United States of America; Duke University Medical Center, United States of America

## Abstract

The ascomycete fungus *Tolypocladium inflatum*, a pathogen of beetle larvae, is best known as the producer of the immunosuppressant drug cyclosporin. The draft genome of *T. inflatum* strain NRRL 8044 (ATCC 34921), the isolate from which cyclosporin was first isolated, is presented along with comparative analyses of the biosynthesis of cyclosporin and other secondary metabolites in *T. inflatum* and related taxa. Phylogenomic analyses reveal previously undetected and complex patterns of homology between the nonribosomal peptide synthetase (NRPS) that encodes for cyclosporin synthetase (*simA*) and those of other secondary metabolites with activities against insects (e.g., beauvericin, destruxins, etc.), and demonstrate the roles of module duplication and gene fusion in diversification of NRPSs. The secondary metabolite gene cluster responsible for cyclosporin biosynthesis is described. In addition to genes necessary for cyclosporin biosynthesis, it harbors a gene for a cyclophilin, which is a member of a family of immunophilins known to bind cyclosporin. Comparative analyses support a lineage specific origin of the cyclosporin gene cluster rather than horizontal gene transfer from bacteria or other fungi. RNA-Seq transcriptome analyses in a cyclosporin-inducing medium delineate the boundaries of the cyclosporin cluster and reveal high levels of expression of the gene cluster cyclophilin. In medium containing insect hemolymph, weaker but significant upregulation of several genes within the cyclosporin cluster, including the highly expressed cyclophilin gene, was observed. *T. inflatum* also represents the first reference draft genome of Ophiocordycipitaceae, a third family of insect pathogenic fungi within the fungal order Hypocreales, and supports parallel and qualitatively distinct radiations of insect pathogens. The *T. inflatum* genome provides additional insight into the evolution and biosynthesis of cyclosporin and lays a foundation for further investigations of the role of secondary metabolite gene clusters and their metabolites in fungal biology.

## Author Summary

*Tolypocladium inflatum*, the fungus from which the immunosuppressant drug cyclosporin was isolated, is a prolific producer of secondary metabolites with potential applications in medicine and agriculture. We have sequenced the first draft reference genome of *T. inflatum*, which also represents the first genome of a novel family of insect pathogenic fungi, Ophiocordycipitaceae. We present comparative genomic and evolutionary analyses of the cyclosporin nonribosomal peptide synthetase (*simA*), which highlight the lineage specific nature of cyclosporin's origin and the homology of cyclosporin adenylation (A) domains with other fungal NRPSs whose products show anti-insect activity. RNA-Seq data profiles the expression patterns of the cyclosporin gene cluster in an inducing medium and in response to media simulating distinct stages of insect pathogenesis. Sequencing of the *T. inflatum* genome has uncovered the metabolite gene cluster responsible for cyclosporin biosynthesis and characterized complex patterns of its evolution.

## Introduction

Fungi are prolific producers of secondary metabolites and an important source of novel and commercially important pharmaceuticals, mycoinsecticides, and antibiotics. Cyclosporin A (CsA; CAS ID: 59865-13-3), the well-known immunosuppressant drug which revolutionized organ transplantation from an experimental to a relatively routine lifesaving procedure [1], was first discovered in the insect pathogenic and ubiquitous soil fungus, *Tolypocladium inflatum*. CsA targets and binds with high affinity human cyclophilin A (hCypA, peptidylprolyl isomerase A, EC: 5.2.1.8), a conserved immunophilin found across eukaryotes [2], [3]. The CsA-hCypA complex suppresses the vertebrate immune system by binding to and inhibiting calcineurin, a conserved calcium-calmodulin activated serine/threonine-specific protein phosphatase (EC: 3.1.3.16) [4], [5]. Inhibition of calcineurin blocks activity of NF-AT (nuclear factor of activated T-cells), a regulator of transcription of Interleukin 2 in T-lymphocytes [6]. CsA also impairs the immune response in insects and shows both antifungal [7] and antiviral activity [8]. CsA and related cyclosporins (B-Z and isoforms) form a family of cyclic undecapeptides produced by nonribosomal peptide synthetases (NRPSs), a class of large multimodular enzymes that produce peptides via a nonribosomal mechanism [9]. While the 45.8 kb locus encoding the NRPS synthetase (*simA*) responsible for biosynthesis of cyclosporin was cloned in 1994 [10], the complete biosynthetic cluster remains uncharacterized.

*T. inflatum* belongs to the fungal order Hypocreales, containing fungi known to produce a high diversity of bioactive secondary metabolites [11]. In addition to cyclosporins, *T. inflatum* synthesizes a number of other products via both NRPSs and polyketide synthetases (PKSs), another class of multimodular enzyme involved in secondary metabolite production in bacteria and fungi [12]. Other hypocrealean fungi are known to produce NRPS or PKS products with activity against insects, such as destruxins (*Metarhizium robertsii*) [13], efrapeptins (*Tolypocladium* spp.) [14], and ergot alkaloids (clavicipitalean endophytes of grasses including *Claviceps* and *Epichloë* spp.) [15]. Many of these compounds also have pharmaceutical applications and/or roles in antibiosis, pathogenesis, and competitive interactions between organisms [13], [14]. The genome sequence of *T. inflatum* thus provides an opportunity to characterize the secondary metabolite arsenal of an insect-pathogenic fungus with potential for both elucidation of the biosynthetic cluster of the immunosuppressant drug cyclosporin and discovery of novel gene clusters and metabolites with applications in medicine and agriculture.

**Figure 1:**
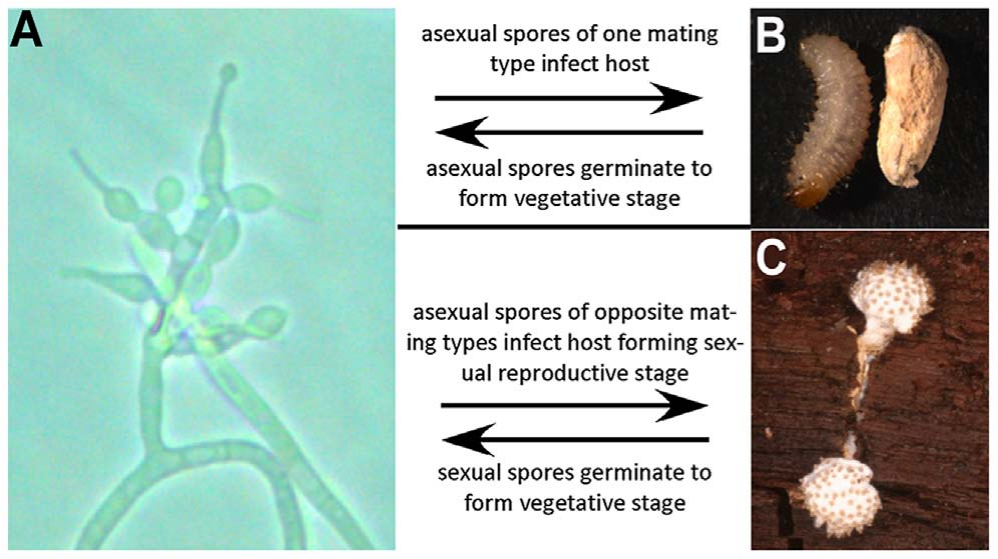
Lifecycle of *T. inflatum*. The lifecycle of *T. inflatum* comprises both an asexual stage (primarily saprotrophic growth in soil) and a sexual stage (only occurs on an insect host). A) Asexual reproductive stage growing on cornmeal agar produces asexual spores on phialides with diagnostic inflated bases. B) Uninfected beetle larva (left) and beetle larva infected with asexual spores (right). C) Sexual reproductive stage growing off of an infected beetle host buried in wood. The sexual stage only occurs when two spores of opposite mating type infect the same insect host.

Hypocrealean fungi display considerable flexibility of lifestyles. They include plant-pathogens, plant-saprobes, plant-endophytes, mycoparasites, and pathogens of insects, spiders, rotifers, and nematodes. Transitions between different lifestyles have occurred multiple times in the evolutionary history of the order [16], [17]. *T. inflatum* is a pathogen of beetle larvae [18], but is also able to live saprotrophically in soil during the asexual phase of its lifecycle (Figure 1). It is one of the few insect pathogenic (entomopathogenic) fungi sequenced to date, although Hypocreales contains three families (Clavicipitaceae, Cordycipitaceae, and Ophiocordycipitaceae) that are particularly rich in entomopathogenic species. The genomes of *M. robertsii* and *M. acridum* (Clavicipitaceae) have provided insights into expansions of gene families, especially those for secreted proteins, with roles in insect pathogenesis [19]. Other studies have shown changes in the profiles of carbohydrate active enzymes (CAZymes), cytochrome P450s, and proteases in insect pathogens when compared to closely related plant pathogens [20]. The genome of *Cordyceps militaris* (Cordycipitaceae), a common pathogen of moth pupae used in traditional Chinese Medicine, revealed aspects of the mating systems of entomopathogenic fungi [21]. These taxa belong to three separate families of Hypocreales that represent parallel diversifications of entomopathogenic fungi [16].

Here we present the results from whole genome sequencing and RNA-Seq analyses of *T. inflatum* (Ophiocordycipitaceae), which represents the first draft genome from the third entomopathogenic family within Hypocreales. Through phylogenomic and comparative genomic analyses we demonstrate that the NRPS responsible for cyclosporin biosynthesis (*simA*) exhibits complex patterns of homology with other NRPSs and that the cyclosporin gene cluster is unique to *Tolypocladium* with no evidence for horizontal transfer of a complete cluster from other fungi or bacteria. RNA-Seq analyses in a cyclosporin-inducing medium clearly delineate a secondary metabolite cluster responsible for cyclosporin biosynthesis. RNA-Seq analyses in media simulating insect pathogenesis indicate that several genes within the cluster, including the homolog of the cyclosporin binding protein cyclophilin, are also upregulated in response to insect hemolymph, supporting a role for both cyclosporin and the cyclophilin gene in pathogenesis of insects.

## Results/Discussion

### General Genome Features

A karyotype study of the sequenced strain *T. inflatum* NRRL 8044 (ATCC 34921) indicated that *T. inflatum* has 6 chromosomes ranging in size from 3.8 to 6.6 Mb and a mini supernumerary chromosome of 1 Mb with a total genome size of approximately 30.45 Mb [22]. The total size of the assembly (30.348 Mb) closely matched this estimate and contained 194 contigs in 101 scaffolds with an N50 of 1.5 Mb and an Nmax of 3.56 Mb. The MAKER 2.0 annotation pipeline [23] predicted 9,998 protein coding genes (loci tagged as TINF) with greater than 90% having support from either protein (*Fusarium graminearum*, *Nectria haematococca*, *Trichoderma reesei*, *Tr. virens*, and *M. robertsii*) or EST data (*C. militaris*, *Beauveria bassiana*, *M. robertsii*, *Tr. reesei*, and assembled *T. inflatum* RNA-Seq reads). Analysis using the Core Eukaryotic Genes Mapping Approach (CEGMA) pipeline [24] estimated that the annotations represent >98% of coding regions based on completeness of a conserved set of eukaryotic proteins. The average gene length (1.67 kb), exon length (570 bp) and intron length (77.5 bp) were similar to estimates from other Ascomycota (Table 1) [25]. However, *T. inflatum* has a higher average GC content (58%) and a more compact genome with higher gene density (329 genes/Mb) than closely related filamentous ascomycetes (Table 1). Only the *MAT1-2* mating type locus was detected in the sequenced strain, indicating that *T. inflatum* is likely heterothallic (Figure S1).

**Table 1:** Comparison of genome features with closely related ascomycetes.

Feature	*T. inflatum*	*M. robertsii*	*M. acridum*	*C. militaris*	*F. graminearum*	*N. haematococca*	*N. crassa*
Genome Size (Mb)	30.35	39.00	38.00	32.2	36.09	53.43	38.04
% GC Content	58.0	51.5	50.0	51.4	48.3	51.7	48.2
Predicted Proteins	9,998	10,582	9,849	9,684	13,321	15,707	10,082
Avg. Gene Density (genes/Mb)	329	271	259	301	369	294	265
Avg. Gene Length (bp)	1,670	1,700	1,672	1,742	1,583	1,670	1,673
Avg. Exon Length (bp)	570.0	567.2	548.5	506.5	507.5	468.5	356.0
Avg. Intron Length	77.5	106.3	112.2	97.9	68.0	84.0	134.0
Avg. #Introns/gene	2.2	1.8	1.7	2.0	2.2	2.1	1.7
Repeat Content %	1.24	0.98	1.52	3.04	0.10	5.10	3.15
tRNAs	112	363	440	102	314	285	415

### Repeat Elements

The estimated proportion of repeat sequence (1.24%), which agrees well with previous experimental estimates (1%) [26], is relatively low compared to other filamentous ascomycetes (Table 1). In total, *T. inflatum* contained a slightly larger number of retrotransposons compared to DNA transposons (Table S1). Retrotransposons were dominated by two classes, LINE elements and the Gypsy family of LTRs, while DNA transposons were mostly comprised of the hAT family (Table S1). Several novel repeat elements, including the CPA (cyclosporin production associated) element [26] and the first characterized fungal hAT transposon (*Restless*) [27], were previously identified in the sequenced strain (NRRL 8044). The CPA element, which shows greatest similarity to a RecQ DNA helicase in *M. robertsii* [28], was named based on the observation that multiple copies were found only in cyclosporin producing strains of *T. inflatum* while only a single copy was present in other strains of *T. inflatum* and related *Tolypocladium* and *Beauveria* species. We identified 12 copies of the CPA element in *T. inflatum* NRRL 8044 that were found dispersed across eleven scaffolds, but none of them associated with the scaffold containing the cyclosporin biosynthetic cluster. A single partial copy was also found in *C. militaris* and *Tr. virens* and multiple copies were present in *Tr. reesei*, *Metarhizium* spp., and especially *N. haematococca*, in which the transposon has undergone expansion to 27 copies (Table S1). We conclude that presence and expansion of CPA elements is not unique to *T. inflatum* and that it is not associated with the evolution or production of cyclosporin. Similarly, copies of *Restless* were also found in other hypocrealean taxa and were particularly expanded in *F. oxysporum*, which harbored over double (55) the number of elements in *T. inflatum* (26) (Table S1). *Restless* generates partial deletion copies of the transposon and several deletion variants (*ΔRst* 1-6) have been found in *T. inflatum*, *Neurospora crassa*, and *Penicillium chrysogenum* [29]. With the exception of *Tr. atroviride*, all hypocrealean taxa contained either an intact or a deletion variant of *Restless*, indicating the transposon was present in the ancestor of Hypocreales. However, of the genomes analyzed, deletion variants were most abundant and diverse in *T. inflatum* (Table S1), suggesting it has been particularly active in *T. inflatum*.

### Phylogenomic Relationships and Orthologous Gene Clusters

**Figure 2:**
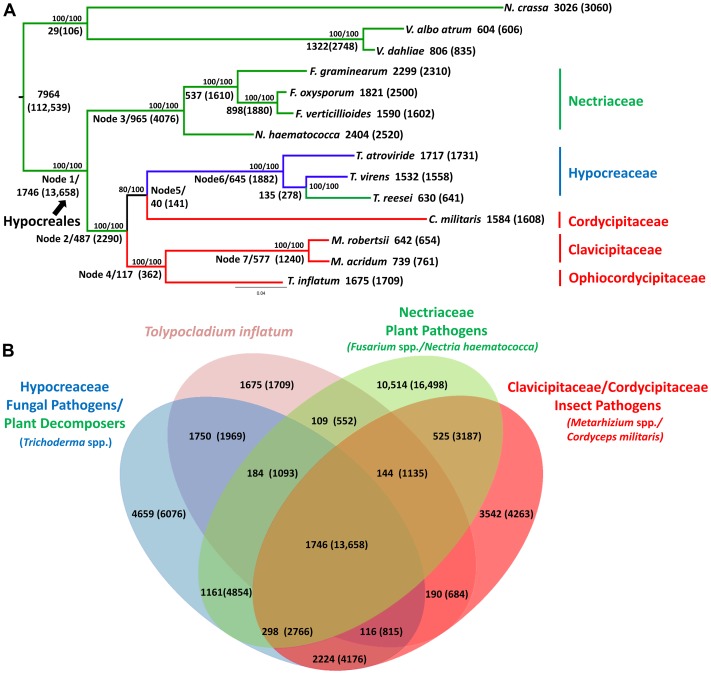
Phylogenetic relationships and orthologous gene clusters. A) Maximum likelihood phylogeny created from a concatenated alignment of 2769 groups of single copy orthologs identified by the Hal pipeline. Phylogeny constructed using RAxML with best models for each cluster partition identified using ProtTest. Bootstrap values for analyses with the original alignment (top)/the alignment with fast-evolving sites removed (bottom) are shown above nodes. Larger numbers beneath or adjacent to nodes and terminal taxa indicate the number of clusters and genes (in parentheses) within those clusters that map to each node in the phylogeny or are unique to a species. Color coding corresponds to fungal host: green = plant associated, blue = fungal associated, red = animal or insect associated. Hypocreales is delineated at node 1. A major shift from early diverging taxa that have primarily plant-associated hosts to either animal/insect or fungal hosts occurs at node 2. B) The number of both clusters and number of genes (in parentheses) in those clusters that are shared by *T. inflatum* with each of the major families and associated ecologies within Hypocreales: green = Nectriaceae, primarily plant associated including *F. graminearum*, *F. oxysporum*, *F. verticillioides*, and *N. haematococca*; blue = Hypocreaceae, primarily fungal associated (*Tr. atroviride* and *Tr. virens*) or plant saprobic (*Tr. reesei*); red = Clavicipitaceae/Cordycipitaceae, primarily animal or insect associated including *C. militaris*, *M. robertsii*, *M. acridum*; and pink = *T. inflatum*.

*T. inflatum* is a member of the class Sordariomycetes and is related to the widely studied filamentous ascomycete *N. crassa*, which together with the wilt pathogens *Verticillium dahliae* and *Verticillium albo-atrum*, served as an outgroup to the order Hypocreales in our phylogenomic analyses (Figure 2A, node 1). Orthologous clustering of proteins using MCL [30] identified a total of 36,532 orthologous clusters of proteins across the 14 taxa analyzed (*N. crassa, V. dahliae, V. albo-atrum, N. haematococca, F. graminearum, F. oxysporum, F. verticillioides, Tr. virens, Tr. atroviride, Tr. reesei, C. militaris, M. robertsii, M. acridum, and T. inflatum*). Using the phylogenomic pipeline Hal [31], these 36,532 clusters were filtered to identify 2,769 clusters containing only single-copy orthologous proteins. A concatenated alignment was built from this subset of clusters and used to construct a maximum likelihood phylogeny.

The inferred phylogeny recovered a topology consistent with the four major families of Hypocreales (Figure 2A). The earliest diverging group, Nectriaceae, comprises primarily plant pathogenic species including the wheat head blight fungus *F. graminearum* and related species *F. oxysporum*, *F. verticillioides*, and *N. haematococca* (Figure 2A, node 3, green). Sequenced representatives of Hypocreaceae include members of the genus *Trichoderma* (*Tr. atroviride, Tr. reesei, Tr.virens*). Although the ability of *Trichoderma* spp. to grow on and digest plant-based compounds is well-documented, recent comparative genomic studies support an evolutionary history characterized by mycoparasitism [32](Figure 2A, node 6, blue). Hypocreaceae forms a sister group (node 5) to the primarily insect pathogenic Cordycipitaceae, which includes the moth pathogen and traditional Chinese medicinal fungus *C. militaris*. The remaining two families, Ophiocordycipitaceae, of which *T. inflatum* is the first sequenced representative, and Clavicipitaceae (Figure 2A, node 7, red), which includes the two insect pathogenic biocontrol species *M. robertsii* and *M. acridum*, comprise, with the exception of the clavicipitaceous endophytes, primarily insect pathogens. This topology is consistent with standard multigene (e.g., five to seven loci) phylogenetic analyses that have sampled an order of magnitude more species of Hypocreales and ancestral character state reconstructions from previously published multigene datasets [16], [17], which support a major transition within the order from plant hosts/substrates in early diverging lineages (Nectriaceae) to primarily insect (Clavicipitaceae, Cordycipitaceae, Ophiocordycipitaceae) or fungal (Hypocreaceae) hosts (Figure 2A, node 2). However, the placement of Cordycipitaceae has been controversial. The removal of fast evolving sites [33] in these genome-scale analyses provides stronger bootstrap support for the placement of *C. militaris* (Cordycipitaceae) as monophyletic with *Trichoderma* (Hypocreaceae) and not with the other insect pathogens of *Metarhizium* (Clavicipitaceae) and *Tolypocladium* (Ophiocordycipitaceae). These results provide additional support for polyphyletic origins and parallel diversifications of insect pathogenic fungi in three separate families in Hypocreales [34].

Out of the total 36,532 orthologous clusters, those shared by one or more descendants of each node were mapped to the phylogeny to produce a phylogenetic profile of orthologous clusters (Figure 2A). A total of 7,964 clusters containing 112,539 proteins from both within Hypocreales and from outgroup taxa (*N. crassa*, *V. dahliae* and *V. albo atrum*), while 1,746 clusters containing 13,658 proteins mapped uniquely to the node representing the origin of Hypocreales (Figure 2A, node 1). Within Hypocreales, plant associated species in the Nectriaceae had the largest number of unique clusters although these genomes also contained a larger number of protein coding genes per genome (Table 1). *T. inflatum* had 1,675 species-unique clusters containing 1,709 proteins (out of a total of 9998). *T. inflatum* shared a larger number of clusters with fungal pathogens in Hypocreaceae (1750) than with other insect pathogens in Clavicipitaceae and Cordycipitaceae (190) or with plant pathogens in Nectriaceae (109) (Figure 2B).

### Gene Content and Evolution

**Figure 3:**
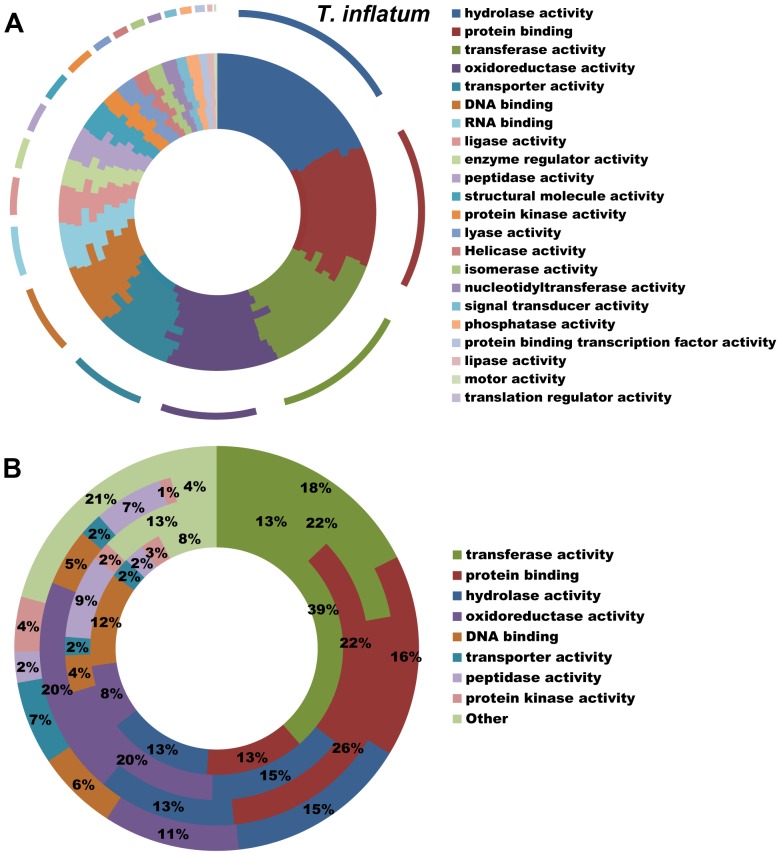
Functional classification of gene content. A) Profiles of GO Slim (*Aspergillus* GO Slim) molecular function categories for hypocrealean taxa. Taxa from inside of circle to outside of circle are *F. oxysporum*, *F. verticillioides*, *F. graminearum*, *N. haematococca*, *Tr. atroviride*, *Tr. reesei*, *Tr. virens*, *C. militaris*, *M. acridum*, *M. robertsii*, and *T. inflatum*. B) Profiles of GO Slim molecular function categories for genes in species-unique orthologous clusters (Figure 3A) showing percentage of genes in each category out of total annotated genes. Taxa from inside to outside are *C. militaris*, *M. robertsii*, *M. acridum*, and *T. inflatum*.

All hypocrealean taxa, including *T. inflatum*, shared a similar profile of Gene Ontology (GO) Slim categories (Figures 3A, S2). However, GO Slim profiles of genes found in orthologous clusters unique to each of the insect pathogens *C. militaris* (Cordycipitaceae), *M. robertsii* and *M. acridum* (Clavicipitaceae), and *T. inflatum* (Ophiocordycipitaceae) showed lineage specific differences (Figure 3B). *Metarhizium* spp. (Clavicipitaceae) had a larger proportion of species-unique genes associated with GO molecular functions of protein binding (22–26% vs 13–16%), oxidoreductase activity (20% vs 8–11%), and peptidase activity (7–9% vs 2–3%) relative to either *T. inflatum* or *C. militaris* (Figures 3B, S2B). These results are consistent with expansions of both proteases (peptidase activity) and P450s (oxidative activity) in *Metarhizium* spp., *Tr. virens*, and all plant pathogenic species in Nectriaceae, but not in the other insect pathogens *T. inflatum* or *C. militaris* (Table S2). In fact, *M. acridum* and particularly *M. robertsii*, which is known to live in association with the plant rhizosphere [35], showed overall profiles of CAZymes, proteases, and P450s more similar to plant pathogens than to the other insect pathogens (Table S2). *T. inflatum* contained a larger proportion of species-unique genes associated with the GO molecular function of transporter activity (7% vs 2%) than other insect pathogens, while *C. militaris* had a larger proportion of species-unique genes associated with transferase activity (39% vs 13–22%) (Figures 3B, S2B). For GO biological process categories, *T. inflatum* also contained a larger proportion of species-unique genes related to transport (11% vs 3–4%) but a smaller proportion of genes involved in DNA-dependent transcription (7% vs 18–23%) relative to other insect pathogens (Figure S2D). A large proportion of species-unique genes in all insect pathogens (31–74%) were associated with membranes, particularly endomembrane systems (Figure S2F), consistent with the importance of secreted proteins in these fungi [19]. These differences in gene content and ontology between the three insect pathogenic lineages corroborate phylogenetic evidence for parallel and qualitatively distinct evolutionary radiations of insect pathogens in Hypocreales that reflect adaptations to distinct ecologies.

### Overview of Secondary Metabolite Gene Clusters

While *T. inflatum* is best known as the original source of CsA [36], it is also known to produce other bioactive secondary metabolites including insecticidal compounds such as efrapeptins [37] and tolypin [38], diketopiperazines [39], and the carboxysterol antibiotic ergokonin-C [39]. In addition to well-known core enzymes involved in producing fungal secondary metabolites (NRPSs, PKSs, prenyltransferases, and terpene cyclases (TC)), a large number of modifying enzymes such as racemases, methyltransferases, acetyl transferases, prenyltransferases, cytochrome P450 monooxygenases (P450s) and oxidoreductases are often required for synthesis of the final bioactive products. In fungi, these are often found clustered with the core enzymes to form secondary metabolite biosynthetic gene clusters [40], which some hypothesize may facilitate or be driven by horizontal transfer [41]. Others suggest clustering may minimize the number of coordinated interactions between regulatory elements [42]. We identified a total of 14 NRPSs, 20 PKSs, 4 Hybrid PKS/NRPSs, 11 putative NRPS-like enzymes, 5 putative PKS-like enzymes, and one dimethylallyl-tryptophan synthase (DMATS) in the *T. inflatum* genome (Table S3), indicating that *T. inflatum* has a large potential for secondary metabolite production. The majority of these core enzymes fell within one of the 36 secondary metabolite clusters identified by SMURF [43] or an additional 2 clusters (38 total) identified by antiSMASH [44].

In addition to the NPRS in the cyclosporin cluster, phylogenomic analyses of *T. inflatum* NRPS adenylation domains (Figure S3, Table S4) identified homologs of a number of functionally characterized NRPSs from fungi, including three peptaibol synthetases (TINF05969, TINF07827, TINF07876), both intracellular (*ChNPS2* - TINF08996) and extracellular (*ChNPS6* - TINF01764 and TINF06175) siderophore synthetases, and a homolog of conserved NRPS-like proteins involved in morphological development (*ChNPS10* TINF09755) (Figure S3, Table S4).

We also identified an NRPS (TINF02556) whose A-domains group with those of the ergot alkaloid synthetases (*cpps1-4*) from the grass endophyte *Claviceps purpurea* (Figures S3, S4A). The ergot alkaloid cluster in *C. purpurea* contains two trimodular NRPSs (*cpps1, cpps4*), two monomodular NRPSs (*cpps2, cpps3*), and a DMATS [45], [46]. The alkaloid secondary metabolite clusters recently discovered in the insect pathogens *M. robertsii* and *M. acridum* [19] contain homologs of the two monomodular NRPSs (*cpps2, cpps3*), the DMAT synthase, and the majority of modifying enzymes found on the 5′ end of the *C. purpurea* cluster. In contrast, the antiSMASH predicted cluster in *T. inflatum* lacks homologs of these monomodular NRPSs and other ergot alkaloid biosynthetic genes but contains a four modular NRPS (TINF02556) with A-domains that show closest similarity to the trimodular NRPSs (*cpps1* and *cpps4*) from *C. purpurea*, as well as other genes involved in secondary metabolism (Figure S4B). We also identified an additional cluster in *Metarhizium* spp. which contains a 7 modular NRPS (MAA_06559, MAC_08899) with A-domains that also show homology to *cpps1* and *cpps4*, but which also lacks other genes from the ergot alkaloid cluster (Figure S4B). *T. inflatum* does contain one DMAT synthase. However, it is located on a different scaffold in a distinct secondary metabolite cluster predicted by antiSMASH to be involved in terpene biosynthesis (Figure S4C). While further chemical data is needed, we conclude it is unlikely that the *T. inflatum* cluster produces an ergot alkaloid compound similar to those produced by *C. purpurea*.

### Evolution of Cyclosporin Synthetase (*simA*)

Cyclosporins are cyclic depsipeptides belonging to a class of cyclic undecapeptides [10], [47], and *T. inflatum* is known to produce 25 different analogs of cyclosporin (cyclosporins A-I and K-Z) [48], [49]. CsA is composed of 11 substrate molecules produced by an NRPS encoded by the single 45.8 kb *simA* locus, and like the products of many NRPSs, CsA contains several non-proteinogenic substrates including 2-aminobutyric acid, D-alanine, and (4*R*)-4-[(*E*)-2-butenyl]-4-methyl-threonine (Bmt) [10]. The *simA* gene displays a modular structure typical of NRPSs, consisting of 11 modules comprised of three core catalytic domains: adenylation (A), which binds and activates the substrate, thiolation (T), which attaches substrates to the NRPS, and condensation (C), which forms a peptide bond between adjacent substrates. Each module activates one of the eleven substrates and additional methylation (M) domains are present which methylate substrates 2 (Leu), 3, (Leu), 4 (Val), 5 (Bmt), 8 (Leu) and 10 (Leu). Various other fungi within Hypocreales (*Acremonium*, *Chaunopycnis*, *Fusarium*, *Isaria*, *Nectria*, *Neocosmospora*, *Trichoderma* and *Verticillium*) have been reported to synthesize a common profile of cyclosporins A-D and E-F [49], [50]. Only a few fungi outside of Hypocreales (*Aspergillus terreus*) [51] have been reported to produce cyclosporin A, while others (*Leptostroma*, *Cylindrotrichum*, *Stachybotrys*) produce a single and often novel cyclosporin-related compound [52], [53].

**Figure 4:**
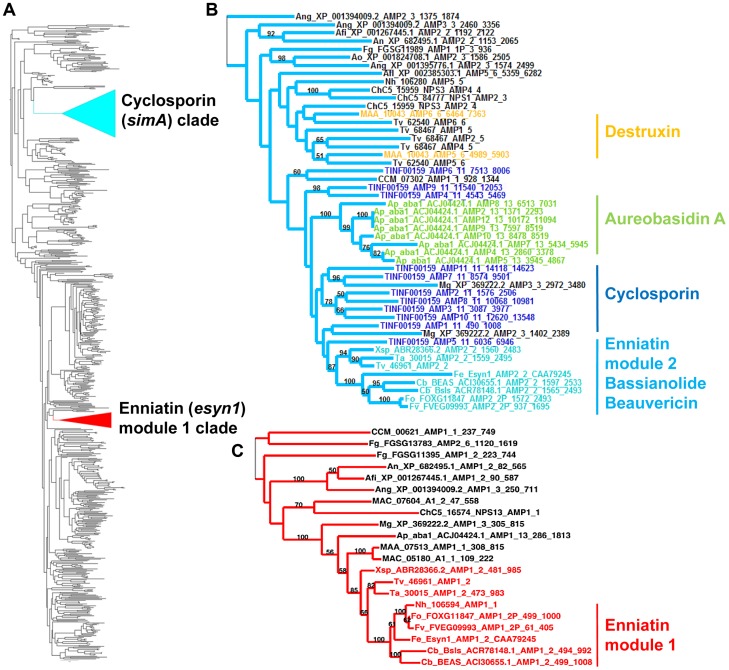
Phylogeny of cyclosporin synthetase (***simA*** **).** A) Locations of the cyclosporin (*simA*) and enniatin synthetase (*esyn1*) module1 clade showing their disparate locations in the larger NRPS A-domain phylogeny. B) Expanded view of the *simA* clade, showing modules from six other fungal NRPSs producing cyclic depsipeptides and containing A-domains in the *simA* clade: module 2 of enniatin synthetase (*esyn1*), module 2 of beauvericin synthetase (*bbBeas*), module 2 of bassianolide synthetase (*bbBsls*), aureobasidin A synthetase (*aba1*), and modules 5 and 6 of destruxin A synthetase (*dtxS1*). C) Expanded view of the enniatin synthetase (*esyn1*) module 1 clade containing module 1 of *esyn1*, *bbBeas*, and *bbBsls*.

A previous phylogenomic study of NRPSs from 37 complete fungal genomes found that *simA* grouped sister to a clade of bacterial NRPSs but found no complete (11 modular) homolog of *simA* in either bacteria or other fungi [54]. Similarly, we employed BLAST searches of the NCBI database and HMMER searches across closely related hypocrealean fungi and found no complete homologs of *simA* (Figures 4, S3, S5). The phylogenetic tree constructed from A-domain sequences of the top 50 BLAST hits to *simA* from the NCBI nr database (Figure 4, S5) showed that no bacterial sequences group within the *simA* clade. This suggests that *simA* likely evolved by duplication of modules within fungi rather than through recent horizontal transfer from bacteria. Individual adenylation domains from several other fungal NRPSs group within the *simA* clade with 100% bootstrap support (Figures 4B, S5). These include NRPSs synthesizing several other known fungal cyclic depsipeptides such as enniatin synthetase (*esyn1*) (*Fusarium equiseti*) [55], beauvericin (*bbBeas*) and bassianolide (*bbBsls*) synthetases (*Beauveria bassiana*), the NRPS responsible for biosynthesis of the antifungal compound aureobasidin A (*aba1*) (*Aureobasidium pullulans*), and two modules of destruxin synthetase (*dtxS1*) (*M. robertsii*) (Figures 4B, S5). These compounds share similar functions, having either anti-insect (beauvericin, bassianolide, destruxin, and cyclosporin A) [13], [56]–[58] and/or antifungal properties (aureobasidin A [59] and cyclosporin A [60]).

**Figure 5:**
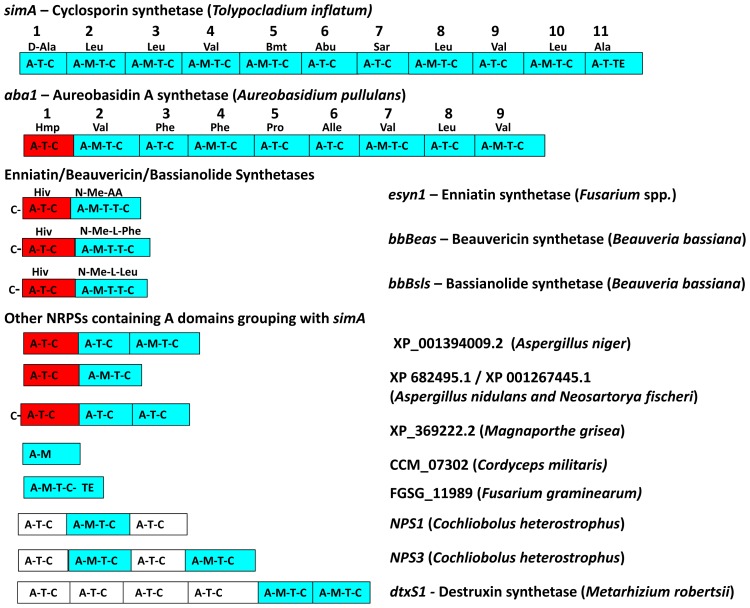
Modular domain structure and A-domain specificities of NRPSs grouping within the *simA* clade. Color coding of NRPS modules denotes clade assignment of A-domains in phylogeny (Figure 4, Figure S3): light blue = groups within cyclosporin (*simA*) clade, red = groups within enniatin (*esyn1*) module 1 clade, white = groups with *perA*-like outside both *simA* and enniatin module 1 clade (Figure S3). Abbreviations for unusual amino acid substrates: Bmt = (4*R*)-4-[(*E*)-2-butenyl]-4-methyl-threonine, Abu = Aminobutyric acid, Hiv = D-2-hydroxyvaleric acid, Hmp = D-Hmp, D-2-hydroxy-3-methylpentanoic acid.

Fungal NRPSs containing adenylation (A) domains found in the *simA* clade, share a complex history of evolution through duplication and fusion of modules [61]. For example, the A-domain of module 1 of the NRPSs synthesizing enniatin (*esyn1*), beauvericin (*bbBeas*), and bassianolide (*bbBsls*) synthetases all code for an identical non-amino acid substrate, D-2-hydroxyisovaleric acid (Hiv) and group together phylogenetically in a clade distinct from the *simA* clade (Figures 4A, C, 5, S5 [enniatin module 1 clade]). In contrast, the A-domain from module 2 of enniatin and the C-terminal modules of all of these genes fall within the *simA* clade (Figures 4A, B, 5, S5 [enniatin module 2 clade]), suggesting fusion of modules. Other fungal NRPSs from *Magnaporthe grisea* (XP_369222.2) and *Aspergillus* species (XP_001394009.2, XP 682495.1, XP 001267445.1) display a similar pattern. Others, such as *NPS1* and *NPS3* from *Cochliobolus heterostrophus* and destruxin synthetase from *M. robertsii*, contain two A-domains grouping in the *simA* clade, but remaining A-domains group with the *Epichloë festucae* NRPS *perA*, outside both the *simA* and the enniatin module 1 clades (Figures 5, S3).

The C-terminal modules of these same NRPSs, containing A-domains in the *simA* clade, show evidence of duplication of NRPS modules followed by divergence of A-domain substrate specificities. Enniatins, for example, are known to vary in the N-Me-amino acid incorporated by the second A-domain [62]. The C-terminal modules (2–8) of aureobasidin A synthetase (*aba1*) provide the clearest example of extensive module duplication and divergence within a single species as all A-domains group as a single monophyletic group with 100% bootstrap support and many share over 95% sequence similarity (Figure 4B) [63]. While the sequence of duplications within *simA* is more complex, A-domains of *simA* that group together with greater than 50% bootstrap support encode for identical substrate amino acid specificities ([A2, A3, A8, A10; bs = 78%) for Leu and (A4 and A9; bs = 98%) for Val]), suggesting these domains represent more recent duplications that have not diverged in specificity encoding regions (Figures 4B, 5). The average pairwise dN/dS ratio across all A-domains was low (ώ = 0.182), indicating that most sites within A domains are under purifying selection. However, the branch-site REL method of the HYPHY package [64] detected significant evidence of episodic positive selection (p<0.0001) on the branch separating the clade coding for Leu (A2, A3, A8, A10) from other A-domains. These results are consistent with a process in which duplication followed by lineage specific changes at a few amino-acid positions contributed to the evolution of the species-unique cyclosporin metabolite.

### Computational and Transcriptional Identification of the *simA* Biosynthetic Cluster

**Figure 6:**
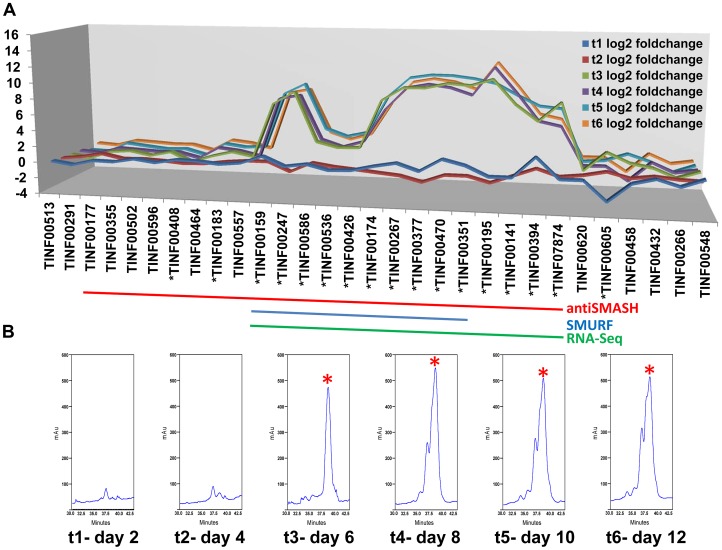
Computational and transcriptional identification of the cyclosporin metabolite cluster. A) Fold changes (log_2_ transformed) of gene expression levels from SDB to SM media at time points 1–6 (days 2, 4, 6, 8, 10, 12). Strong upregulation of gene expression occurs after time point 3 (day 6). All genes marked below with an * are upregulated with q-value<0.001 for at least one time point. The boundaries to the cyclosporin *simA* cluster predicted by antiSMASH (red), SMURF (blue), and RNA-Seq data (green) are indicated by bars below. B) Partial HPLC traces showing the major cyclosporin A peak at 38 min. (marked with a red asterisk) in SM medium for each harvest time point. Trace amounts of cyclosporin A are found at time points 1 and 2, but production spikes at time point 3 (day 6) and peaks at time point 4 (day 8). Additional peaks surrounding the 38 min. major peak are observed after time point 4, consistent with depletion of substrates in the culture media leading to relaxed specificity of NRPS A-domains and production of additional cyclosporin analogs.

**Figure 7:**
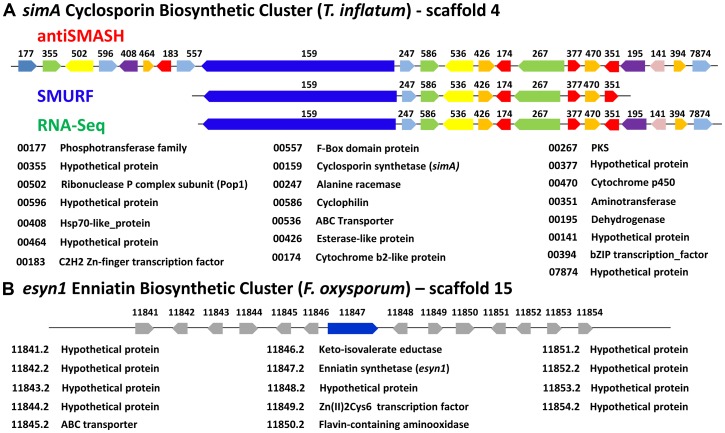
Organization of the *simA* biosynthetic cluster. A) The secondary metabolite gene cluster responsible for cyclosporin biosynthesis as identified by antiSMASH (red), SMURF (blue), and RNA-Seq (green) with predicted protein functions or families listed below. B) The enniatin biosynthetic cluster of *F. oxysporum* showing genes orthologous to those in the *simA* cluster in blue [only enniatin synthetase (*esyn1*)] and genes without orthologs in the *simA* cluster in grey.

The two computational methods used for defining secondary metabolite clusters, SMURF and antiSMASH, identified slightly different boundaries to the *simA* cluster (Figures 6, 7). The secondary metabolite gene cluster surrounding *simA* predicted by antiSMASH contains 22 genes and spans over 112 kb, while SMURF delineated a slightly smaller cluster of 10 genes spanning approximately 93 kb (Figures 6, 7).

In order to utilize transcriptional data to define the cyclosporin metabolite cluster, an RNA-Seq time course experiment in a cyclosporin-inducing medium (SM medium) [65] was conducted. Three biological replicates each were grown in the inducing (SM) and a rich control medium, Sabouraud Dextrose Broth (SDB), and were sampled at two-day intervals for a total of six time points (days 2, 4, 6, 8, 10, and 12). Total RNA isolated from these samples was prepared for RNA-Seq and remaining mycelia and culture filtrate was harvested and analyzed by LS-MS in order to correlate gene expression patterns with production of cyclosporin metabolites.

Expression profiling under cyclosporin-inducing conditions (SM medium) clearly identified a cluster of genes surrounding *simA* that were significantly (q-value<0.01) upregulated (Table S5) during production of cyclosporins as detected by an HPLC peak (Figures 6, S6) that included analogs with nominal molecular masses of 1188, 1202 (Cyclosporin A), 1216, and 1218 Da (data not shown). Upregulation of genes within the cluster became highly significant at time point 3 (day 6) which corresponded with the first detection of large quantities of cyclosporin in the culture filtrate by LC-MS and were consistently and strongly upregulated at time points 4, 5, and 6 that also showed an HPLC peak for CsA (Figure 6, S6, Table S5). The 5′ boundary of this cluster corresponded to the SMURF computational prediction beginning at *simA* (TINF00159), while the 3′ edge of this cluster corresponded to the 3′ boundary of the antiSMASH cluster prediction (TINF07874) (Figures 6, 7, Table S5).

LC-MS profiles for the cyclosporin-containing fraction were generated consistently from culture filtrate extracts obtained for each time point. Under the HPLC protocol used, analogs of cyclosporins eluted predominantly between 32–40 min, with the peak maximum for cyclosporin A (the major product, molecular mass 1202 Da) at 38 min. The overall production of cyclosporins peaked at time point 4 (day 8) in SM medium (Figure 6, Figure S6). Beginning at time point 4, an increase in the number of overlapping peaks for closely-eluting analogs of cyclosporin A is consistent with depletion of specific amino acids in the culture media leading to relaxed substrate specificity of the cyclosporin NRPS A-domains and production of distinct cyclosporin analogs (Figure 6, Figure S6). However, the existence of more than one isoform with the same molecular mass prevents the rigorous assignment of these metabolites (molecular masses 1188, 1202, 1216, and 1218 Da) by mass spectrometry alone.

This combination of computational and experimental approaches provided a more robust method for characterization of secondary metabolite clusters and demonstrates the utility of transcriptional data in confirming cluster boundaries.

### Organization and Components of the *simA* Cluster

Although NRPSs such as *simA* and *esyn1* contain A-domains with shared ancestry (Figure 4, S3, S5), the metabolite clusters containing these core metabolite genes do not share other homologous genes (Figure 7). Components of secondary metabolite clusters other than the core backbone enzymes function in synthesis of precursors, mediation of intermediate steps, transport and delivery, and modifications of the final metabolite [66]. While further functional studies (e.g. gene knockouts) are needed, the *simA* cluster contains genes which likely function in both synthesizing substrates for the NRPS and modification or activation of the cyclosporin product. The unusual non-proteinogenic amino acid substrate D-alanine must be supplied by an independent alanine racemase [67] as *simA* itself does not contain racemase activity. Similarly, it was shown that one of the unusual amino acid substrates of cyclosporin, (4*R*)-4-[(*E*)-2-butenyl]-4-methyl-threonine (Bmt), is synthesized by a polyketide biosynthetic mechanism [68]. As a cyclosporin mutant (Cyb56) was shown to accumulate Bmt [69], this substrate is likely synthesized by *T. inflatum*. The discovery of both a D-alanine racemase (TINF00247) and a PKS gene (TINF00267) within the cluster strongly suggests their involvement in production of these two unusual substrate molecules (Figure 7). The cluster also contains an aminotransferase (TINF00351), an enzyme involved in the synthesis of branched chain amino acids such as the Leu and Val residues found in cyclosporin. Several genes in the cluster belong to gene families commonly found in fungal secondary metabolite clusters, including a cytochrome P450 (TINF00470) and a dehydrogenase (TINF00195). Two transcription factors, a C2H2 zinc-finger transcription factor (TINF00183) on the 5′-edge of the cluster and a putative basic leucine zipper (bZIP) transcription factor (TINF0394) on the 3′-end of the cluster, are candidates for a cluster-specific transcriptional regulator (Figure 7).

Adjacent to the alanine racemase (TINF0247) is a gene (TINF00586) belonging to the cyclophilin family of peptidylprolyl isomerases (IPR002130) (Figure 7). The first isolated cyclophilin, human cyclophilin A (hCypA), was identified almost thirty years ago as the cellular target of cyclosporin [3]. Binding to hCypA is a prerequisite for the immunosuppressive activity of CsA as it causes CsA to undergo a conformational change to an entirely *trans* peptide conformation, which puts the calcineurin-binding motif of CsA in the reverse orientation compared to the crystal structure of CsA bound to a tetrapeptide substrate [70], [71] and primes CsA to create a better fit to its cellular target calcineurin. Cyclophilins have since been identified in nearly all kingdoms of life, including animals, plants, insects, fungi, protists, and bacteria, and they are classified based on their cellular location, domain organization and function [72], [73]. While different cyclophilins vary in their binding affinity for CsA, all exhibit petidyl isomerase activity that facilitates conformational changes from *cis* to *trans* at peptide bonds preceding prolines (peptidyl-prolyl bonds), and thus may function as general molecular chaperones in protein folding [74]. They are also implicated in diverse cellular processes including cell signaling, cell cycle control, intracellular transport, stress response, and virulence in both plant [75] and animal pathogens [76].

**Figure 8:**
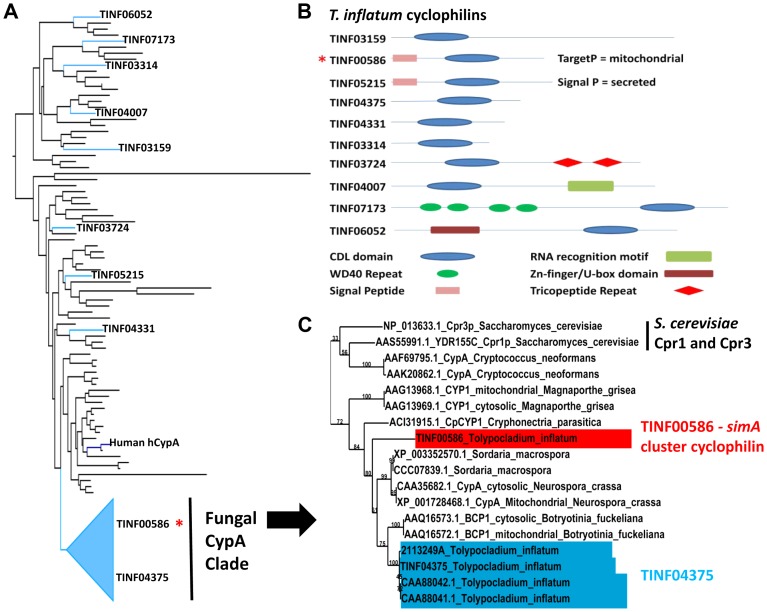
Cyclophilins in *T. inflatum* genome. A) Backbone of maximum likelihood phylogeny of major cyclophilins from *T. inflatum*, *H. sapiens*, *C. elegans*, *D. melanogaster*, and characterized cyclophilins from other fungi, bacteria, and protists (Figure S7) showing phylogenetic positions of the ten *T. inflatum* cyclophilins and the *simA* cluster cyclophilin (red asterisk) within the fungal CypA clade. B) Domain organization of cyclophilins identified in the *T. inflatum* genome. The cyclosporin cluster cyclophilin (TINF00586) is indicated with a red asterisk and contains a mitochondrial localization signal. C) Expanded view of the fungal CypA clade showing *S. cerevisiae* Cpr1 and Cpr3, the *simA* cluster cyclophilin (TINF00586) in red, and TINF04375 in blue. Note that all of the products in blue are likely produced by alternative splicing of the single gene TINF04375.

Using an HMM model to the conserved cyclophilin-like domain (CLD) of cyclophilins (Pfam: PF00160.16), we identified ten proteins, including TINF00586, containing the conserved CLD domain (PF00160.16) in the *T. inflatum* genome (Figure 8 A, B). The *simA* cluster cyclophilin (TINF00586) has highest and equally scoring BLAST hits to two *S. cerevisiae* proteins, Cpr1 (YDR155C) (e^−41^), the yeast homolog of hCypA, and the yeast mitochondrial cyclophilin Cpr3 (YML078W) (e-^41^), and it contains an N-terminal signal peptide with a localization signal to mitochondria (Figure 8B) [77]. In a phylogeny including the conserved CLD domains of major cyclophilins from other fungi, animals, bacteria, and protists, the *T. inflatum* cyclophilins group in diverse locations in the phylogeny, suggesting that *T. inflatum*, like other eukaryotes, contains a full suite of cyclophilins (Figures 8A, S7).

The *simA* cluster cyclophilin is similar in domain structure to hCypA and other cyclophilin A homologs. It groups phylogenetically with greater than 70% bootstrap support in a clade with another *T. inflatum* cyclophilin gene (TINF04375) and a number of fungal cyclophilins with roles in morphological development and pathogenesis in either plant (BCP1 (*Botrytis cinerea*) [78], CpCYP1 (*Cryphonectria parasiticus*) [75], and CYP1 (*Magnaporthe grisea*) [79]) or animal (Cpa1 and Cpa2 (*Cryptococcus neoformans*) systems [80] (Figures 8A, C [Fungal CypA Clade], S7). Several putative cyclophilins previously cloned from *T. inflatum*, including two that are hypothesized to be alternately spliced products of a single gene targeted to the cytosol and mitochondria, respectively [81], and another gene coding for an approximately 19.5 kDa protein (*cptA*) [82], are nearly identical in sequence and all group closely with TINF04375 (Figure 8C, S7). Alternative splicing of this single gene is consistent with the finding that other cyclophilin genes in this clade *CypA (N. crassa)* [83] and *BCP1* (*B. cinerea*) [78]), also produce alternately spliced mitochondrial and cytosolic isoforms (Figure 8C, S7). The *simA* cluster cyclophilin (TINF00586) is distinct in sequence from TINF04375 and groups at the base of this clade with the CpCYP1 of *C. parasiticus* (Figure 8C, S7).

While the direct mechanism of toxicity of CsA in insects remains unknown, histopathological changes consistent with Mitochondrial Pore Transition Permeability (MPT), such as swollen, electron-dense, and occasionally lysed mitochondria, have been observed in several insect species treated with CsA [84]. We hypothesize that the *simA* cluster cyclophilin may be involved in targeting CsA to the insect mitochondria. Other possible functions include a role in folding of the cyclosporin peptide during export, creation of a pre-activated CsA-CYPA cocktail prior to delivery to the host, protection of CsA from proteolysis by endopeptidases [85], binding to detoxifying proteins in hemolymph [86], or auto protection for *T. inflatum* against CsA toxicity.

### Transcriptional Responses in the *simA* Gene Cluster in Relation to Insect Pathogenesis

Toxins and other secondary metabolites are suspected to function in insect pathogenesis but their expression patterns and modes of action remain poorly characterized. Previous studies suggest that many secondary metabolites are expressed at very low levels under most experimental conditions [87], and their expression is elicited only in response to specific stimuli. Like many insect pathogenic fungi, *T. inflatum* exhibits a complex lifecycle encompassing a saprobic growth phase in soil and a pathogenic growth phase on and within the insect host (Figure 1). The pathogenic phase initiates with an infection phase that involves growth on, and penetration of, the insect cuticle. This infection phase is followed by a colonization phase, which initially involves a yeast-like (hyphal body) growth phase within insect hemolymph, and ultimately switches to a filamentous growth form that colonizes the insect to form an endosclerotium. Previous studies have shown that cyclosporin has immunosuppressive functions in insects [58], as well as in humans, suggesting a role for and expression of cyclosporin inside the insect. In order to evaluate the expression of the *simA* cluster in relation to insect pathogenesis, RNA-Seq was carried out on fungal cultures grown on (1) minimal medium supplemented with insect cuticle (infection stage), and (2) Grace's insect medium supplemented with insect hemolymph (colonization stage). Each of these treatment samples (cuticle and hemolymph) were compared to a control grown on SDB. Transcriptional responses in these media were compared qualitatively to responses in the cyclosporin-inducing (SM) medium.

**Figure 9:**
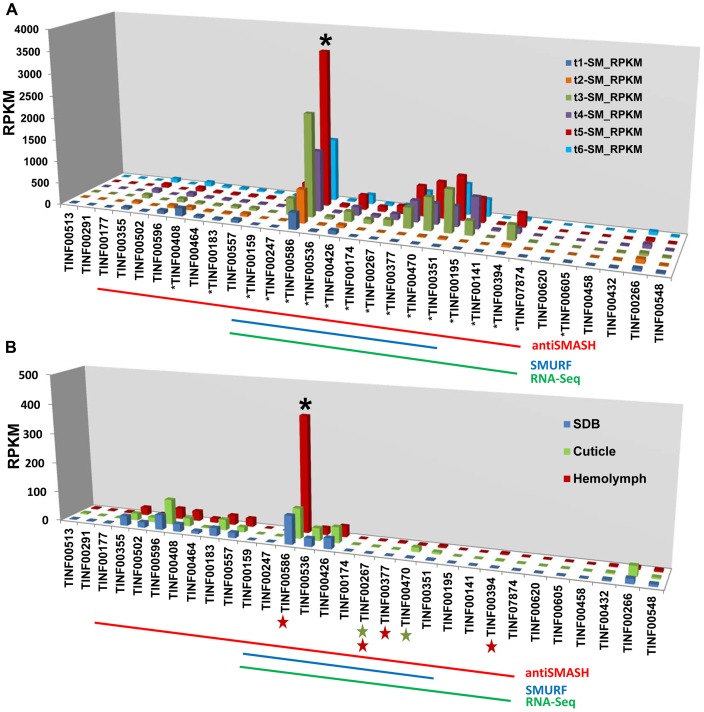
Expression profiling of genes in the *simA* cluster in relation to insect pathogenesis. A) Relative expression levels in Reads Per Kilobase per million mapped reads (RPKM) of genes in the *simA* cluster in SM medium. Significantly (p<.01) upregulated genes are shown with an asterisk below gene. B) Relative expression levels (RPKM) of genes in the *simA* cluster in Sabouraud Dextrose Broth (SDB), cuticle, and hemolymph media. Genes that are significantly (p<.05) upregulated are shown with either a green star (cuticle media) and/or red star (hemolymph media) below gene. In both A and B, the Y-axis is RPKM, the cluster cyclophilin (TINF00586) is indicated with an asterisk above the histogram bar and the antiSMASH (red), SMURF (blue), and RNA-Seq (green) predicted clusters are indicated by lines below.

In the strongly inducing SM medium, nearly all genes within the cluster were upregulated with extremely high significance (q-value<0.0005) (Figure 6, Table S5), but their relative expression levels varied widely (Figures 9A, S6, Table S5). The most highly expressed gene in the cluster was the cyclophilin gene (TINF00586). However, this gene also had relatively high levels of constitutive expression in the control SDB medium and underwent only a 3.18× log_2_ fold increase in expression during time point 5 (Figure 6, Table S5). In contrast, most other genes within the cluster, with the exception of TINF00536 and TINF00426, had very low levels of constitutive expression in control SDB medium, but were more highly upregulated in SM medium (Figures 6, 9A, S6, Table S5). For example, the PKS (TINF00267), the cytochrome P450 (TINF00470), the D-alanine racemase (TINF00247), the dehydrogenase (TINF00195), a hypothetical protein (TINF00377), and the aminotransferase (TINF00351) had over a 9× log_2_ fold increase in expression in SM medium compared to the control (Figure 6, Table S5). Several other genes, including *simA* (TINF00159), a cytochrome b-2-like protein (TINF0174), two hypothetical proteins (TINF00141 and TINF07874), and the bZIP transcription factor (TINF00394) experienced between 5–9× log_2_ fold increases in expression (Figure 6, Table S5). The C2H2 transcription factor on the 5′ end of the cluster (TINF00183) experienced less than 1× log_2_ fold increases in expression, suggesting that the bZIP transcription factor (TINF001374) is more likely the cluster-specific transcriptional regulator (Figure 6, Table S5).

While the *simA* NRPS itself was not significantly upregulated in media simulating stages of insect pathogenesis (cuticle and hemolymph media), several cluster genes showed significant upregulation (q-value<0.05) (Figure 9B, Table S6). In cuticle medium, the PKS gene (TINF00267) and the cytochrome P450 (TINF00470) were significantly upregulated (Figure 9B, Table S6). In hemolymph medium, a greater number of cluster genes including the PKS gene (TINF00267), the bZIP transcription factor (TINF00394), the cyclophilin homolog (TINF00586), and a hypothetical protein containing a thioester/thiol ester dehydrase-isomerase domain (TINF00377) were significantly upregulated (Figure 9B, Table S6). The P450 (TINF00470) was the most highly upregulated gene in the cuticle medium (3.25× log_2_ fold), while the bZIP transcription factor (TINF00394) was the most highly upregulated gene in hemolymph medium (3.58× log_2_ fold) (Table S6). Importantly, the cyclophilin gene (TINF00586) showed significant upregulation (1.82× log2 fold) only in hemolymph media and had the highest relative expression level of all cluster genes in hemolymph media (Figure 9B, Table S6).

However, culture media conditions can only simulate conditions of insect pathogenesis, and differential expression cannot be solely attributed to the added insect components as differences in the composition and pH of the basal media used may also influence expression patterns. RNA samples were also harvested 24 hours after transfer from a rich SDB medium to hemolymph medium. Given that full induction of cyclosporin production took nearly 6 days in a strongly inducing medium, it is possible that the weaker response observed in media containing insect components reflects an early stage of cyclosporin induction. The larger number of significantly upregulated genes and the strong upregulation of the bZIP transcription factor (TINF00394) only in hemolymph media, however, suggests a possible role for cyclosporin and the cyclophilin homolog during the colonization phase inside an insect host (Figure 9B, Table S6).

### Evolution and Synteny of the *simA* Cluster

**Figure 10:**
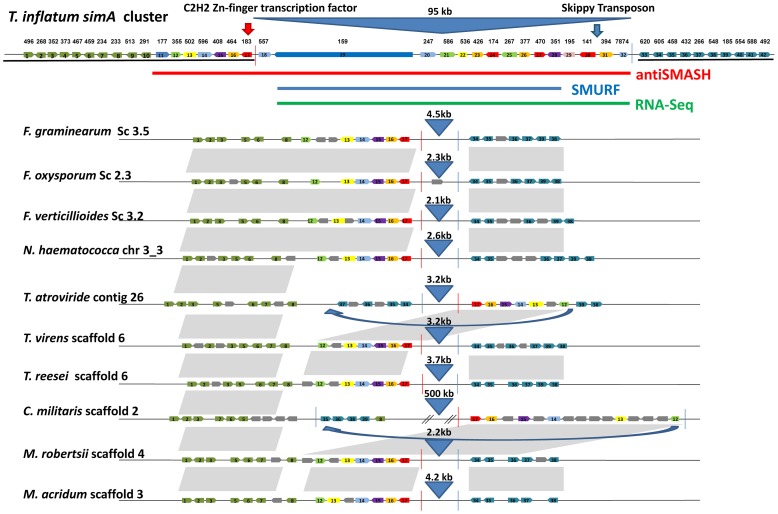
Synteny of regions flanking the cyclosporin cluster in other hypocrealean taxa. Genes within the cyclosporin biosynthetic cluster as delineated by antiSMASH (red), SMURF (blue), and RNA-Seq (green) plus ten genes on the 5′ (green) and 3′ (blue) flanks of the antiSMASH predicted cluster are shown at top and numbered from left (5′) to right (3′) (1–42). Orthologous genes in other hypocrealean taxa identified by best-pairwise BLAST searches are shown below for each species. Grey genes indicate additional genes present in other species while grey shaded areas show regions of synteny between genomes. Genes in *T. inflatum* in the region between the C2H2 Zn-finger transcription factor (TINF00183) on the 5′end (red line) and the RNA-Seq predicted 3′- end of the *simA* cluster (at TINF007874) (blue line) mostly lack orthologs in other hypocrealean genomes. The few best-pair orthologs identified in other Hypocreales were found elsewhere in these genomes. Numbers above blue triangles show length of intervening sequence between the 5′ (red line) and 3′ (blue line) flanks of the cluster (approximately 95 kb in *T. inflatum*) which is less than 5 kb in all other hypocrealean taxa except *C. militaris* and *Tr. atroviride*. Blue arrows show regions inverted in *Tr. atroviride* and *C. militaris*. In *Tr. atroviride*, an inversion has occurred but the region between adjacent genes is still <5 kb and contains no additional genes. In *C. militaris*, the inversion has added nearly 500 kb of sequence containing additional genes, none of which were found to have orthologs in the *simA* cluster or to belong to other secondary metabolite clusters.

The disjunct distribution of cyclosporin biosynthesis across fungal taxa poses interesting questions about the origins and evolution of the cyclosporin biosynthetic cluster. In order to search for homologs of cyclosporin cluster genes in other fungi, the top 25 BLAST hits to the antiSMASH predicted *simA* cluster genes plus 10 flanking genes on either side in the NCBI nr database were aligned and phylogenies constructed using maximum likelihood (Figure S8). Pairwise BLASTP searches among the set of the fourteen hypocrealean fungi analyzed for phylogenomic analyses were also performed to identify reciprocal best-pair hits between these genomes and these best-pair BLASTP hits were considered as orthologs. These analyses revealed that only one gene (TINF00195) between the C2H2 transcription factor (TINF00183), adjacent to the 5′ end of the RNA-Seq defined cluster (Figure 10, red line), and the 3′-end of the RNA-Seq defined cluster (Figure 10, blue line, at TINF07874) had hits above e^−05^ to bacterial genes (Figure S8). Similarly, only a few genes (TINF00557, TINF00586, TINF00426, TINF00174, TINF00267, TIN00470, and TINF00394) had orthologs in other sequenced hypocrealean taxa (Figures 10, S8). In contrast, most genes flanking this region on both sides, as well as the 8 genes at the 5′-end of the antiSMASH predicted cluster (TINF00177 to TINF00557) contained single-copy orthologs in nearly all other hypocrealean taxa that showed relatively conserved synteny with those in *T. inflatum* (Figures 10, S8). The 8 genes on the 5′ end of the antiSMASH predicted cluster that had homologs in other Hypocreales were excluded from the cluster by both SMURF and the RNA-Seq predicted cluster (Figures 6, 7). Additionally, the few homologs of genes within the RNA-Seq defined cluster in other hypocrealean taxa were scattered elsewhere in these genomes and not located between these conserved flanking regions (Figure 10, S8). In most species, no additional genes were found between the 5′ flank (TINF00183) and the 3′ flank (TINF07874) of the RNA-Seq defined cluster and the intervening region between these boundaries was less than 5 kb (Figure 10). *F. oxysporum* contained a single additional gene in this region, while *C. militaris* underwent an inversion that added additional genes, none of which were orthologs of the *simA* cluster genes (Figure 10). These results suggest one of two hypotheses regarding the origin of the nearly 100 kb *simA* cluster region in *T. inflatum* that is missing in other Hypocreales: 1) the cluster has been horizontally transferred from another fungal species into this site in *T. inflatum*, or 2) this region has evolved by recruitment of genes from other regions of the *T. inflatum* genome.

Horizontal transfer of complete secondary metabolite clusters between heterologous bacterial species by transposition has been demonstrated [88], and evidence exists for horizontal transfer of large secondary metabolite clusters among fungi [89], [90]. However, BLAST searches did not detect a homologous cluster in other fungal taxa. To further test for horizontal transfer, we utilized the customized pipeline (CRAP) [89] to scan for syntenic homologs of *simA* cluster genes in 195 sequenced fungal genomes. We did not detect a cluster containing both the NRPS and PKS genes and a majority of accessory genes from the *simA* cluster. While these results do not preclude the existence of a nearly complete cluster in a yet to be sequenced fungus, available evidence suggests that the cluster more likely evolved by a process of recruitment of genes into the cluster from elsewhere within the *T. inflatum* genome.

The fact that *simA* and *esyn1* synthetases clearly share related A-domains while the clusters containing these NRPSs lack other shared genes leads us to hypothesize that these clusters have evolved by recruitment of distinct modifying enzymes into regions surrounding these core NRPSs through rearrangement and transposition. Transposons have been found adjacent to other secondary metabolite gene clusters in fungi, such as the gliotoxin cluster in *Aspergillus fumigatus* [91]. While their role in shaping the evolution of secondary metabolite clusters remains speculative, they represent a potential mechanism for gene recruitment. A *gypsy* LTR retrotransposon, related to *F. oxysporum* SKIPPY [92], was found on the 3′ end of the *simA* cluster (Figure 10), and several lines of evidence suggest that the region surrounding the *simA* cluster may be prone to rearrangement in other taxa. Both *Tr. atroviride* and *C. militaris* show evidence of an inversion or transposition in this region of the genome. In *Tr. atroviride*, this is simply an inversion which did not add additional genes. In *C. militaris*, an additional 0.5 Mb of sequence distinct from the *T. inflatum* sequence is found between the two rearranged flanking regions, but this additional sequence does not contain any homologs of *simA* cluster genes nor is it predicted by SMURF or antiSMASH to contain other secondary metabolite clusters (Figure 10).

### Conclusions

Although the *simA* clade itself was shown previously to group sister to a clade of bacterial NRPSs [54], no bacterial homolog of *simA* were found within the *simA* clade. Thus, we conclude that *simA* evolved through duplication and divergence of fungal NRPS modules within *T. inflatum* rather than by recent horizontal transfer from bacteria (Figures 4, S5, S8). A number of related NRPSs that share homologous A-domains with *simA*, including enniatin, beauvericin, bassianolide, and aureobasidin A synthetases, evolved through a similar process of module duplication, but also fusion of distantly related NRPS modules (Figures 4, 5, S5). Interestingly, all of these metabolites possess insecticidal or fungicidal properties. Other genes within the secondary metabolite gene clusters containing these NRPSs, however, do not show homology with those in the *simA* cluster and are not syntenic with the *simA* cluster. Regions syntenic with the *simA* cluster in other hypocrealean fungi instead lack the nearly 100 kb of the *simA* cluster present in *T. inflatum*. However, searches for orthologs of the *simA* cluster genes in other fungi using BLAST searches of the NCBI nr database and the CRAP pipeline found no clusters containing more than a few genes showing similarity to those in the *simA* cluster. While horizontal transfer from a yet un-sampled fungus cannot be ruled out, we suggest that the *simA* cluster instead had a lineage specific origin, having evolved through recruitment of genes from other locations in the *T. inflatum* genome.

The discovery of a homolog of hCypA, the cellular target of cyclosporin in mammalian systems, within the *simA* cluster of *T. inflatum* is novel. This is the first report of a cyclophilin gene located within a secondary metabolite gene cluster, although several genes shown to be dependent on the activity of calcineurin, the target of CsA, are suspected of being located within a secondary metabolite biosynthetic cluster in *B. cinerea* [78]. The up-regulation of the cluster cyclophilin in hemolymph and high expression levels under both inducing conditions and in insect hemolymph medium suggests a role for this gene in mediating the activity of cyclosporin *in vivo*. Elucidation of the *simA* gene cluster through a combination of computational and transcriptional approaches opens the door for functional studies and chemical analyses to address mechanisms of action in nature and potential novel applications of these compounds in pathogenicity and medicine.

## Materials and Methods

### Strains and Culture Conditions

*Tolypocladium inflatum* NRRL 8044, the original strain from which cyclosporin was isolated, was obtained from NRRL. Cultures were grown for 2.5 weeks on cornmeal agar to induce sporulation. For DNA extractions, conidia from agar plates were used to inoculate potato dextrose broth cultures, which were grown for 3 days before harvesting.

### DNA Isolation and Sequencing

Lyophilized mycelia were ground in liquid nitrogen and genomic DNA was isolated using the Qiagen genomic tip 500 following the manufacturer-supplied protocol for isolation of genomic DNA from plants and filamentous fungi (Qiagen). A 350 bp insert Illumina library was prepared by shearing two samples of approximately 5 µg of genomic DNA in a Biorupter XL sonicator for 20 min with a cycle of 30 sec on and 30 sec off. These samples were pooled and prepared following the Illumina protocol for paired-end sequencing. Illumina sequencing was performed on the Illumina GAII machine at the Center for Genome Research and Biocomputing (CGRB) at Oregon State University. For 454 sequencing, a shotgun library and a 3-kb paired end 454 library were each prepared and sequenced on a full plate using titanium chemistry at the Duke IGSP Sequencing Core Facility at Duke University.

### Genome Assembly

454 reads were assembled using the Newbler Assembler version 2.3 (454 Life Sciences) using a combined shotgun and 3 kb paired end library assembly. Out of a total of 4,260,863 input 454 reads (1,516,236 single end shotgun reads, 1,067,664 mate pair reads with both pairs, and 1,676,963 singleton mate pair reads), 4,077,744 (95.70%) were assembled into the Newbler Assembly. Illumina reads were first trimmed to 50 bp, and sequences containing adaptors, N's, or greater than 2 bp with a quality score below 20 were filtered out of the dataset. A total of 37,643,435 quality filtered reads, containing 33,586,130 paired end reads, were submitted to SOAP Gapcloser [93] to fill gaps in the Newbler scaffolds. Finally, a mapping assembly was performed in MIRA to map the quality filtered Illumina reads to the SOAP Gapcloser assembly to correct for homopolymer base pair errors.

### Gene Predictions

Genome annotations were created in MAKER 2.00 [23] using three *ab initio* gene prediction models: an AUGUSTUS model trained for *F. graminearum*, a GeneMark model trained for *T. inflatum* via self-training, and a SNAP model trained for *F. graminearum*. Protein datasets from *F. graminearum*, *Nectria haematococca*, *Tr. reesei*, *Tr. virens*, and *M. robertsii* were submitted to MAKER as protein evidence. ESTs from *C. militaris*, *B. bassiana*, *M. robertsii*, and *Tr. reesei* downloaded from GenBank were included as EST evidence. Illumina PE RNA-Seq reads from *T. inflatum* PDB grown cultures (see RNA isolation and sequencing) were trimmed to 70 bp and filtered to remove sequences containing adaptors, N's, or greater than 2 bp with a quality score below 20, and assembled into transcripts using OASES with a coverage cutoff of 3 [94]. Assembled transcripts were input into MAKER as EST evidence. Transfer RNAs (tRNAs) were identified with tRNA scan-SE [95].

### Repeat Elements

Repeat elements were identified with Repeat Masker (Smit, AFA, Hubley, R & Green, P. *RepeatMasker Open-*3.2.8 1996–2010 http://www.repeatmasker.org) using the fungal transposon species library (database version 20090604) as input and crossmatch version .990329. The number of CPA (NCBI Accession AM990997.1) and *Restless* (NCBI Accession Z69893.1) elements was identified in hypocrealean taxa by supplying these sequences as a library to Repeat Masker using the same settings.

### Phylogenetic Relationships and Orthologous Clusters

Proteins for the set of hypocrealean taxa utilized for functional annotations, as well as three Sordariomycete outgroups (*N. crassa, V. dahliae, and V. albo-atrum*), were clustered using MCL [30] at inflation parameter 3 within the Hal pipeline [31] and then filtered to identify single-copy orthologs. Single copy orthologs were aligned separately using MUSCLE [96] and then concatenated. The concatenated alignment was used to infer a phylogeny using RAxML with the best fit model of amino acid substitution for each gene partition estimated by ProtTest [97] and branch support estimated from 1000 bootstrap replicates. In a second set of analyses, amino acid rate categories were estimated across eight categories using PAML [98] and the effect of amino acid positions with high rates of substitution was determined by repeating the same RAxML analyses without the 6^th^, 7^th^, and 8^th^ rate categories. The ultrametric tree used for CAFÉ analyses was computed in r8s [99] from the phylogeny inferred by the first RAxML analysis using previously estimated divergence dates for Hypocreales [16] (Table S2). The number of clusters and proteins within clusters were mapped to nodes using a custom perl script.

### Functional Annotation

A functional annotation pipeline was developed for the hypocrealean fungi *F. graminearum*, *F. oxysporum*, *F. verticillioides*, *N. haematococca*, *Tr. reesei*, *Tr. virens*, *Tr. atroviride*, *M. robertsii*, *M. acridum*, *C. militaris*, and *T. inflatum*. Functional annotations were classified with InterproScan (http://www.ebi.ac.uk/interpro) using hmmpfam, PatternScan, ProfileScan and blastprodom databases and converted to GO with the Interpro2GO mapping (version 11/05/2011). Orthologous groups of proteins for these eleven fungi as well as the model fungi *S. cerevisiae*, *Schizosaccharomyces pombe*, and *N. crassa* were determined with Inparanoid [100], [101]. GO enrichments were performed by transferring additional GO annotations associated with proteins from model fungi to uncharacterized proteins within the same orthologous cluster. Protein localization signals excluding the plastid location were identified using TargetP [102] and Predotar [103] while transmembrane proteins were characterized using TMHMM [104]. Carbohydrate active enzymes (CAZymes) were determined by BLAST searches against the CAZymes database with a cutoff of <e^−30^. Proteases were identified by BLASTP searches of the MEROPS [105] database (http://merops.sanger.ac.uk/) with default settings (cutoff of e^−05^), and P450 enzymes were identified as BLAST hits to entries in the Nelson P450 [106] database below a cutoff of <e^−20^. The program CAFÉ, using the default settings, was used to analyze gene families (CAZymes, P450s, and proteases) expanded or contracted in taxa within Hypocreales.

### Secondary Metabolite Characterization

NRPS and PKS secondary metabolite genes and their domain structures were characterized using three methods: 1) HMMER searches using models build for Adenylation (A), Thiolation (T), and Condensation (C) domains of NRPSs [54], 2) SMURF [43], and 3) antiSMASH [44]. Adenylation (A) domains from NRPSs and ketosynthase (KS) domains from PKSs were extracted from the same eleven hypocrealean taxa included in the functional annotation pipeline and aligned together with known NRPSs from fungi and several outgroup A-domains (Acetyl CoA Synthetases, AcylCoA ligases, Ochratoxin, and CPS1) using MAFFT [107]. The alignment was examined and manually edited to remove regions of ambiguous alignment and a maximum likelihood phylogeny constructed in RAxML [108] using 1000 bootstrap replicates and the best fit model identified by ProtTest [97] (REVF plus gamma for A domains). The *simA* NRPS and the PKS gene found within the cyclosporin cluster were also subjected to BLAST against the NCBI nr database and A-domains from the top 50 hits were extracted, aligned, and analyzed as described above to identify any putative bacterial homologs of *simA*. The identified secondary metabolite genes were placed into secondary metabolite gene clusters using SMURF and antiSMASH.

### Synteny of the Cyclosporin Cluster

Protein sequences from the *T. inflatum* cyclosporin cluster plus ten flanking genes were used to search the genomes of related hypocrealean fungi using TBLASTN and BLASTP searches. The TBLASTN searches identified genes flanking the cyclosporin cluster that localized to the same contig or scaffold in other species. Genome-wide best-pair BLASTP searches were used to refine this search and identify those genes in the *simA* cluster with best pairwise BLASTP hits in other genomes and these were considered putative orthologs. The genomic sequence was also aligned with MAUVE at the DNA level to confirm synteny relationships (data not shown) [109]. To identify potential homologs in fungal species other than hypocrealean taxa and to address phylogenetic evidence for horizontal transfer, all *T. inflatum* cyclosporin cluster genes plus ten flanking genes of the antiSMASH predicted cluster were subjected to BLASTP against the nr database and the top 25 hits were extracted and aligned with MAFFT [107]. These alignments were filtered to remove regions of poor alignment using Gblocks (with relaxed setting), and ProtTest [97] was used to identify best-fit models for each gene alignment. Maximum likelihood phylogenies were constructed using RAxML [108] with the corresponding best-fit protein model and 100 bootstrap replicates. The *simA* cluster genes were also run through a customized pipeline (CRAP) [89] to search 195 sequenced fungal genomes and 1150 bacterial genomes for syntenic homologs of the *simA* cluster.

### RNA Isolation and Sequencing

For initial coverage of the transcriptome, conidia from 2 1/2 week-old cultures grown on cornmeal agar were adjusted to a concentration of 1×10^7^ spores/mL and 1 mL was used to inoculate a 100 mL liquid culture of potato dextrose broth. Mycelia were harvested after three days, flash frozen and ground in liquid nitrogen. RNA was extracted using the Qiagen RNAeasy kit. cDNA was prepared using the Mint cDNA Kit (Evrogen), sheared by nebulization for 6 min at 34 psi, prepared using the Illumina protocol for PE sequencing, and sequenced in one lane of a paired end 80 bp run on the Illumina GAII. For the RNA-Seq time course experiment in cyclosporin inducing (SM medium), two 200 mL flasks containing 100 mL of YM medium (4 g yeast extract, 20 g malt extract in 1L ddH20) were each inoculated with approximately 1 mL of 1×10^7^ conidia/mL. After two days growth in YM medium, 10 mL of the YM culture was transferred to 125 mL of SM production media or Sabouraud Dextrose Broth (SDB) in 200 mL flasks. All cultures were grown at 21°C. Three flasks (3 biological replicates) for each treatment (SM or SDB) were harvested every two days for a total of six time points (days 2, 4, 6, 8, 10, 12). Tissue was flash frozen and ground in liquid nitrogen and total RNA extracted in TRIzol®. Remaining tissue and culture filtrate was frozen at −20°C until chemical extraction for HPLC analyses. The RNA samples were prepared using the Illumina TruSeq RNA sequencing kit, randomized, and sequenced across three lanes of a 50 bp SE run on the Illumina HiSeq2000. For RNA-Seq expression studies in insect media, cultures were grown under three conditions: 1) a rich medium of Sabouraud dextrose broth (SDB), 2) a minimal salts medium [110] supplemented with 10% (w/v) black vine weevil (*Otiorhynchus sulcatus*, Coleoptera) insect cuticle cleaned from soft tissue with sodium tetraborate [111] to simulate the infection stage, and 3) Grace's insect medium (Gibco, unsupplemented) with 10% (v/v) filter sterilized black vine weevil hemolymph added to simulate the colonization stage. The minimal salts medium utilized for cuticle media contained 0.02% KH_2_PO_4_, 0.01% MgSO_4_, 0.2 p.p.m. FeSO_4_, 1.0 p.p.m. ZnSO_4_, 0.02 p.p.m. NaMoO_4_, 0.02 p.p.m. CuSO_4_, 0.02 p.p.m. MnCl_2_ adjusted to pH 6.5 These cultures were grown in a 2-stage fermentation that has proven a reproducible method for eliciting expression of proteins in response to specific elicitors in insect pathogenic fungi [112]. Conidia from cultures grown on cornmeal agar for 2.5 weeks were used to inoculate a 100 mL culture of rich media (SDB) at a final concentration of approximately 1×10^6^ conidia/mL. These cultures were grown on a shaking incubator for 48 hours, washed in sterile water, and approximately 500 mg wet weight of mycelia transferred to three replicates of 2 mL cultures for each media condition. Mycelia were grown for an additional 24 hours before harvesting. All cultures were grown at 21°C. RNA was extracted with TriZol® according the manufacturers protocol (Invitrogen), polyA RNA isolated using the Ambion PolyA Purist kit, and cDNA prepared using the Superscript III kit and random hexamer primers (Invitrogen). A 450 bp insert library was prepared for each biological replicate according to the Illumina PE protocol and all samples were multiplexed in each of three lanes of a SE 40 bp run.

### RNA-Seq Analyses

Barcodes were trimmed from 40 bp Illumina reads to a length of 36 bp for the insect pathogenesis experiment and the first and last nine bases were trimmed from the 50 bp reads based on quality score profiles to a length of 40 bp for the cyclosporin inducing experiment. Reads were mapped to gene models using GENE-Counter [113]. Differential expression was analyzed in GENE-Counter, which utilizes a negative binomial model and the NBP-Seq R package to model differential gene expression [114]. For the time course experiment, the three biological replicates in SM media were compared with the three biological replicates in the SDB media at each time point separately. For the insect assays, pairwise comparisons of SDB vs cuticle medium and SDB vs hemolymph medium were performed in GENE-Counter. Q-values and fold changes (log_2_ transformed) were calculated using the normalized expression values from NBP-Seq. Relative expression levels were calculated as Reads Per Kilobase of transcript per Million mapped reads (RPKM) [115].

### Chemical Extraction and HPLC/LC-MS Profiling

Culture filtrates from three biological replicates at each time point were pooled for chemical extraction. Each culture filtrate was applied to a glass column containing Diaion® HP20 resin (20 g, Supelco), which had been sonicated in MeOH (to de-gas) and then pre-washed with H_2_O (200 mL). The column loaded with sample was then eluted sequentially with H_2_O (200 mL, to desalt the sample), MeOH (100 mL) and acetone (100 mL). The latter two organic solvent eluents were combined and concentrated to provide an organic extract from each culture filtrate. In each case, the organic extract was applied to a C_18_ reversed-phase solid phase extraction (RP_18_ SPE) cartridge (10 g), which had been primed in 100% methanol, and then equilibrated in 70% methanol in water. The SPE cartridge was then eluted sequentially with 70% and 100% methanol in H_2_O before being washed with dichloromethane. The cyclosporin-containing SPE fractions (100% methanol, determined by direct injection MS) from each SM culture filtrate extract were selected for comparative HPLC (used to establish protocols for peak collection) and also LC-MS profiling, alongside the corresponding control SDB medium extracts. HPLC of each sample (50 µg per 5 injection) was performed using a linear gradient from 60–100% methanol in H_2_O over 40 min followed by isocratic 100% methanol for 20 min (column: Synergi Hydro-RP, 4.6×250 mm, 0.6 mL/min). LC-MS of 5 µg-containing aliquots was performed under identical HPLC solvent conditions using a Synergi Hydro-RP, 2×100 mm column with a flow rate of 0.2 mL/min.

HPLC was performed on a Shimadzu HPLC system comprising a SIL-20AC autosampler, dual LC-20AD solvent pumps and a SPD-M20A UV/VIS photodiode array detector. LC-ESI(+) MS data were obtained using an AB SCIEX QTrap 3200 mass spectrometer interfaced with a Shimadzu Prominence HPLC system. HPLC-grade solvents were used for all chemical extraction and fractionation.

### Accession Numbers

The Whole Genome Shotgun projects have been deposited at DDBJ/EMBL/GenBank under the accession number AOHE00000000.

## Supporting Information

Figure S1 The *MAT1-2* Mating Locus of *T. inflatum* NRRL 8044. Only a single mating type was found in this strain, indicating that the fungus is likely heterothallic.
(PDF)

Figure S2 GO-Slim profiles (*Aspergillus* GO-Slim) for 14 hypocrealean taxa analyzed and for species-unique genes in the insect pathogens (Figure 2). A, C, E - GO Slim profiles for hypocrealean taxa; A) molecular function, C) biological process, and E) cellular component categories. Taxa from inside of circle to outside of circle are *F. oxysporum, F. verticillioides, F. graminearum, N. haematococca, Tr. atroviride, Tr. reesei, Tr. virens, C. militaris, M. acridum, M. robertsii, and T. inflatum*. B, D, F - GO Slim profiles for species-unique genes in (from inside to out) *C. militaris*, *M. robertsii*, *M. acridum*, and *T. inflatum* for B) molecular function, D) biological process, and F) cellular component. Percent of genes in each category out of total annotated genes analyzed is shown.
(PDF)

Figure S3 Maximum likelihood phylogeny of 696 NRPS A-domains from 14 hypocrealean taxa showing previously characterized groups of NRPSs or those with known chemical products: Ch *NPS11*/gliotoxin (dark orange), Ch *NPS12* (dark blue), PKS-NRPS hybrids (blue green), ACV synthases (yellow green), NRPS-PKS hybrids (lavender), alpha-aminoadipate reductases (light pink), Ch *NPS10* (yellow), *simA/*cyclosporin clade (turquoise), enniatin synthase (*esyn1*) module 2 (red), Ch *NPS2* intracellular siderophore synthases (brown), enniatin synthase (*esyn1*) module 1 (red), *tex1*/peptaibols (dark purple), duplicated paralogous copies of Ch *NPS6* (Ch *NPS6*_1- pink and Ch *NPS6_2* - purple), *perA-*like/peramine (light orange), Ch *NPS8*/insect expanded clade (brick), and *cpps1-4*/ergot alkaloids (bright pink). Phylogeny constructed by maximum likelihood in RAxML using the PROTGAMMARTREV model and 1000 bootstrap replicates.
(PDF)

Figure S4 Possible homologs of ergot alkaloid biosynthetic genes in *T. inflatum* and *Metarhizium* spp. A) NRPS A-domains in other Hypocreales related to *C. purpurea* ergot alkaloid synthetases *cpps1-cpps4* and belonging to the ergot alkaloid clade in the larger phylogeny (Figure S3). The *C. purpurea* monomodular NRPSs (*cpps2* and *cpps3*) are shown in light green while the trimodular *cpps1* and *cpps4* are shown in dark green. Both *M. robertsii* (red) and *M. acridum* (pink) contain orthologs of the two monomodular NRPSs (*cpps2* and *cpps3*) as well as a novel 7 modular NRPS showing closer similarity to *cpps1* and *cpps4*. *T. inflatum* (orange) lacks both monomodular NRPSs but contains an NRPS with 4 modules whose A-domains also are most similar to *cpps1* and *cpps4* (orange). B) The antiSMASH predicted secondary metabolite gene clusters containing these NRPSs show that in addition to orthologs of the two monomodular *C. purpurea* NRPSs *cpps2* (MAA_06742 and MAC_06982) and *cpps3* (MAA_06744 and MAC_06980) both *M. robertsii* and *M. acridum* also contain orthologs (indicated by vertical lines and color coding – same color = homologs, black = not homologs) of the majority of genes found on the 5′ end of the *C. purpurea* ergot alkaloid cluster (shaded light green). Two 7 modular NRPSs in *Metarhizium* spp. (MAA_06559 and MAC_08899) that share homology to A-domains of the 3 modular genes (*cpps1* and *cpps4*) in *C. purpurea* are located in a distinct cluster that does not contain other genes from the ergot cluster. *T. inflatum* appears to lack homologs of the two monomodular NRPSs (*cpps2*, *cpps3*) and other genes in the 5′portion of the cluster but contains a single 4-modular NRPS (TINF02556) containing A-domains that group with *C. purpurea* NRPSs *cpps1* and *cpps4*. The antiSMASH predicted cluster containing TINF02556 is shown and contains other genes of unknown function predicted to be involved in secondary metabolism but lacks homologs of the ergot alkaloid biosynthetic pathway. C) A single DMAT enzyme is found in the *T. inflatum* genome, but it is located in a separate antiSMASH cluster predicted to be involved in terpene synthesis and located on a different scaffold from the NRPS cluster.
(PDF)

Figure S5 Phylogeny of A-domains from top 50 BLAST hits to the *simA* NRPS in the NCBI nr database. Although the *simA* clade (turquoise) groups near a large clade of bacterial NRPSs (orange), no bacterial NRPSs were found within the *simA* clade. Phylogeny constructed by maximum likelihood in RAxML using the PROTGAMMARTREV model and 100 bootstrap replicates.
(PDF)

Figure S6 A) left panel: relative expression levels (reads mapped to genes/total mapped reads in treatment) of each biological replicate in SM medium at each time point, right panel: complete HPLC traces of extracts from pooled samples at same time points in SM medium showing cyclsoporin A peak at 38 min. (marked by a red asterisk); B) left panel: relative expression levels (reads mapped to gene/total mapped reads in treatment) of each biological replicate in SDB medium at each time point and right panel: complete HPLC traces of extracts from pooled samples at same time point in SDB media showing only trace amounts of cyclosporin A in the peak at 38 min. (marked by a red asterisk).
(PDF)

Figure S7 Maximum likelihood phylogeny of major cyclophilins from *T. inflatum*, *H. sapiens*, *C. elegans*, *D. melanogaster*, and other characterized cyclophilins from other fungi, bacteria, and protists. Phylogeny was constructed from an alignment of the conserved cyclophilin-like domain (CLD) using RAxML with the best fit model identified by ProtTest (WAG+G) model and 100 bootstrap replicates. The phylogenetic positions of all *T. inflatum* cyclophilins (shaded light blue), the human cyclophilin A (hCypA), the yeast CypA homologs Cpr1 and Cpr2, the *simA* cluster cyclophilins (TINF00586), and TINF04375 are shown.
(PDF)

Figure S8 Maximum likelihood phylogenies of top 25 BLAST hits to each gene in the *simA* cluster plus 10 genes flanking the antiSMASH predicted cluster from the 5′ to 3′ end. Most *T. inflatum* genes (branches shown in blue) that are located outside of the RNA-Seq defined metabolite cluster have single copy orthologs in other hypocrealean taxa, while those inside the cluster mostly lack orthologs in other hypocrealean taxa.
(PDF)

Table S1 A) Numbers of major classes of fungal repeat elements in the *T. inflatum* genome characterized by RepeatMasker. The *T. inflatum* genome contains a large number of DNA hAT transposons. B) Number of *CPA* element and *Restles*s transposons found in other hypocrealean taxa.
(DOCX)

Table S2 Results from CAFÉ analyses of gene family expansions and contractions of CAZys, P450s, and proteases across the fourteen hypocrealean taxa and nodes in the phylogeny (Figure 3) are shown across the top and color coded according to ecology or predicted ancestral ecology (red = animal associated, blue = fungal associated, green = plant associated). Numbers of genes found in each taxa are listed in each cell and taxa or nodes that have significant expansions (E) or contractions (C) at p<0.01are shaded with gray or hatched lines respectively.
(DOCX)

Table S3 Table of identified *T. inflatum* core secondary metabolites including NRPSs, PKSs, NRPS-like, PKS-like, and DMAT enzymes.
(DOCX)

Table S4 NRPSs in *T. inflatum* and other hypocrealean taxa with A-domains that group with known NRPSs (top row) in larger phylogeny (Figure S3). The number of modules (M) present in each NRPS is listed after each gene.
(DOCX)

Table S5 Table of q-values, fold change, and RPKM values at six time points (days 2, 4, 6, 8, 10, 12) in the cyclosporin time course experiment. Genes in the RNA-Seq defined cluster are shaded green.
(DOCX)

Table S6 Table of q-values, fold change, and RPKM values at six time points (days 2, 4, 6, 8, 10, 12) in analyses of SDB, cuticle, and hemolymph media. Genes in the RNA-Seq defined cluster are shaded green.
(DOCX)

## References

[1] Borel JF (1997) Cyclosporin in immunology: Past, present and future. Biodrugs 8: 1–3.

[2] Wang P, Heitman J (2005) The cyclophilins. Genome Biology 6: 226. 15998457 10.1186/gb-2005-6-7-226 PMC1175980

[3] Handschumacher RE, Harding MW, Rice J, Drugge RJ (1984) Cyclophilin - A specific cytosolic binding-protein for Cyclosporin-A. Science 226: 544–547. 6238408 10.1126/science.6238408

[4] Liu J, Farmer JD, Lane WS, Friedman J, Weissman I, et al (1991) Calcineurin is a common target of cyclophilin-Cyclosporine-A and FKBP-FK506 complexes. Cell 66: 807–815. 1715244 10.1016/0092-8674(91)90124-h

[5] Jorgensen KA, Koefoed-Nielsen PB, Karamperis N (2003) Calcineurin phosphatase activity and immunosuppression. A review on the role of calcineurin phosphatase activity and the immunosuppressive effect of cyclosporin A and tacrolimus. Scandinavian Journal of Immunology 57: 93–98. 12588654 10.1046/j.1365-3083.2003.01221.x

[6] Okeefe SJ, Tamura J, Kincaid RL, Tocci MJ, Oneill EA (1992) FK-506-sensitive and CsA-sensitive activation of the Interleukin-2 promotor by calcineurin. Nature 357: 692–694. 1377361 10.1038/357692a0

[7] Cruz MC, Del Poeta M, Wang P, Wenger R, Zenke G, et al (2000) Immunosuppressive and nonimmunosuppressive cyclosporine analogs are toxic to the opportunistic fungal pathogen Cryptococcus neoformans via cyclophilin-dependent inhibition of calcineurin. Antimicrobial Agents and Chemotherapy 44: 143–149. 10602736 10.1128/aac.44.1.143-149.2000 PMC89641

[8] Nakagawa M, Sakamoto N, Enomoto N, Tanabe Y, Kanazawa N, et al (2004) Specific inhibition of hepatitis C virus replication by cyclosporin A. Biochemical and Biophysical Research Communications 313: 42–47. 14672695 10.1016/j.bbrc.2003.11.080

[9] Marahiel MA, Stachelhaus T, Mootz HD (1997) Modular peptide synthetases involved in nonribosomal peptide synthesis. Chemical Reviews 97: 2651–2673. 11851476 10.1021/cr960029e

[10] Weber G, Schorgendorfer K, Schneiderscherzer E, Leitner E (1994) The peptide synthetase catalyzing cyclosporine production in *Tolypocladium-niveum* is encoded by a giant 45.8-kilobase open reading frame. Current Genetics 26: 120–125. 8001164 10.1007/BF00313798

[11] Isaka M, Kittakoop P (2003) Secondary Metabolites of Clavicipitalean Fungi. In: White JF, Bacon CW, Hywel-Jones NL, Spatafora JW, editors. Clavicipitalean Fungi: Evolutionary Biology, Chemistry, Biocontrol, and Cultural Impacts. New York, NY: Marcel Dekker Inc. pp 355–397.

[12] Cane DE, Walsh CT (1999) The parallel and convergent universes of polyketide synthases and nonribosomal peptide synthetases. Chemistry & Biology 6: R319–R325. 10631508 10.1016/s1074-5521(00)80001-0

[13] Pal S, Leger RJS, Wu LP (2007) Fungal peptide destruxin a plays a specific role in suppressing the innate immune response in *Drosophila melanogaster* . Journal of Biological Chemistry 282: 8969–8977. 17227774 10.1074/jbc.M605927200

[14] Bandani AR, Khambay BPS, Faull JL, Newton R, Deadman M, et al (2000) Production of efrapeptins by *Tolypocladium* species and evaluation of their insecticidal and antimicrobial properties. Mycological Research 104: 537–544.

[15] Torres MS, Singh AP, Vorsa N, White JF Jr (2008) An analysis of ergot alkaloids in the Clavicipitaceae (Hypocreales, Ascomycota) and ecological implications. Symbiosis 46: 11–19.

[16] Sung GH, Poinar GO, Spatafora JW (2008) The oldest fossil evidence of animal parasitism by fungi supports a Cretaceous diversification of fungal-arthropod symbioses. Molecular Phylogenetics and Evolution 49: 495–502. 18817884 10.1016/j.ympev.2008.08.028

[17] Spatafora JW, Sung GH, Sung JM, Hywel-Jones NL, White JF (2007) Phylogenetic evidence for an animal pathogen origin of ergot and the grass endophytes. Molecular Ecology 16: 1701–1711. 17402984 10.1111/j.1365-294X.2007.03225.x

[18] Hodge K, Krasnoff S, Humber RA (1996) *Tolypocladium inflatum* is the anamorph of *Cordyceps subsessilis* . Mycologia 88: 715–719.

[19] Gao QA, Jin K, Ying SH, Zhang YJ, Xiao GH, et al (2011) Genome sequencing and comparative transcriptomics of the model entomopathogenic fungi *Metarhizium anisopliae* and *M. acridum* . PLoS Genetics 7: e1001264. 21253567 10.1371/journal.pgen.1001264 PMC3017113

[20] Bidochka MJ, St Leger RJ, Stuart A, Gowanlock K (1999) Nuclear rDNA phylogeny in the fungal genus *Verticillium* and its relationship to insect and plant virulence, extracellular proteases and carbohydrases. Microbiology-UK 145: 955–963. 10.1099/13500872-145-4-955 10220175

[21] Zheng Peng, Xia Yongliang, Xiao Guohua, Chenghui Xiong, Hu Xiao, et al (2011) Genome sequence of the insect pathogenic fungus *Cordyceps militaris*, a valued traditional Chinese medicine. Genome Biology 12: R116. 22112802 10.1186/gb-2011-12-11-r116 PMC3334602

[22] Stimberg N, Walz M, Schorgendorfer K, Kuck U (1992) Electrophoretic karyotyping from *Tolypocladium-inflatum* and 6 related strains allows differentiation of morphologically similar species. Applied Microbiology and Biotechnology 37: 485–489.

[23] Holt C, Yandell M (2011) MAKER2: an annotation pipeline and genome-database management tool for second-generation genome projects. BMC Bioinformatics 12: 491. 22192575 10.1186/1471-2105-12-491 PMC3280279

[24] Parra G, Bradnam K, Korf I (2007) CEGMA: a pipeline to accurately annotate core genes in eukaryotic genomes. Bioinformatics 23: 1061–1067. 17332020 10.1093/bioinformatics/btm071

[25] Stajich JE, Deietrich FS, Roy SW (2007) Comparative genomic analysis of fungal genomes reveals intron-rich ancestors. Genome Biology 8: R223. 17949488 10.1186/gb-2007-8-10-r223 PMC2246297

[26] Kempken F, Schreiner C, Schorgendorfer K, Kuch U (1995) A unique repeated DNA sequence in the cyclosporin-producing strain of *Tolypocladium inflatum* (ATCC 34921). Experimental Mycology 19: 305–313. 8574905 10.1006/emyc.1995.1037

[27] Kempken F, Kuck U (1996) restless, an active Ac-like transposon from the fungus *Tolypocladium inflatum*: Structure, expression, and alternative RNA splicing. Molecular and Cellular Biology 16: 6563–6572. 8887685 10.1128/mcb.16.11.6563 PMC231658

[28] Kempken F (2008) The *Tolypocladium inflatum* CPA element encodes a RecQ helicase-like gene. Journal of Basic Microbiology 48: 496–499. 18792048 10.1002/jobm.200800164

[29] Windhofer F, Hauck K, Catcheside DEA, Kuck U, Kempken F (2002) Ds-like *Restless* deletion derivatives occur in *Tolypocladium inflatum* and two foreign hosts, *Neurospora crassa* and *Penicillium chrysogenum* . Fungal Genetics and Biology 35: 171–182. 11848679 10.1006/fgbi.2001.1323

[30] Van Dongen S (2008) Graph clustering via a discrete uncoupling process. Siam Journal on Matrix Analysis and Applications 30: 121–141.

[31] Robbertse B, Yoder R, Boyd A, Reeves J, Spatafora J (2011) Hal: an automated pipeline for phylogenetic analyses of genomic data. PLoS Curr 3: RRN1213. 21327165 10.1371/currents.RRN1213 PMC3038436

[32] Kubicek CP, Herrera-Estrella A, Seidl-Seiboth V, Martinez DA, Druzhinina IS, et al (2011) Comparative genome sequence analysis underscores mycoparasitism as the ancestral life style of *Trichoderma* . Genome Biology 12: R40. 21501500 10.1186/gb-2011-12-4-r40 PMC3218866

[33] Rodriguez-Ezpeleta N, Brinkmann H, Roure B, Lartillot N, Lang BF, et al (2007) Detecting and overcoming systematic errors in genome-scale phylogenies. Systematic Biology 56: 389–399. 17520503 10.1080/10635150701397643

[34] Xiao G, Ying S-H, Zheng P, Wang Z-L, Zhang S, et al (2012) Genomic perspectives on the evolution of fungal entomopathogenicity in *Beauveria bassiana* . Scientific Reports 2: 483. 22761991 10.1038/srep00483 PMC3387728

[35] Pava-Ripoll M, Angelini C, Fang WG, Wang SB, Posada FJ, et al (2011) The rhizosphere-competent entomopathogen *Metarhizium anisopliae* expresses a specific subset of genes in plant root exudate. Microbiology-SGM 157: 47–55. 10.1099/mic.0.042200-0 20947574

[36] Dreyfuss M, Harri E, Hofmann H, Kobel H, Pache W, et al (1976) Cyclosporin-A and C new metabolites from *Trichoderma-polysporum* (Link Ex Pers) rifai. European Journal of Applied Microbiology 3: 125–133.

[37] Krasnoff SB, Gupta S (1992) Efrapeptin production by *Tolypocladium* fungi (Deuteromycotina, Hyphomycetes) - Intraspecific and interspecific Variation. Journal of Chemical Ecology 18: 1727–1741. 24254715 10.1007/BF02751098

[38] Weiser J, Matha V (1988) Tolypin, a new insecticidal metabolite of fungi of the genus *Tolypocladium* . Journal of Invertebrate Pathology 51: 94–96. 3351326 10.1016/0022-2011(88)90093-6

[39] Chu M, Mierzwa R, Truumees I, Gentile F, Patel M, et al (1993) 2 novel diketopiperazines isolated from the fungus *Tolypocladium* sp. Tetrahedron Letters 34: 7537–7540.

[40] Keller NP, Hohn TM (1997) Metabolic pathway gene clusters in filamentous fungi. Fungal Genetics and Biology 21: 17–29. 9126615

[41] Walton JD (2000) Horizontal gene transfer and the evolution of secondary metabolite gene clusters in fungi: An hypothesis. Fungal Genetics and Biology 30: 167–171. 11035938 10.1006/fgbi.2000.1224

[42] Gacek A, Strauss J (2012) The chromatin code of fungal secondary metabolite gene clusters. Applied Microbiology and Biotechnology 95: 1389–1404. 22814413 10.1007/s00253-012-4208-8 PMC3427479

[43] Khaldi N, Seifuddin FT, Turner G, Haft D, Nierman WC, et al (2010) SMURF: Genomic mapping of fungal secondary metabolite clusters. Fungal Genetics and Biology 47: 736–741. 20554054 10.1016/j.fgb.2010.06.003 PMC2916752

[44] Medema MH, Blin K, Cimermancic P, de Jager V, Zakrzewski P, et al (2011) antiSMASH: rapid identification, annotation and analysis of secondary metabolite biosynthesis gene clusters in bacterial and fungal genome sequences. Nucleic Acids Research 39: W339–W346. 21672958 10.1093/nar/gkr466 PMC3125804

[45] Panaccione DG (2005) Origins and significance of ergot alkaloid diversity in fungi. FEMS Microbiology Letters 251: 9–17. 16112823 10.1016/j.femsle.2005.07.039

[46] Tudzynski P, Hoelter K, Correia T, Arntz C, Grammel N, et al (1999) Evidence for an ergot alkaloid gene cluster in *Claviceps purpurea* . Molecular and General Genetics 261: 133–141. 10071219 10.1007/s004380050950

[47] Hoffmeister D, Keller NP (2007) Natural products of filamentous fungi: enzymes, genes, and their regulation. Natural Product Reports 24: 393–416. 17390002 10.1039/b603084j

[48] Vonwartburg A, Traber R (1986) Chemistry of the natural cyclosporine metabolites. Progress in Allergy 38: 28–45. 3725752

[49] Traber R, Dreyfuss MM (1996) Occurrence of cyclosporins and cyclosporin-like peptolides in fungi. Journal of Industrial Microbiology & Biotechnology 17: 397–401.

[50] Nakajima H, Hamasaki T, Tanaka K, Kimura Y, Udagawa S, et al (1989) Production of cyclosporin by fungi belonging to the genus *Neocosmospora* . Agric Biol Chem 53: 2291–2292.

[51] Sallam LAR, El-Refai AMH, Hamdy AHA, El-Minofi HA, Abdel-Salam IS (2003) Role of some fermentation parameters on cyclosporin A production by a new isolate of *Aspergillus terreus* . Journal of General and Applied Microbiology 49: 321–328. 14747973 10.2323/jgam.49.321

[52] Sakamoto K, Tsujii E, Miyauchi M, Nakanishi T, Yamashita M, et al (1993) FR901459, a novel immunosuppressant isolated from *Stachybotrys chartarum* No. 19392. J Antibiotics 46: 1788–1798. 8294235 10.7164/antibiotics.46.1788

[53] Dreyfuss MM (1986) Neue Erkenntnisse aus einem pharmakologischen Screening. Sydowia 39: 22–36.

[54] Bushley KE, Turgeon BG (2010) Phylogenomics reveals subfamilies of fungal nonribosomal peptide synthetases and their evolutionary relationships. BMC Evolutionary Biology 10: 26. 20100353 10.1186/1471-2148-10-26 PMC2823734

[55] Haese A, Schubert M, Herrmann M, Zocher R (1993) Molecular characterization of the Enniatin synthetase gene encoding a multifunctional enzyme catalyzing N-methyldepsipeptide formation in *Fusarium-scirpi* . Molecular Microbiology 7: 905–914. 8483420 10.1111/j.1365-2958.1993.tb01181.x

[56] Xu YQ, Orozco R, Wijeratne EMK, Gunatilaka AAL, Stock SP, et al (2008) Biosynthesis of the cyclooligomer depsipeptide Beauvericin, a virulence factor of the entomopathogenic fungus *Beauveria bassiana* . Chemistry & Biology 15: 898–907. 18804027 10.1016/j.chembiol.2008.07.011

[57] Xu YQ, Rozco R, Wijeratne EMK, Espinosa-Artiles P, Gunatilaka AAL, et al (2009) Biosynthesis of the cyclooligomer depsipeptide bassianolide, an insecticidal virulence factor of *Beauveria bassiana* . Fungal Genetics and Biology 46: 353–364. 19285149 10.1016/j.fgb.2009.03.001

[58] Fiolka M (2008) Immunosuppressive effect of cyclosporin A on insect humoral immune response. Journal of Invertebrate Pathology 98: 287–292. 18472108 10.1016/j.jip.2008.03.015

[59] Endo M, Takesako K, Kato I, Yamaguchi H (1997) Fungicidal action of aureobasidin A, a cyclic depsipeptide antifungal antibiotic, against *Saccharomyces cerevisiae* . Antimicrobial Agents and Chemotherapy 41: 672–676. 9056012 10.1128/aac.41.3.672 PMC163770

[60] Cardenas ME, Cruz MC, Del Poeta M, Chung NJ, Perfect JR, et al (1999) Antifungal activities of antineoplastic agents *Saccharomyces cerevisiae* as a model system to study drug action. Clinical Microbiology Reviews 12: 583–611. 10515904 10.1128/cmr.12.4.583 PMC88926

[61] Bushley KE, Ripoll DR, Turgeon BG (2008) Module evolution and substrate specificity of fungal nonribosomal peptide synthetases involved in siderophore biosynthesis. BMC Evolutionary Biology 8: 328. 19055762 10.1186/1471-2148-8-328 PMC2644324

[62] Pieper R, Kleinkauf H, Zocher R (1992) Enniatin synthetases from different Fusaria exhibiting distinct amino-acid specificities. Journal of Antibiotics 45: 1273–1277. 1399848 10.7164/antibiotics.45.1273

[63] Slightom JL, Metzger BR, Luu HT, Elhammer AP (2009) Cloning and molecular characterization of the gene encoding the Aureobasidin A biosynthesis complex in *Aureobasidium pullulans* BP-1938. Gene 431: 67–79. 19084058 10.1016/j.gene.2008.11.011

[64] Pond SLK, Murrell B, Fourment M, Frost SDW, Delport W, et al (2011) A Random Effects Branch-Site Model for Detecting Episodic Diversifying Selection. Molecular Biology and Evolution 28: 3033–3043. 21670087 10.1093/molbev/msr125 PMC3247808

[65] Lee MJ, Lee HN, Han K, Kim ES (2008) Spore inoculum optimization to maximize cyclosporin a production in *Tolypocladium niveum* . Journal of Microbiology and Biotechnology 18: 913–917. 18633291

[66] Samel SA, Marahiel MA, Essen LO (2008) How to tailor non-ribosomal peptide products - new clues about the structures and mechanisms of modifying enzymes. Molecular Biosystems 4: 387–393. 18414736 10.1039/b717538h

[67] Hoffmann K, Schneiderscherzer E, Kleinkauf H, Zocher R (1994) Purification and characterization of eukaryotic alanine racemase acting as key enzyme in cyclosporine biosynthesis. Journal of Biological Chemistry 269: 12710–12714. 8175682

[68] Offenzeller M, Santer G, Totschnig K, Su Z, Moser H, et al (1996) Biosynthesis of the unusual amino acid (4R)-4-[(E)-2-butenyl]-4-methyl-L-threonine of cyclosporin A: Enzymatic analysis of the reaction sequence including identification of the methylation precursor in a polyketide pathway. Biochemistry 35: 8401–8412. 8679598 10.1021/bi960224n

[69] Sanglier JJ, Traber R, Buck RH, Hofmann H, Kobel H (1990) Isolation of (4-R)-R-(E)-2-Butenyl-4-Methyl-L-Threonine, the characteristic structural element of cyclosporins, rrom a blocked mutant of *Tolypocladium-inflatum* . Journal of Antibiotics 43: 707–714. 2116402 10.7164/antibiotics.43.707

[70] Thériault Yves, Logan Timothy M, Meadows Robert, Yu Liping, Olejniczak Edward T, et al (1993) Solution structure of the cyclosporin A/cyclophilin complex by NMR. Nature 361: 88–90. 8421500 10.1038/361088a0

[71] Pflügl Gaston, Kallen Jörg, Schirmer Tilman, Jansonius Johan N, Mauro GM, et al (1993) X-ray structure of a decameric cyclophilin-cyclosporin crystal complex. Nature 361: 91–93. 8421501 10.1038/361091a0

[72] Galat A (1999) Variations of sequences and amino acid compositions of proteins that sustain their biological functions: An analysis of the cyclophilin family of proteins. Archives of Biochemistry and Biophysics 371: 149–162. 10545201 10.1006/abbi.1999.1434

[73] Galat A (2003) Peptidylprolyl cis/trans isomerases (immunophilins): Biological diversity targets - Functions. Current Topics in Medicinal Chemistry 3: 1315–1347. 12871165 10.2174/1568026033451862

[74] Gothel SF, Marahiel MA (1999) Peptidyl-prolyl cis-trans isomerases, a superfamily of ubiquitous folding catalysts. Cellular and Molecular Life Sciences 55: 423–436. 10228556 10.1007/s000180050299 PMC11146858

[75] Chen MM, Jiang MG, Shang JJ, Lan XW, Yang F, et al (2011) CYP1, a hypovirus-regulated cyclophilin, is required for virulence in the chestnut blight fungus. Molecular Plant Pathology 12: 239–246. 21355996 10.1111/j.1364-3703.2010.00665.x PMC3313458

[76] Hermans PWM, Adrian PV, Albert C, Estevao S, Hoogenboezem T, et al (2006) The streptococcal lipoprotein rotamase A (SlrA) is a functional peptidyl-prolyl isomerase involved in pneumococcal colonization. Journal of Biological Chemistry 281: 968–976. 16260779 10.1074/jbc.M510014200

[77] Emanuelsson O, Nielsen H, Brunak S, von Heijne G (2000) Predicting subcellular localization of proteins based on their N-terminal amino acid sequence. Journal of Molecular Biology 300: 1005–1016. 10891285 10.1006/jmbi.2000.3903

[78] Viaud M, Brunet-Simon A, Brygoo Y, Pradier JM, Levis C (2003) Cyclophilin A and calcineurin functions investigated by gene inactivation, cyclosporin A inhibition and cDNA arrays approaches in the phytopathogenic fungus *Botrytis cinerea* . Molecular Microbiology 50: 1451–1465. 14651630 10.1046/j.1365-2958.2003.03798.x

[79] Viaud MC, Balhadere PV, Talbot NJ (2002) A *Magnaporthe grisea* cyclophilin acts as a virulence determinant during plant infection. Plant Cell 14: 917–930. 11971145 10.1105/tpc.010389 PMC150692

[80] Wang P, Cardenas ME, Cox CM, Perfect JR, Heitman J (2001) Two cyclophilin A homologs with shared and distinct functions important for growth and virulence of *Cryptococcus neoformans* . Embo Reports 2: 511–518. 11415984 10.1093/embo-reports/kve109 PMC1083903

[81] Hornbogen T, Zocher R (1995) Cloning and sequencing of a cyclophilin gene from the cyclosporine producer *Tolypocladium-niveum* . Biochemistry and Molecular Biology International 36: 169–176. 7663412

[82] Weber G, Leitner E (1994) Disruption of the cyclosporine synthetase gene of *Tolypocladium-niveum* . Current Genetics 26: 461–467. 7874740 10.1007/BF00309935

[83] Tropschug M, Nicholson DW, Hartl FU, Kohler H, Pfanner N, et al (1988) Cyclosporin A-binding protein (Cyclophilin) of *Neurospora crassa* - One gene codes for both the cytosolic and mitochondrial Forms. Journal of Biological Chemistry 263: 14433–14440. 2971658

[84] Dumas C, Ravallec M, Matha V, Vey A (1996) Comparative study of the cytological aspects of the mode of action of destruxins and other peptidic fungal metabolites on target epithelial cells. Journal of Invertebrate Pathology 67: 137–146.

[85] Hornbogen T, Pieper R, Hoffmann K, Kleinkauf H, Zocher R (1992) 2 New cyclophilins from *Fusarium sambucinum* and *Aspergillus-niger* - Resistance of cyclophilin Cyclosporine A complexes against proteolysis. Biochemical and Biophysical Research Communications 187: 791–796. 1530635 10.1016/0006-291x(92)91265-r

[86] Vilcinskas A, Kopacek P, Jegorov A, Vey A, Matha V (1997) Detection of lipophorin as the major cyclosporin-binding protein in the hemolymph of the greater wax moth *Galleria mellonella* . Comparative Biochemistry and Physiology C-Pharmacology Toxicology & Endocrinology 117: 41–45.

[87] Lee BN, Kroken S, Chou DYT, Robbertse B, Yoder OC, et al (2005) Functional analysis of all nonribosomal peptide synthetases in *Cochliobolus heterostrophus* reveals a factor, NPS6, involved in virulence and resistance to oxidative stress. Eukaryotic Cell 4: 545–555. 15755917 10.1128/EC.4.3.545-555.2005 PMC1087798

[88] Fu J, Wenzel SC, Perlova O, Wang JP, Gross F, et al (2008) Efficient transfer of two large secondary metabolite pathway gene clusters into heterologous hosts by transposition. Nucleic Acids Research 36: e113. 18701643 10.1093/nar/gkn499 PMC2553598

[89] Slot JC, Rokas A (2011) Horizontal transfer of a large and highly toxic secondary metabolic gene cluster between fungi. Current Biology 21: 134–139. 21194949 10.1016/j.cub.2010.12.020

[90] Khaldi N, Collemare J, Lebrun MH, Wolfe KH (2008) Evidence for horizontal transfer of a secondary metabolite gene cluster between fungi. Genome Biology 9: R18. 18218086 10.1186/gb-2008-9-1-r18 PMC2395248

[91] Gardiner DM, Howlett BJ (2005) Bioinformatic and expression analysis of the putative gliotoxin biosynthetic gene cluster of *Aspergillus fumigatus* . FEMS Microbiology Letters 248: 241–248. 15979823 10.1016/j.femsle.2005.05.046

[92] Anaya N, Roncero MIG (1995) skippy, a retrotransposon from the fungal plant pathogen *Fusarium oxysporum* . Molecular & General Genetics 249: 637–647. 8544829 10.1007/BF00418033

[93] Lea Ruiqiang (2008) SOAP: short oligonucleotide alignment program. Bioinformatics 24: 713–714. 18227114 10.1093/bioinformatics/btn025

[94] Schulz MH, Zerbino DR, Vingron M, Birney E (2012) Oases: robust de novo RNA-seq assembly across the dynamic range of expression levels. Bioinformatics 28: 1086–1092. 22368243 10.1093/bioinformatics/bts094 PMC3324515

[95] Lowe TM, Eddy SR (1997) tRNAscan-SE: A program for improved detection of transfer RNA genes in genomic sequence. Nucleic Acids Research 25: 955–964. 9023104 10.1093/nar/25.5.955 PMC146525

[96] Edgar RC (2004) MUSCLE: multiple sequence alignment with high accuracy and high throughput. Nucleic Acids Research 32: 1792–1797. 15034147 10.1093/nar/gkh340 PMC390337

[97] Abascal F, Zardoya R, Posada D (2005) ProtTest: Selection of best-fit models of protein evolution. Bioinformatics 21: 2104–2105. 15647292 10.1093/bioinformatics/bti263

[98] Yang ZH (2007) PAML 4: Phylogenetic analysis by maximum likelihood. Molecular Biology and Evolution 24: 1586–1591. 17483113 10.1093/molbev/msm088

[99] Sanderson MJ (2003) r8s: inferring absolute rates of molecular evolution and divergence times in the absence of a molecular clock. Bioinformatics 19: 301–302. 12538260 10.1093/bioinformatics/19.2.301

[100] O'Brien KP, Remm M, Sonnhammer ELL (2005) Inparanoid: a comprehensive database of eukaryotic orthologs. Nucleic Acids Research 33: D476–D480. 15608241 10.1093/nar/gki107 PMC540061

[101] Berglund AC, Sjolund E, Ostlund G, Sonnhammer ELL (2008) InParanoid 6: eukaryotic ortholog clusters with inparalogs. Nucleic Acids Research 36: D263–D266. 18055500 10.1093/nar/gkm1020 PMC2238924

[102] Emanuelsson O, Brunak S, von Heijne G, Nielsen H (2007) Locating proteins in the cell using TargetP, SignalP and related tools. Nature Protocols 2: 953–971. 17446895 10.1038/nprot.2007.131

[103] Small I, Peeters N, Legeai F, Lurin C (2004) Predotar: A tool for rapidly screening proteomes for N-terminal targeting sequences. Proteomics 4: 1581–1590. 15174128 10.1002/pmic.200300776

[104] Krogh A, Larsson B, von Heijne G, Sonnhammer ELL (2001) Predicting transmembrane protein topology with a hidden Markov model: Application to complete genomes. Journal of Molecular Biology 305: 567–580. 11152613 10.1006/jmbi.2000.4315

[105] Rawlings ND, Barrett AJ, Bateman A (2012) MEROPS: the database of proteolytic enzymes, their substrates and inhibitors. Nucleic Acids Research 40: D343–D350. 22086950 10.1093/nar/gkr987 PMC3245014

[106] Nelson DR (2002) Mining databases for cytochrome P450 genes. Methods in Enzymology: Cytochrome P450, Part C 357: 3–15. 10.1016/s0076-6879(02)57660-6 12424892

[107] Katoh M, Kuma Miyata (2002) MAFFT: a novel method for rapid multiple sequence alignment based on fast fourier transform. Nucleic Acids Res 30: 3059–3066. 12136088 10.1093/nar/gkf436 PMC135756

[108] Stamatakis A, Hoover P, Rougemont J (2008) A rapid bootstrap algorithm for the RAxML web-servers. Systematic Biology 75: 758–771. 10.1080/10635150802429642 18853362

[109] Darling AE, Mau B, Perna NT (2010) progressiveMauve: Multiple genome alignment with gene gain, loss and rearrangement. Plos One 5: e11147. 20593022 10.1371/journal.pone.0011147 PMC2892488

[110] St Leger RJ, Nelson JO, Screen SE (1999) The entomopathogenic fungus *Metarhizium anisopliae* alters ambient pH, allowing extracellular protease production and activity. Microbiology-UK 145: 2691–2699. 10.1099/00221287-145-10-2691 10537191

[111] St Leger RJ, Bidochka MJ, Roberts DW (1994) Isoforms of the cuticle degrading Pr1 protease and production of a metalloproteinase by *Metarhizium anisopliae* . Arch Biochem Biophysical Journal 313: 1–7. 10.1006/abbi.1994.1350 8053668

[112] St. Leger RJ, Bidochka MJ, Roberts DW (1994) Characterization of a novel carboxypeptidase produced by the entomopathogenic fungus *Metarhizium anisopliae* . Arch Biochem Biophys 314: 392–398. 7979380 10.1006/abbi.1994.1458

[113] Cumbie JS, Kimbrel JA, Di YM, Schafer DW, Wilhelm LJ, et al (2011) GENE-Counter: A Computational Pipeline for the Analysis of RNA-Seq Data for Gene Expression Differences. PloS ONE 6: e25279. 21998647 10.1371/journal.pone.0025279 PMC3188579

[114] Di YM, Schafer DW, Cumbie JS, Chang JH (2011) The NBP Negative Binomial Model for Assessing Differential Gene Expression from RNA-Seq. Statistical Applications in Genetics and Molecular Biology 10 doi: 10.2202/1544-6115.1637

[115] Mortazavi A, Williams BA, McCue K, Schaeffer L, Wold B (2008) Mapping and quantifying mammalian transcriptomes by RNA-Seq. Nature Methods 5: 621–628. 18516045 10.1038/nmeth.1226 PMC13303166

