# Supplementary figures and images for: The Genome of Tolypocladium inflatum: Evolution, Organization, and Expression of the Cyclosporin Biosynthetic Gene Cluster
Source: PLoS Genet. 2013 Jun 20;9(6):e1003496. doi: 10.1371/journal.pgen.1003496 (PMC3688495; doi:10.1371/journal.pgen.1003496)

***Tolypocladium inflatum* NRRL 8044 - MAT1-2 locus**

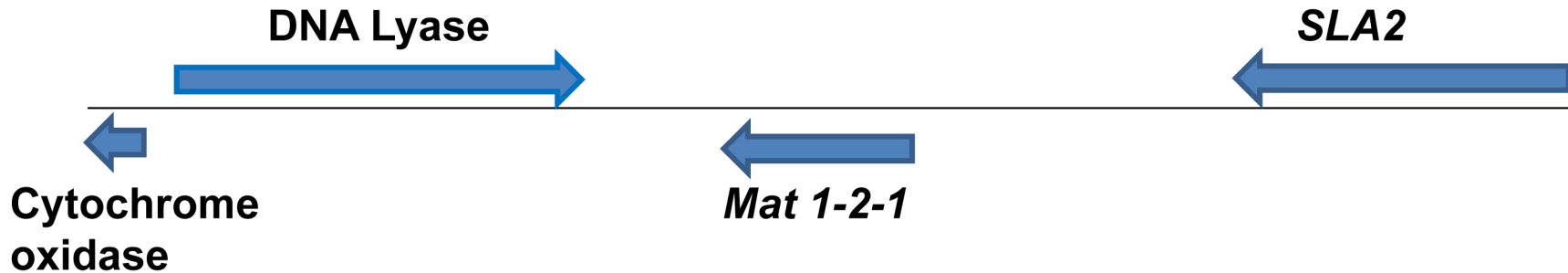

Supplement: Figure S1 — The MAT1-2 Mating Locus of T. inflatum NRRL 8044. Only a single mating type was found in this strain, indicating that the fungus is likely heterothallic. (PDF) [file pgen.1003496.s001.pdf]

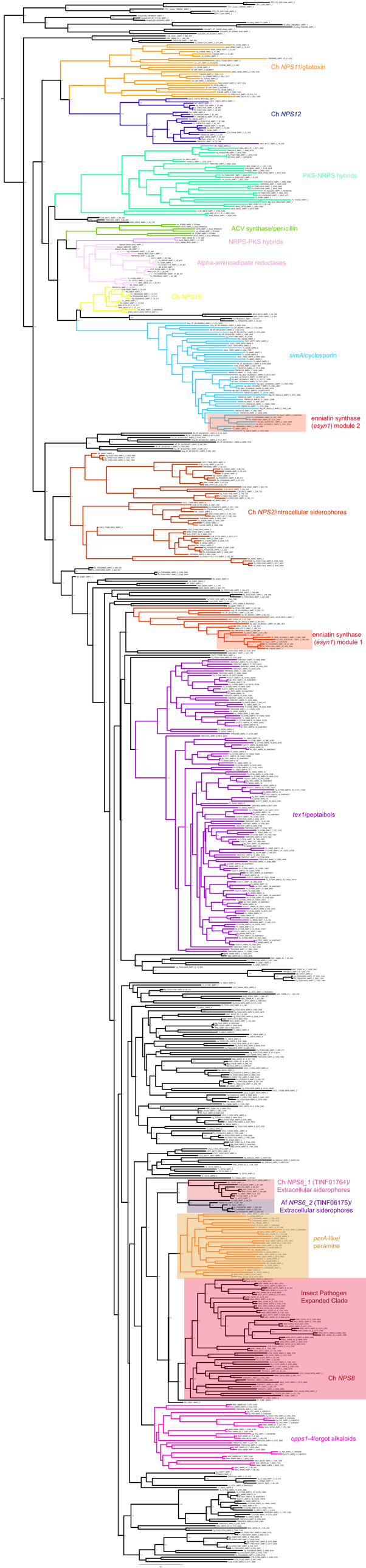

Supplement: Figure S3 — Maximum likelihood phylogeny of 696 NRPS A-domains from 14 hypocrealean taxa showing previously characterized groups of NRPSs or those with known chemical products: Ch NPS11/gliotoxin (dark orange), Ch NPS12 (dark blue), PKS-NRPS hybrids (blue green), ACV synthases (yellow green), NRPS-PKS hybrids (lavender), alpha-aminoadipate reductases (light pink), Ch NPS10 (yellow), simA/cyclosporin clade (turquoise), enniatin synthase (esyn1) module 2 (red), Ch NPS2 intracellular siderophore synthases (brown), enniatin synthase (esyn1) module 1 (red), tex1/peptaibols (dark purple), duplicated paralogous copies of Ch NPS6 (Ch NPS6_1- pink and Ch NPS6_2 - purple), perA-like/peramine (light orange), Ch NPS8/insect expanded clade (brick), and cpps1-4/ergot alkaloids (bright pink). Phylogeny constructed by maximum likelihood in RAxML using the PROTGAMMARTREV model and 1000 bootstrap replicates. (PDF) [file pgen.1003496.s003.pdf]

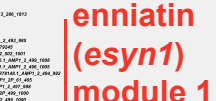

# enniatin (esyn1) module 1

Supplement: Figure S5 — Phylogeny of A-domains from top 50 BLAST hits to the simA NRPS in the NCBI nr database. Although the simA clade (turquoise) groups near a large clade of bacterial NRPSs (orange), no bacterial NRPSs were found within the simA clade. Phylogeny constructed by maximum likelihood in RAxML using the PROTGAMMARTREV model and 100 bootstrap replicates. (PDF) [file pgen.1003496.s005.pdf]

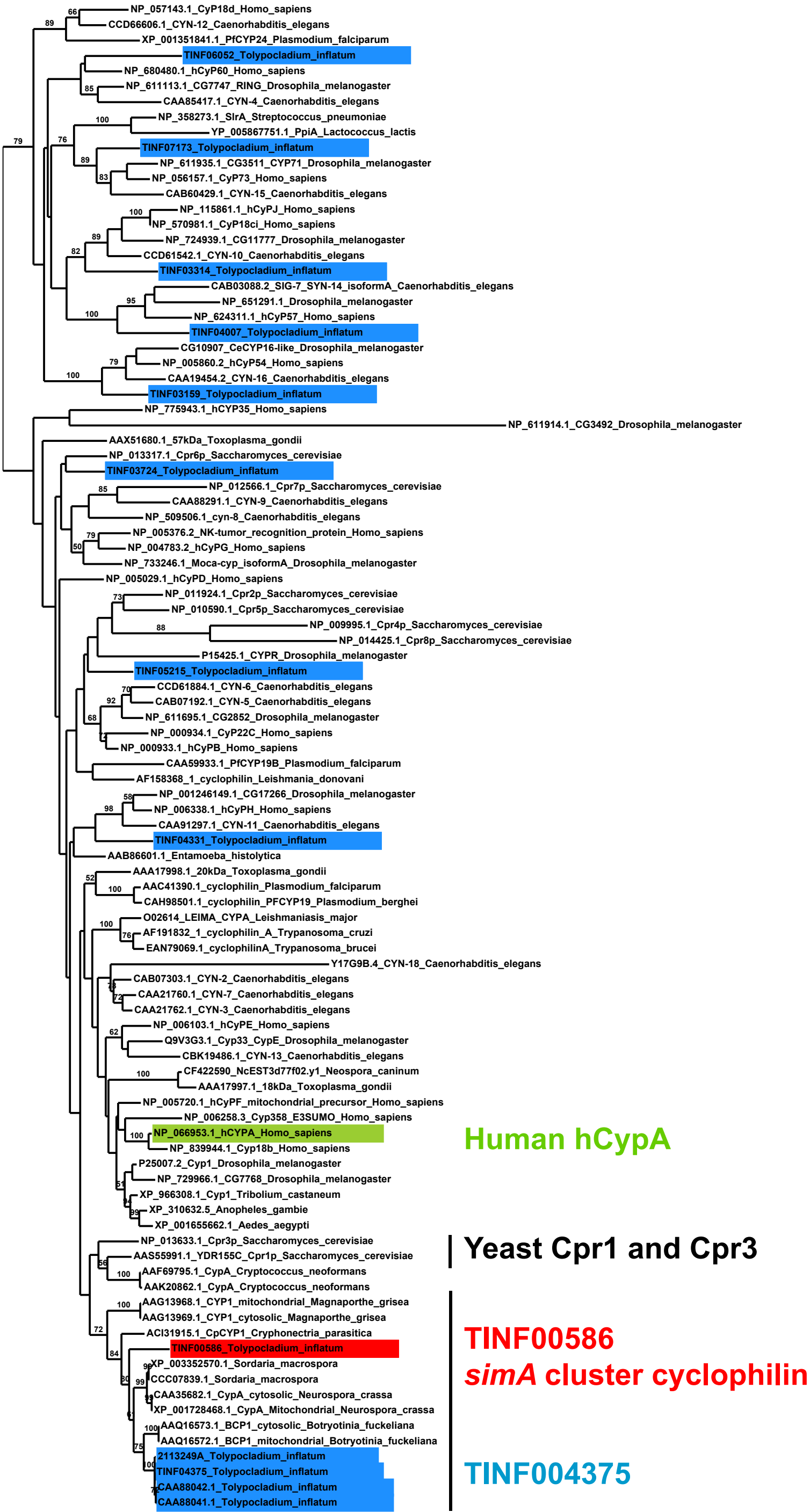

Supplement: Figure S7 — Maximum likelihood phylogeny of major cyclophilins from T. inflatum, H. sapiens, C. elegans, D. melanogaster, and other characterized cyclophilins from other fungi, bacteria, and protists. Phylogeny was constructed from an alignment of the conserved cyclophilin-like domain (CLD) using RAxML with the best fit model identified by ProtTest (WAG+G) model and 100 bootstrap replicates. The phylogenetic positions of all T. inflatum cyclophilins (shaded light blue), the human cyclophilin A (hCypA), the yeast CypA homologs Cpr1 and Cpr2, the simA cluster cyclophilins (TINF00586), and TINF04375 are shown. (PDF) [file pgen.1003496.s007.pdf]
