# Supplementary material for: The Genome of Tolypocladium inflatum: Evolution, Organization, and Expression of the Cyclosporin Biosynthetic Gene Cluster
Source: PLoS Genet. 2013 Jun 20;9(6):e1003496. doi: 10.1371/journal.pgen.1003496 (PMC3688495; doi:10.1371/journal.pgen.1003496)
Supplement: Figure S2 — GO-Slim profiles (Aspergillus GO-Slim) for 14 hypocrealean taxa analyzed and for species-unique genes in the insect pathogens (Figure 2). A, C, E - GO Slim profiles for hypocrealean taxa; A) molecular function, C) biological process, and E) cellular component categories. Taxa from inside of circle to outside of circle are F. oxysporum, F. verticillioides, F. graminearum, N. haematococca, Tr. atroviride, Tr. reesei, Tr. virens, C. militaris, M. acridum, M. robertsii, and T. inflatum. B, D, F - GO Slim profiles for species-unique genes in (from inside to out) C. militaris, M. robertsii, M. acridum, and T. inflatum for B) molecular function, D) biological process, and F) cellular component. Percent of genes in each category out of total annotated genes analyzed is shown. (PDF) [file pgen.1003496.s002.pdf]

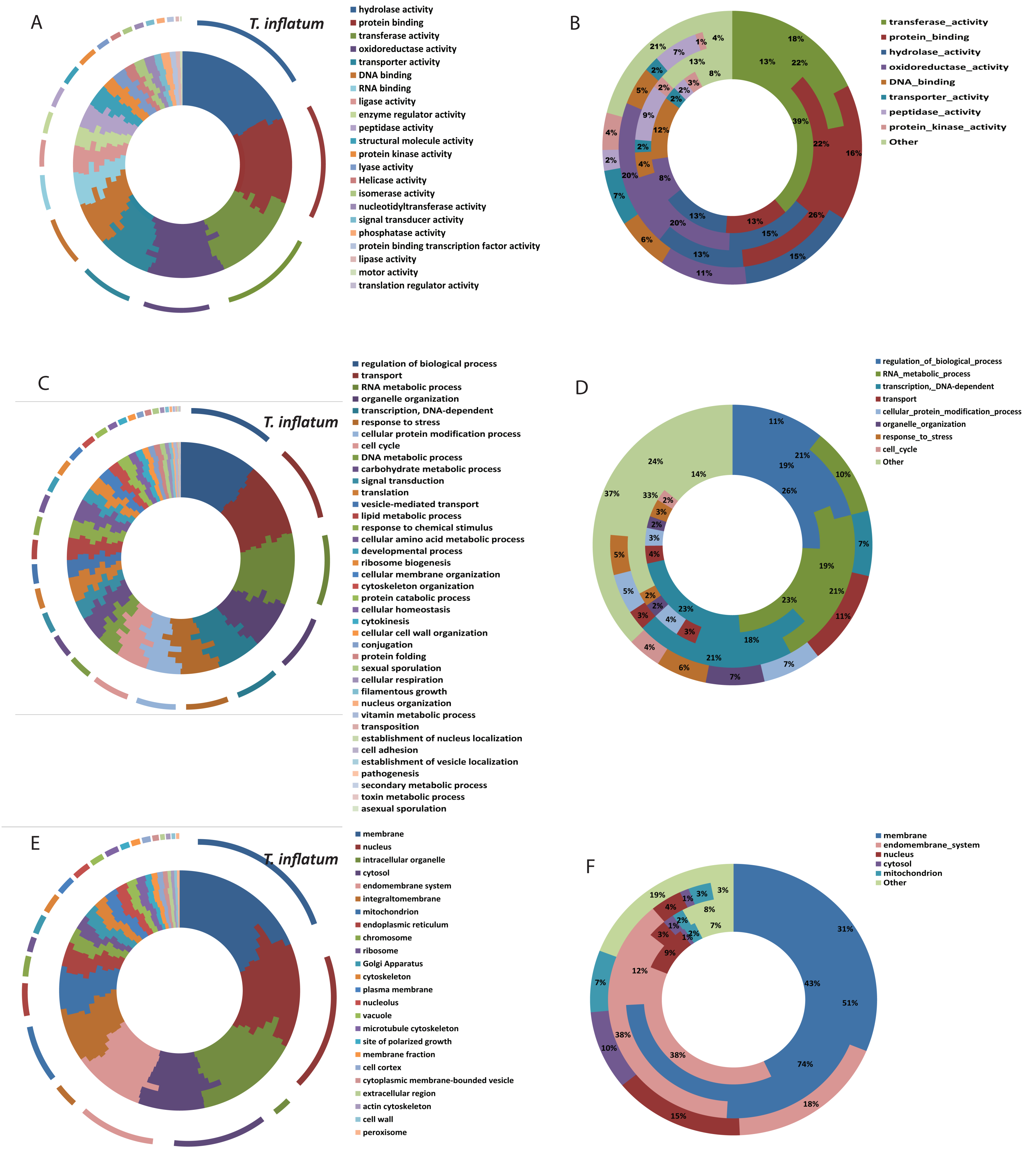
A
T. inflatum
hydrolase activity
protein binding
transferase activity
oxidoreductase activity
transporter activity
DNA binding
RNA binding
ligase activity
enzyme regulator activity
peptidase activity
structural molecule activity
protein kinase activity
lyase activity
Helicase activity
isomerase activity
nucleotidyltransferase activity
signal transducer activity
phosphatase activity
protein binding transcription factor activity
lipase activity
motor activity
translation regulator activity
B
transferase_activity
protein_binding
hydrolase_activity
oxidoreductase_activity
DNA_binding
transporter_activity
peptidase_activity
protein_kinase_activity
Other
18%
22%
13%
4%
1%
21%
7%
13%
8%
3%
2%
2%
2%
5%
9%
12%
39%
22%
16%
4%
2%
2%
4%
20%
8%
7%
13%
13%
26%
15%
20%
6%
13%
15%
11%
C
T. inflatum
regulation of biological process
transport
RNA metabolic process
organelle organization
transcription, DNA-dependent
response to stress
cellular protein modification process
cell cycle
DNA metabolic process
carbohydrate metabolic process
signal transduction
translation
vesicle-mediated transport
lipid metabolic process
response to chemical stimulus
cellular amino acid metabolic process
developmental process
ribosome biogenesis
cellular membrane organization
cytoskeleton organization
protein catabolic process
cellular homeostasis
cytokinesis
cellular cell wall organization
conjugation
protein folding
sexual sporulation
cellular respiration
filamentous growth
nucleus organization
vitamin metabolic process
transposition
establishment of nucleus localization
cell adhesion
establishment of vesicle localization
pathogenesis
secondary metabolic process
toxin metabolic process
asexual sporulation
D
regulation_of_biological_process
RNA_metabolic_process
transcription,_DNA-dependent
transport
cellular_protein_modification_process
organelle_organization
response_to_stress
cell_cycle
Other
11%
21%
19%
10%
26%
14%
24%
37%
33%
2%
3%
2%
3%
7%
4%
19%
5%
21%
5%
2%
23%
2%
23%
11%
3%
4%
3%
18%
4%
21%
7%
6%
7%
E
T. inflatum
membrane
nucleus
intracellular organelle
cytosol
endomembrane system
integraltomembrane
mitochondrion
endoplasmic reticulum
chromosome
ribosome
Golgi Apparatus
cytoskeleton
plasma membrane
nucleolus
vacuole
microtubule cytoskeleton
site of polarized growth
membrane fraction
cell cortex
cytoplasmic membrane-bounded vesicle
extracellular region
actin cytoskeleton
cell wall
peroxisome
F
membrane
endomembrane_system
nucleus
cytosol
mitochondrion
Other
3%
3%
19%
1%
4%
8%
2%
1%
7%
31%
3%
2%
1%
9%
12%
7%
43%
51%
38%
38%
10%
74%
18%
15%
