# Supplementary material for: The Genome of Tolypocladium inflatum: Evolution, Organization, and Expression of the Cyclosporin Biosynthetic Gene Cluster
Source: PLoS Genet. 2013 Jun 20;9(6):e1003496. doi: 10.1371/journal.pgen.1003496 (PMC3688495; doi:10.1371/journal.pgen.1003496)
Supplement: Figure S4 — Possible homologs of ergot alkaloid biosynthetic genes in T. inflatum and Metarhizium spp. A) NRPS A-domains in other Hypocreales related to C. purpurea ergot alkaloid synthetases cpps1-cpps4 and belonging to the ergot alkaloid clade in the larger phylogeny (Figure S3). The C. purpurea monomodular NRPSs (cpps2 and cpps3) are shown in light green while the trimodular cpps1 and cpps4 are shown in dark green. Both M. robertsii (red) and M. acridum (pink) contain orthologs of the two monomodular NRPSs (cpps2 and cpps3) as well as a novel 7 modular NRPS showing closer similarity to cpps1 and cpps4. T. inflatum (orange) lacks both monomodular NRPSs but contains an NRPS with 4 modules whose A-domains also are most similar to cpps1 and cpps4 (orange). B) The antiSMASH predicted secondary metabolite gene clusters containing these NRPSs show that in addition to orthologs of the two monomodular C. purpurea NRPSs cpps2 (MAA_06742 and MAC_06982) and cpps3 (MAA_06744 and MAC_06980) both M. robertsii and M. acridum also contain orthologs (indicated by vertical lines and color coding – same color = homologs, black = not homologs) of the majority of genes found on the 5′ end of the C. purpurea ergot alkaloid cluster (shaded light green). Two 7 modular NRPSs in Metarhizium spp. (MAA_06559 and MAC_08899) that share homology to A-domains of the 3 modular genes (cpps1 and cpps4) in C. purpurea are located in a distinct cluster that does not contain other genes from the ergot cluster. T. inflatum appears to lack homologs of the two monomodular NRPSs (cpps2, cpps3) and other genes in the 5′portion of the cluster but contains a single 4-modular NRPS (TINF02556) containing A-domains that group with C. purpurea NRPSs cpps1 and cpps4. The antiSMASH predicted cluster containing TINF02556 is shown and contains other genes of unknown function predicted to be involved in secondary metabolism but lacks homologs of the ergot alkaloid biosynthetic pathway. C) A single DMAT enzyme is found in the T. [file pgen.1003496.s004.pdf]

A

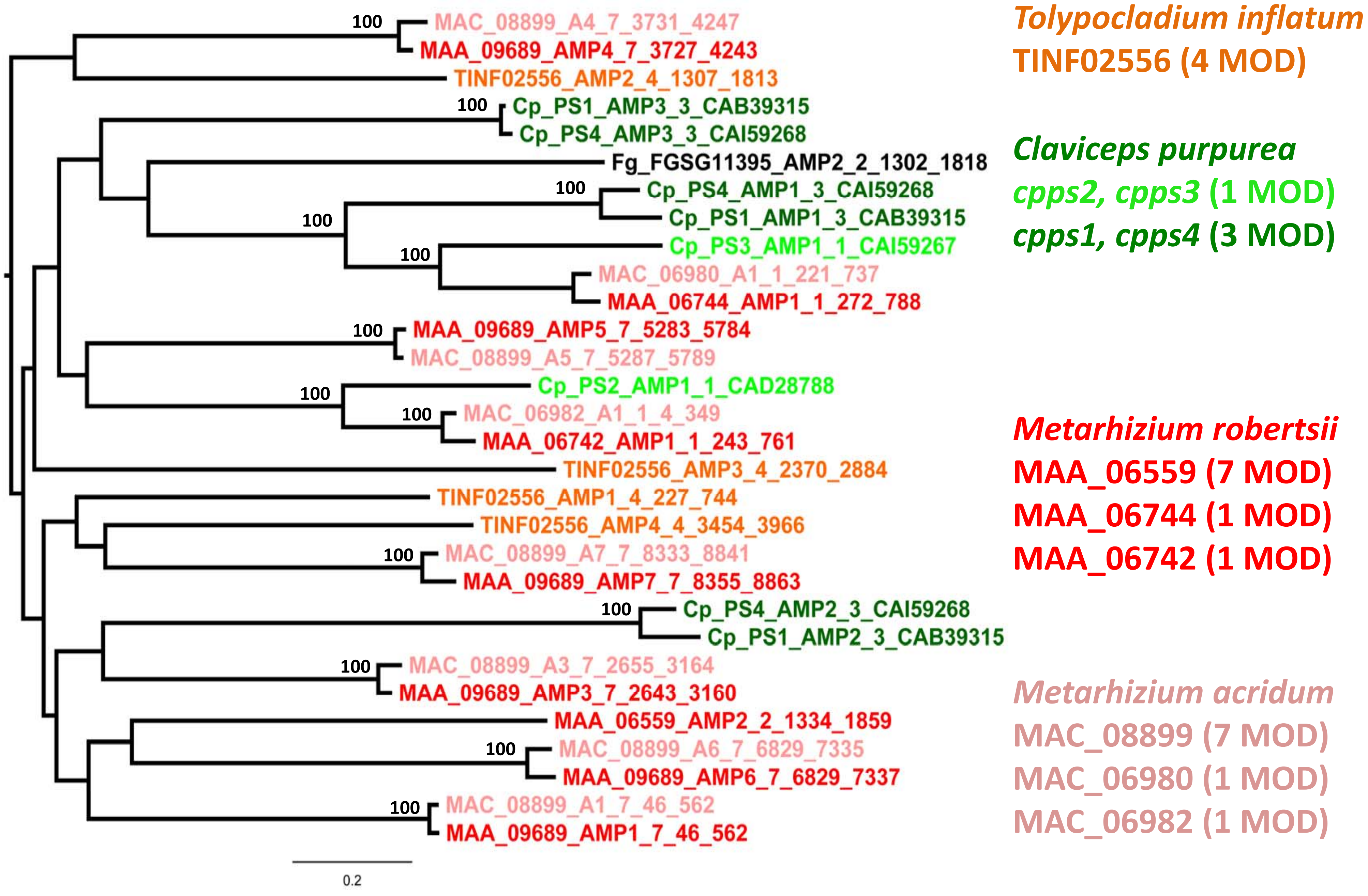

100
MAC_08899_A4_7_3731_4247
MAA_09689_AMP4_7_3727_4243
TINF02556_AMP2_4_1307_1813
100
Cp_PS1_AMP3_3_CAB39315
Cp_PS4_AMP3_3_CAI59268
Fg_FGSG11395_AMP2_2_1302_1818
100
Cp_PS4_AMP1_3_CAI59268
100
Cp_PS1_AMP1_3_CAB39315
Cp_PS3_AMP1_1_CAI59267
100
MAC_06980_A1_1_221_737
MAA_06744_AMP1_1_272_788
100
MAA_09689_AMP5_7_5283_5784
MAC_08899_A5_7_5287_5789
Cp_PS2_AMP1_1_CAD28788
100
MAC_06982_A1_1_4_349
100
MAA_06742_AMP1_1_243_761
TINF02556_AMP3_4_2370_2884
TINF02556_AMP1_4_227_744
TINF02556_AMP4_4_3454_3966
100
MAC_08899_A7_7_8333_8841
MAA_09689_AMP7_7_8355_8863
100
Cp_PS4_AMP2_3_CAI59268
Cp_PS1_AMP2_3_CAB39315
100
MAC_08899_A3_7_2655_3164
MAA_09689_AMP3_7_2643_3160
MAA_06559_AMP2_2_1334_1859
100
MAC_08899_A6_7_6829_7335
MAA_09689_AMP6_7_6829_7337
100
MAC_08899_A1_7_46_562
MAA_09689_AMP1_7_46_562
0.2
Tolypocladium inflatum
TINF02556 (4 MOD)
Claviceps purpurea
cpps2, cpps3 (1 MOD)
cpps1, cpps4 (3 MOD)
Metarhizium robertsii
MAA_06559 (7 MOD)
MAA_06744 (1 MOD)
MAA_06742 (1 MOD)
Metarhizium acridum
MAC_08899 (7 MOD)
MAC_06980 (1 MOD)
MAC_06982 (1 MOD)

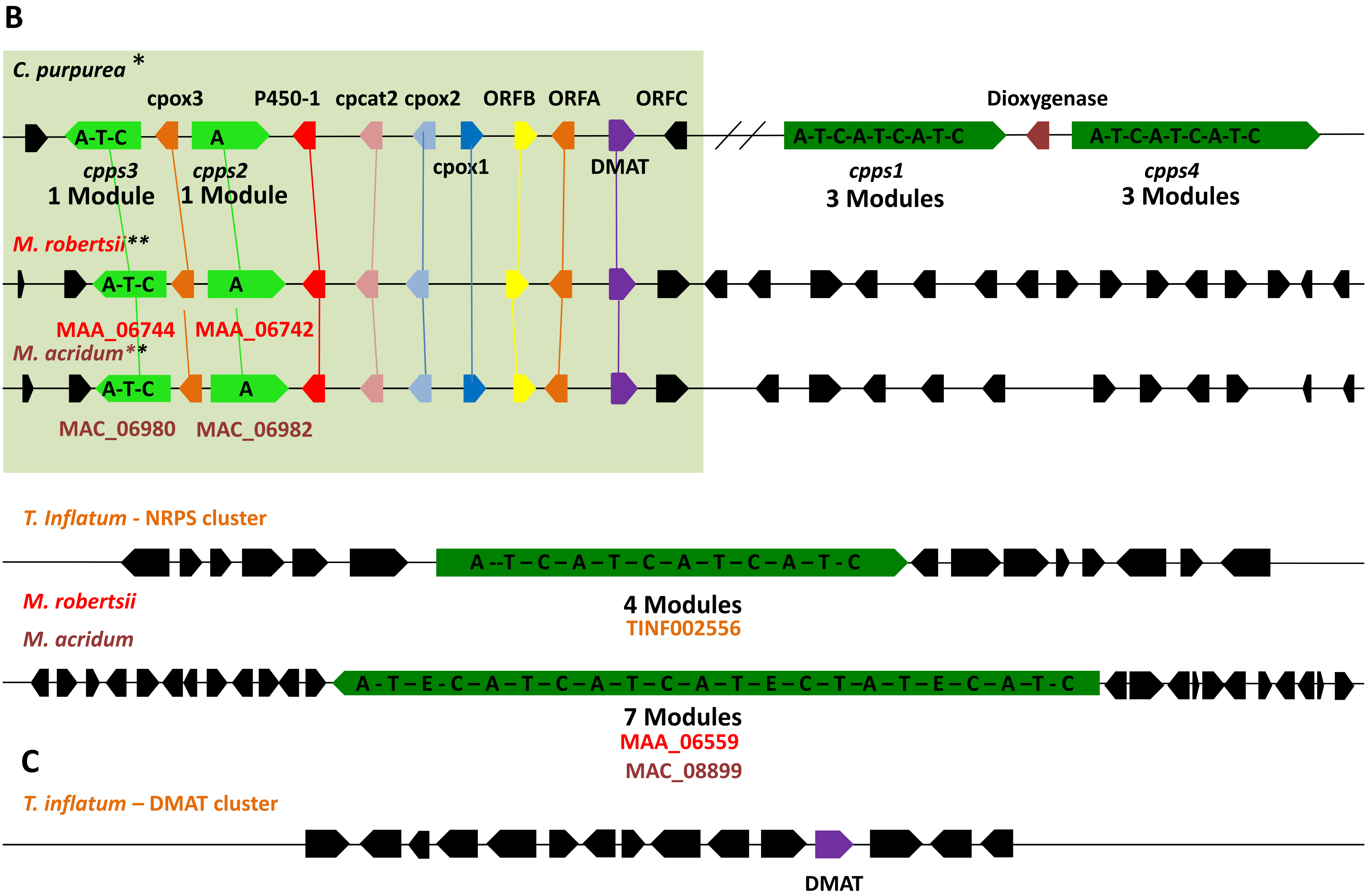

* Pannacione, 2005. Fems Microbiology Letters. 251 (1):9-17.

** Gao et. al., 2011. PLoS Genetics. 7(1): 1-17.
