# Supplementary material for: The Genome of Tolypocladium inflatum: Evolution, Organization, and Expression of the Cyclosporin Biosynthetic Gene Cluster
Source: PLoS Genet. 2013 Jun 20;9(6):e1003496. doi: 10.1371/journal.pgen.1003496 (PMC3688495; doi:10.1371/journal.pgen.1003496)
Supplement: Figure S6 — A) left panel: relative expression levels (reads mapped to genes/total mapped reads in treatment) of each biological replicate in SM medium at each time point, right panel: complete HPLC traces of extracts from pooled samples at same time points in SM medium showing cyclsoporin A peak at 38 min. (marked by a red asterisk); B) left panel: relative expression levels (reads mapped to gene/total mapped reads in treatment) of each biological replicate in SDB medium at each time point and right panel: complete HPLC traces of extracts from pooled samples at same time point in SDB media showing only trace amounts of cyclosporin A in the peak at 38 min. (marked by a red asterisk). (PDF) [file pgen.1003496.s006.pdf]

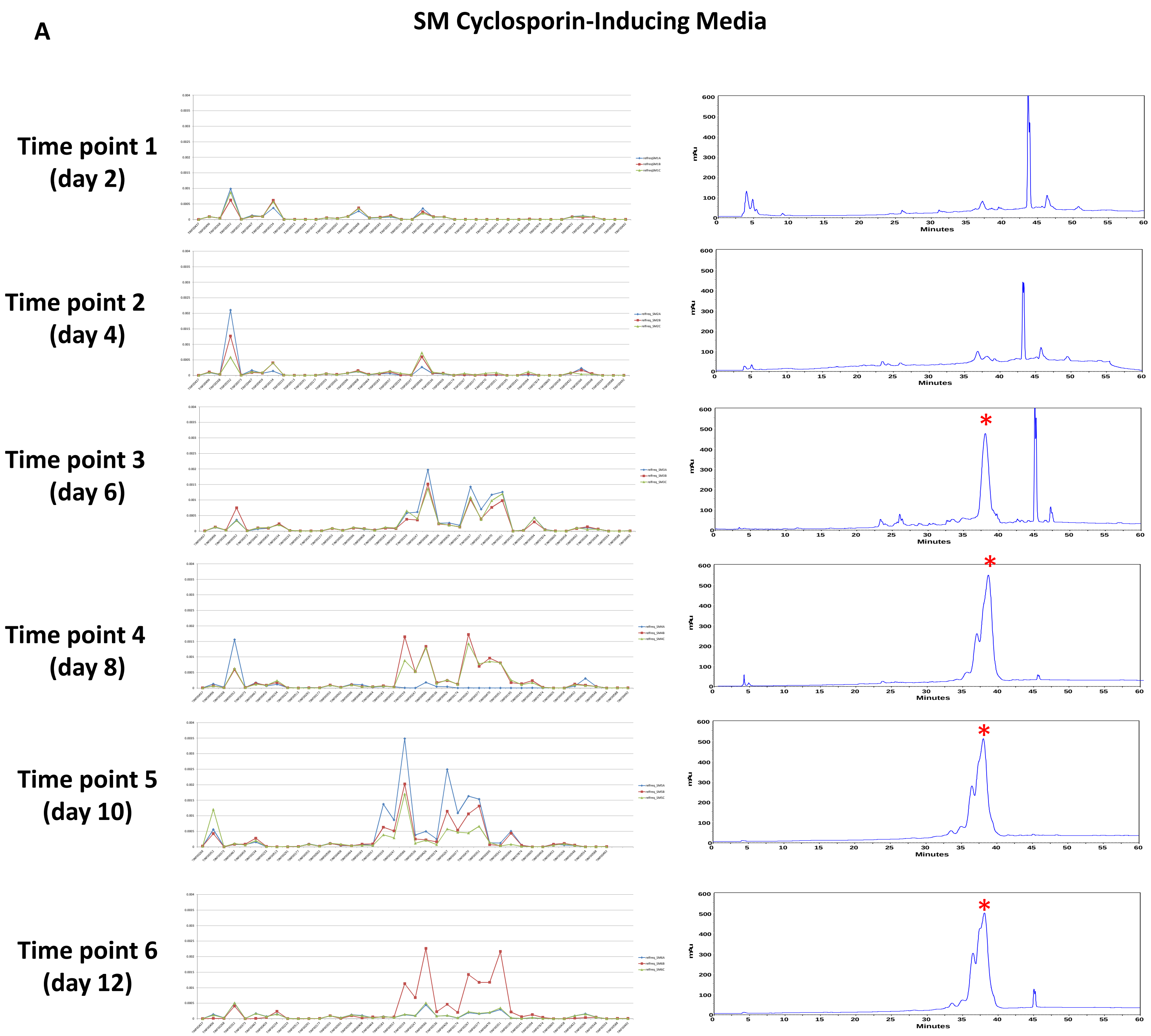
A
SM Cyclosporin-Inducing Media
Time point 1
(day 2)
Time point 2
(day 4)
Time point 3
(day 6)
Time point 4
(day 8)
Time point 5
(day 10)
Time point 6
(day 12)
mAu
Minutes
*

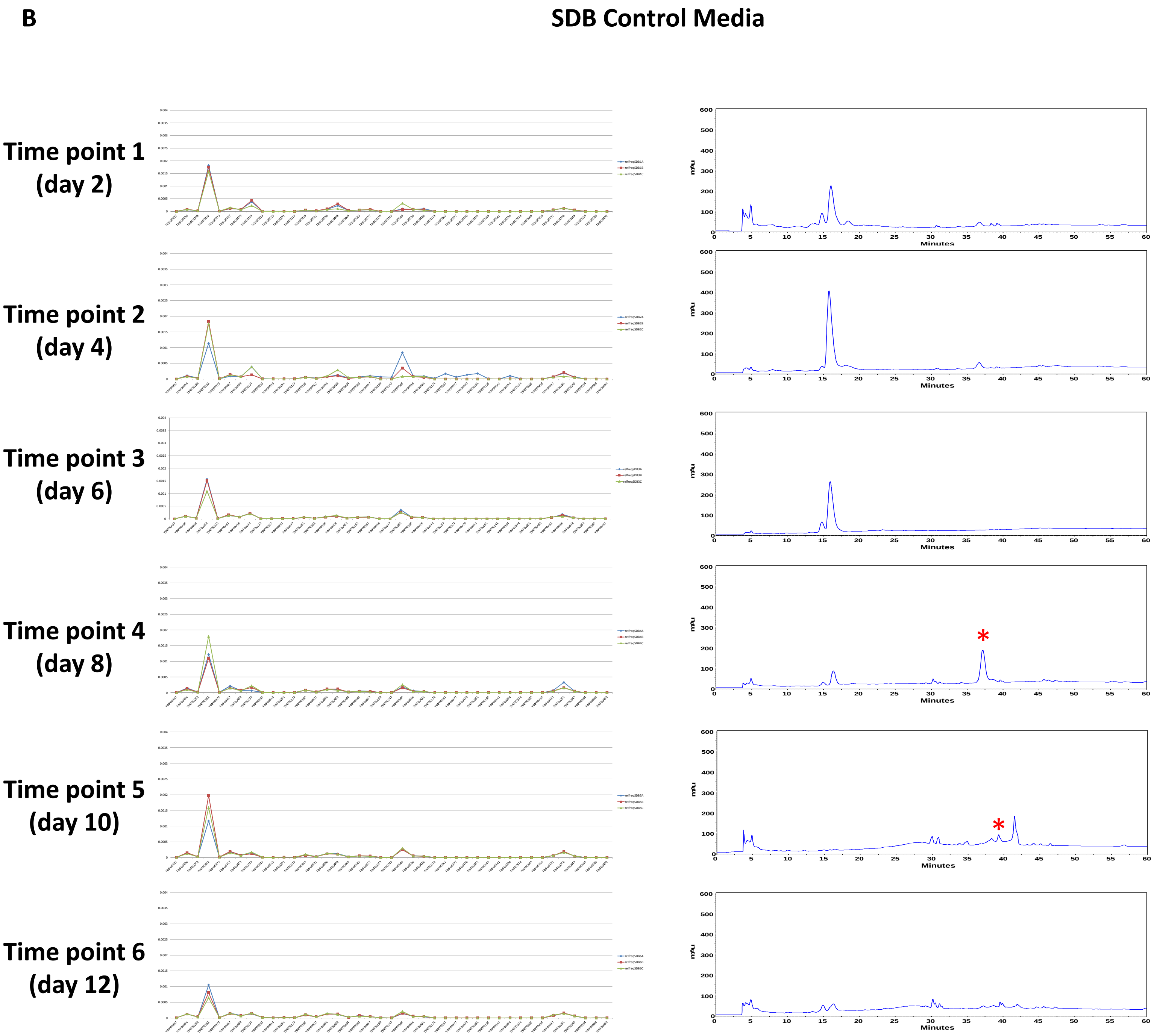

B
SDB Control Media
Time point 1
(day 2)
Time point 2
(day 4)
Time point 3
(day 6)
Time point 4
(day 8)
Time point 5
(day 10)
Time point 6
(day 12)
Minutes
*
*
