# Supplementary material for: The Genome of Tolypocladium inflatum: Evolution, Organization, and Expression of the Cyclosporin Biosynthetic Gene Cluster
Source: PLoS Genet. 2013 Jun 20;9(6):e1003496. doi: 10.1371/journal.pgen.1003496 (PMC3688495; doi:10.1371/journal.pgen.1003496)

Gene1 TINF00496

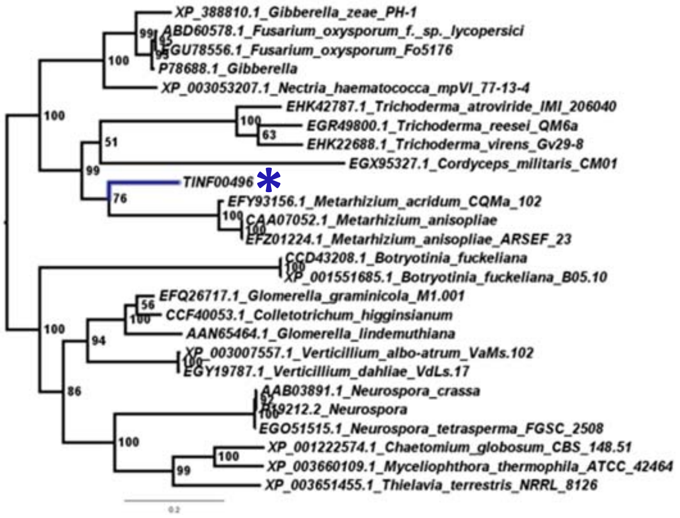

Gene2 TINF00268

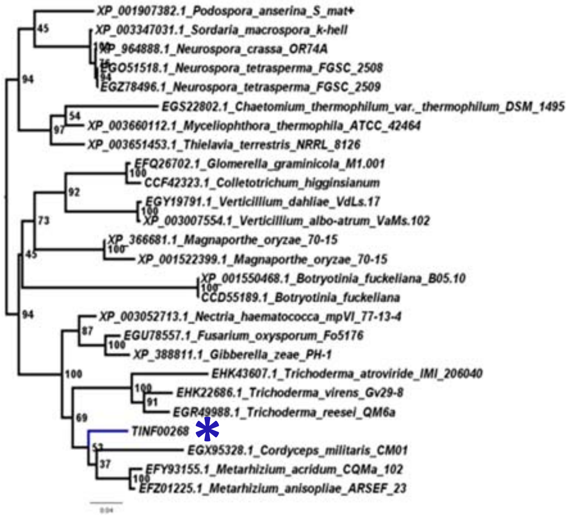

Gene3 TINF00352

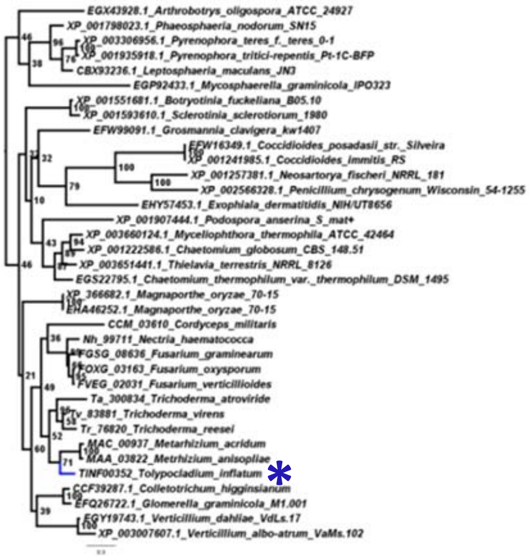

Gene4 TINF00373 – no hits >e-^{05}

Gene5 TINF00467

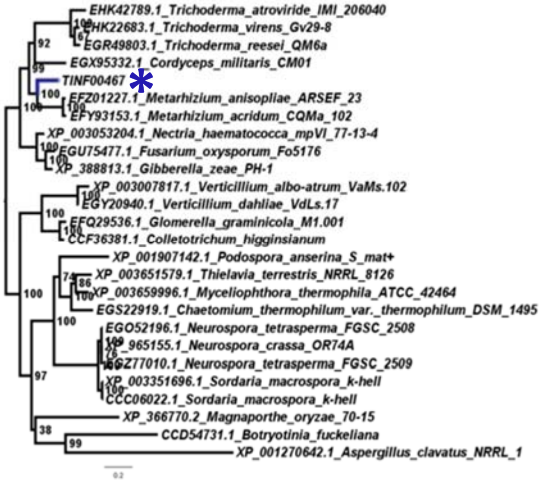

Gene6 TINF00459

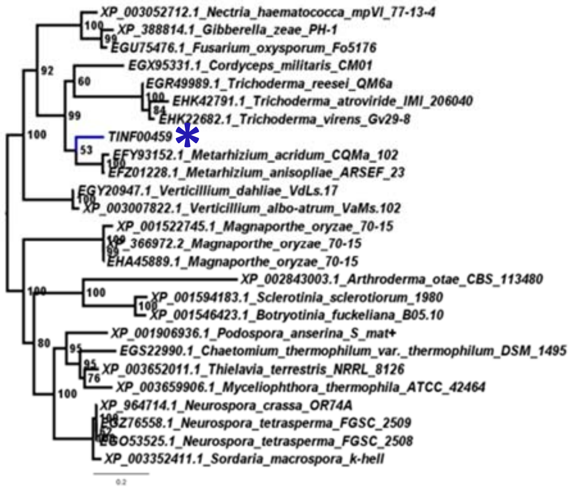

Gene7 TINF00234

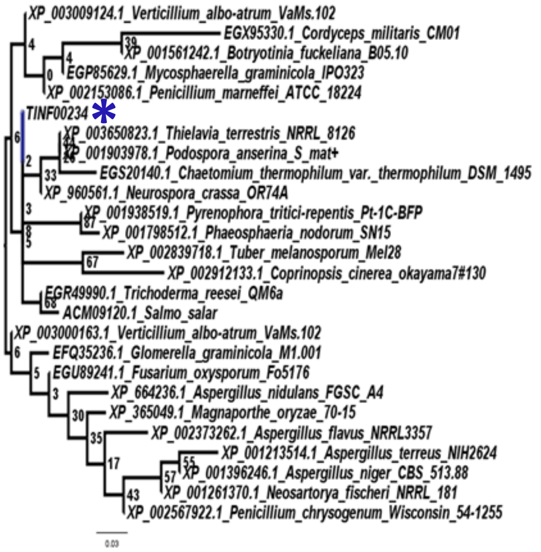

Gene8 TINF00233

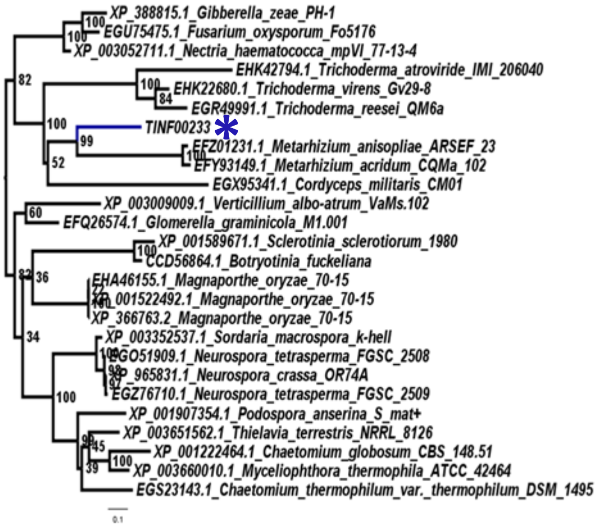

Gene9 TINF00513

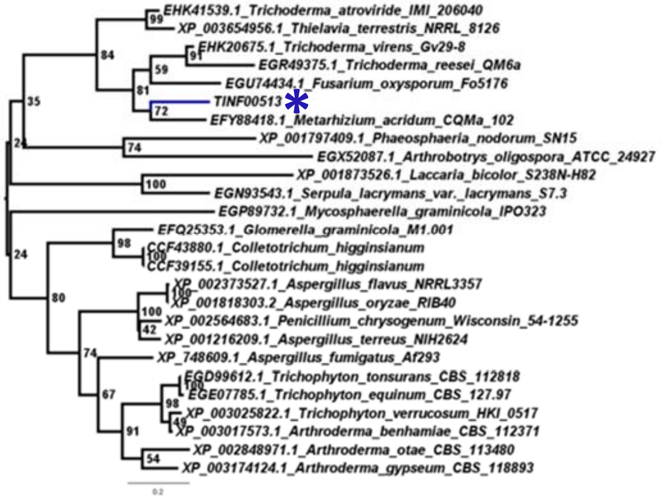

Gene10 TINF00291

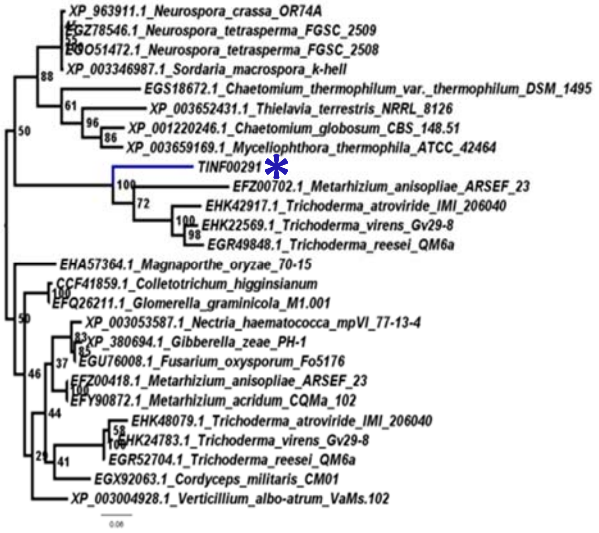

Gene11 TINF00177

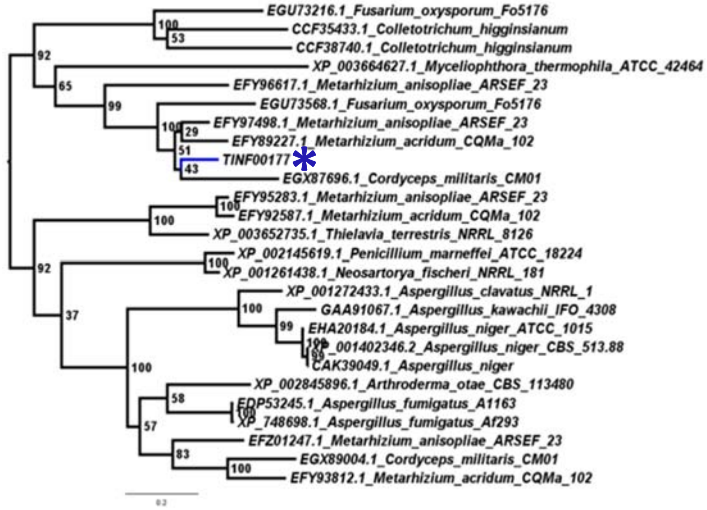

Gene12 TINF00355

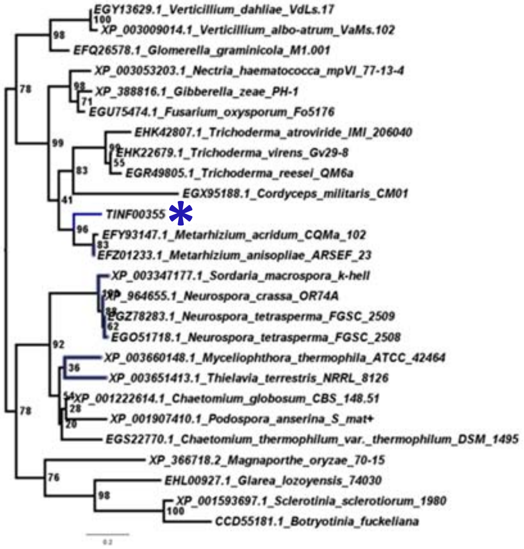

Gene13 TINF00502

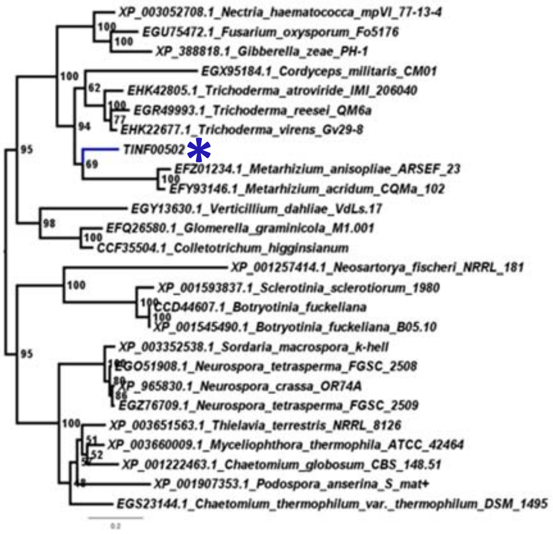

Gene14 TINF00596

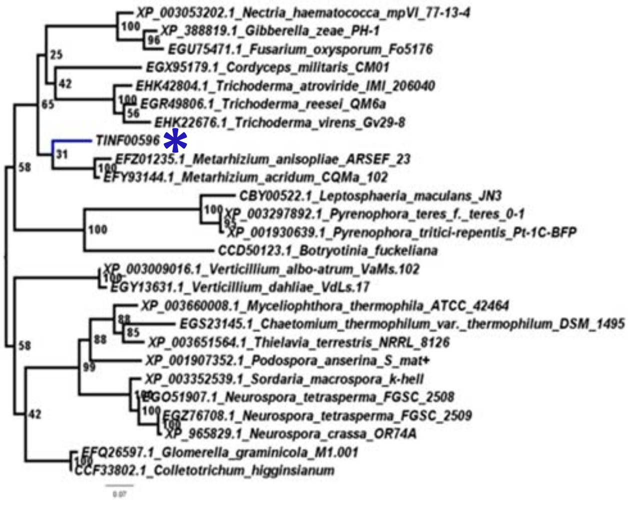

Gene15 TINF00408

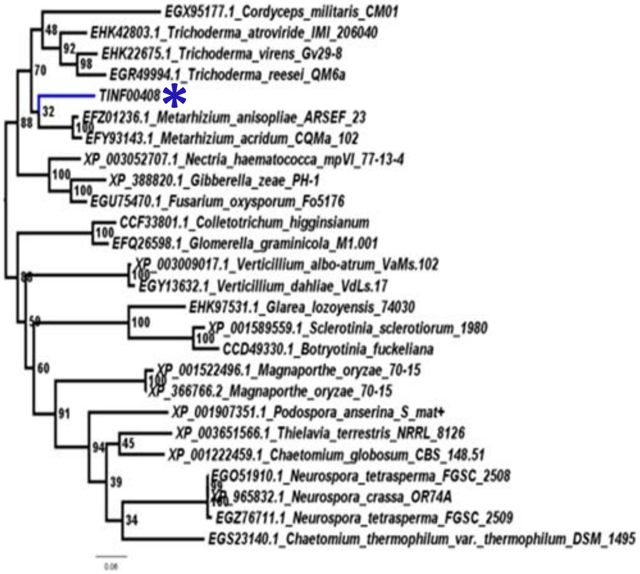

Gene16 TINF00464

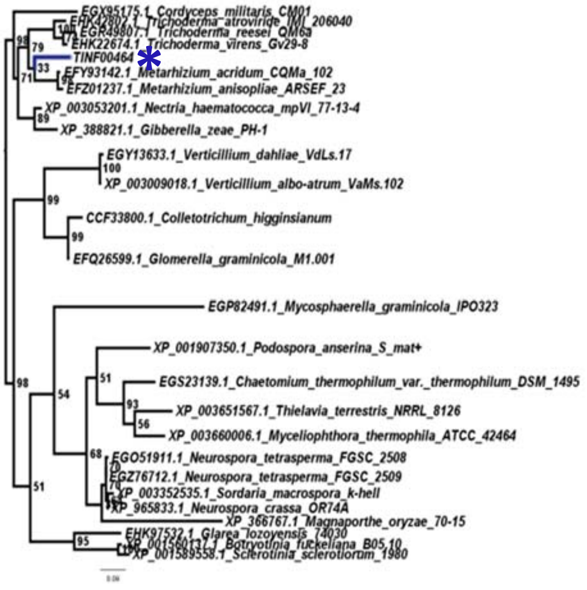

Gene17 TINF00183

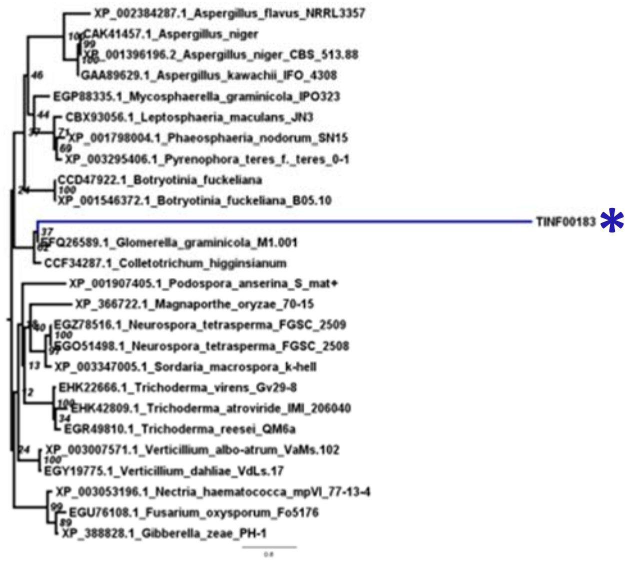

Gene18 TINF00557

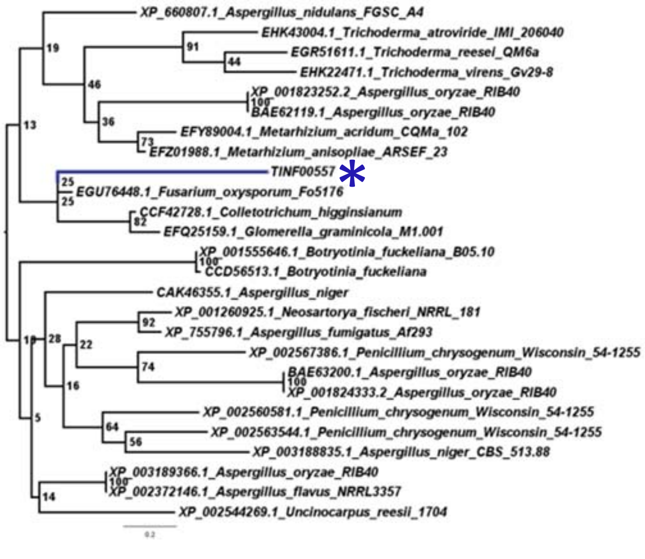

Gene19 TINF00159

See Figure S5

Gene20 TINF00247

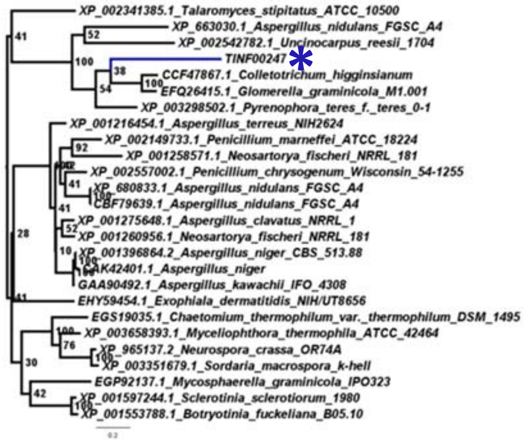

Gene21 TINF00586

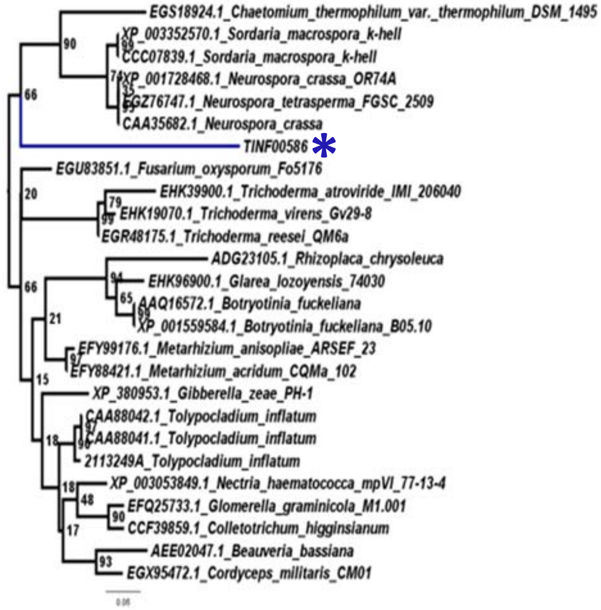

Gene22 TINF00536

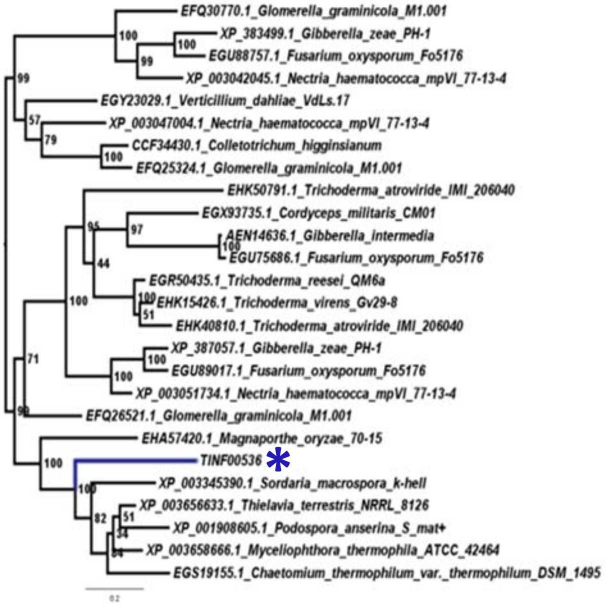

Gene23 TINF00426

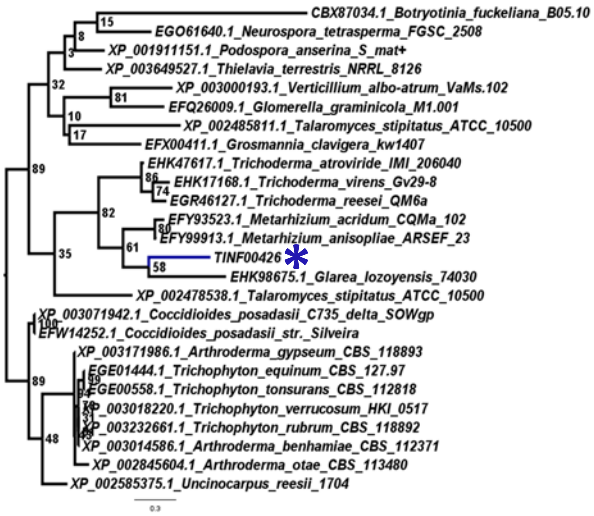

Gene24 TINF00174

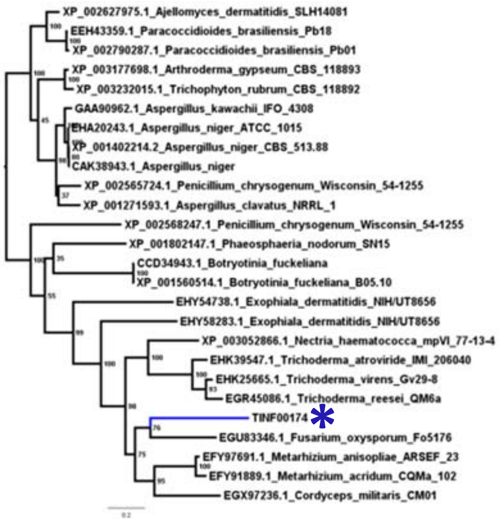

Gene25 TINF00267

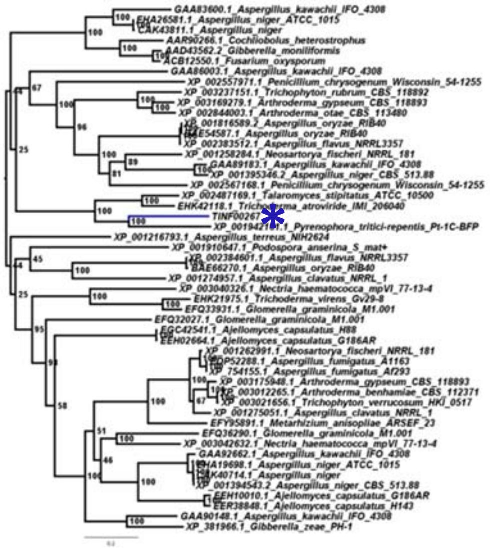

Gene26 TINF00377

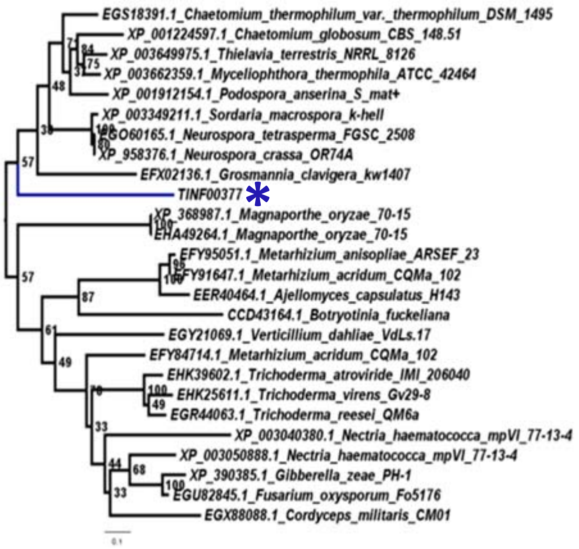

Gene27 TINF00470

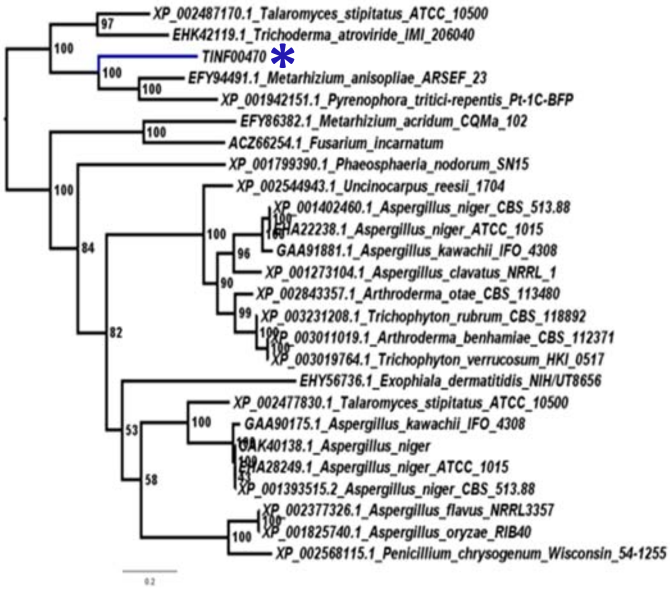

Gene28 TINF00351

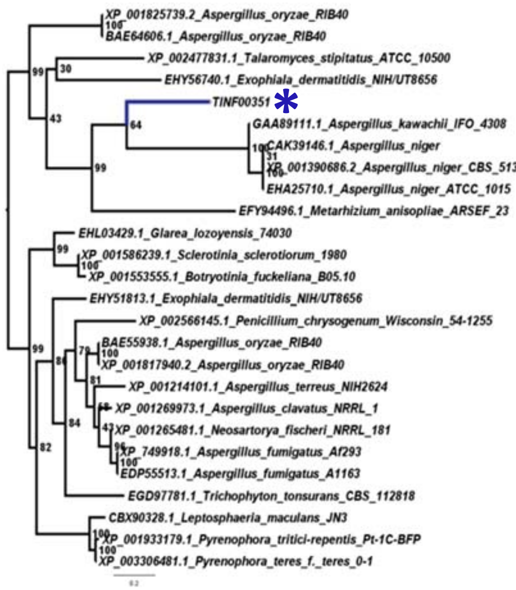

Gene29 TINF00195

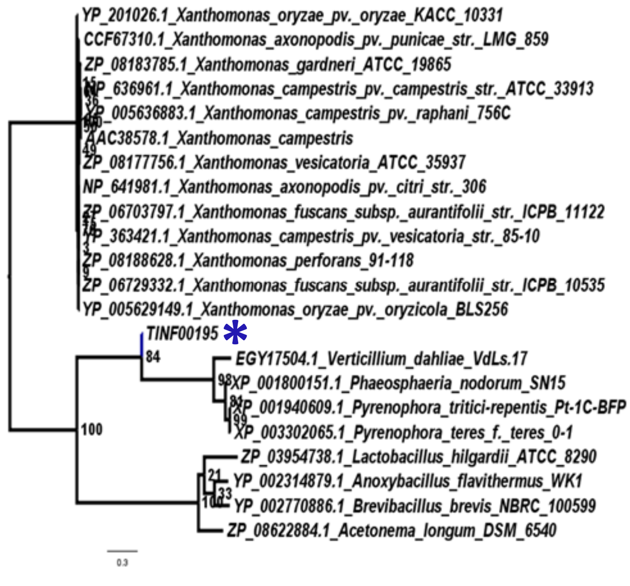

Gene30 TINF00141

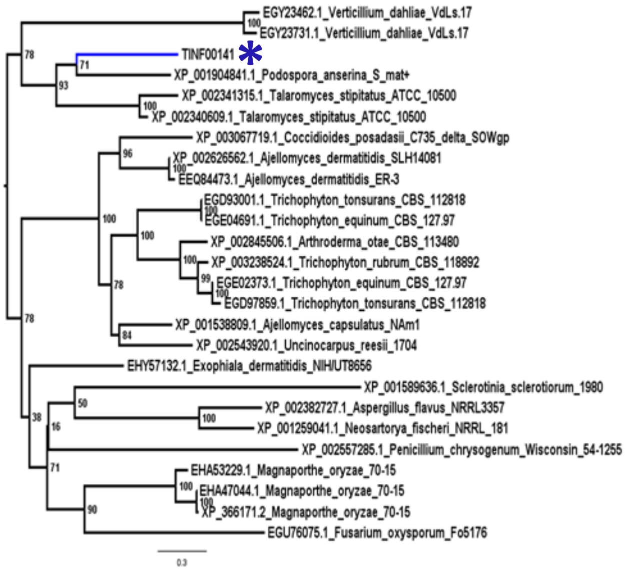

Gene31 TINF00394

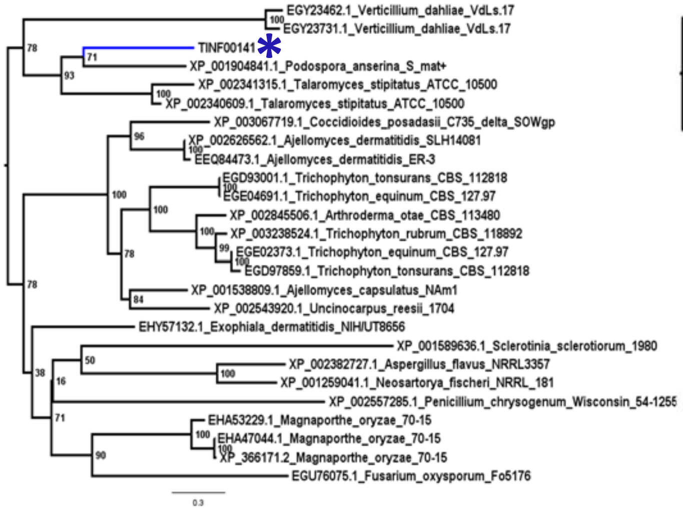

Gene32 TINF007874

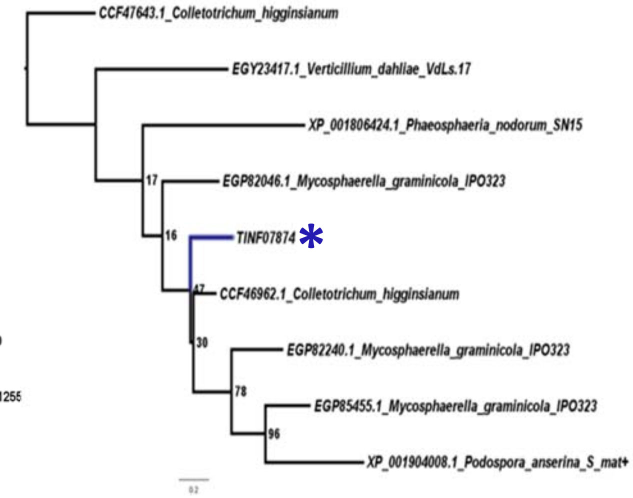

Gene33 TINF00620

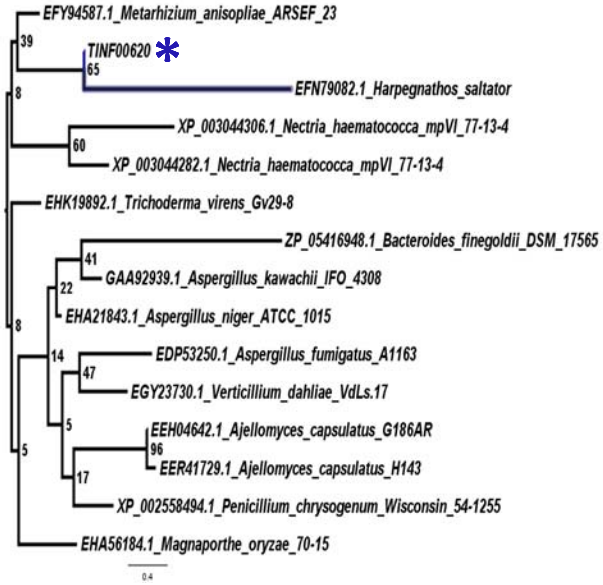

Gene34 TINF00605

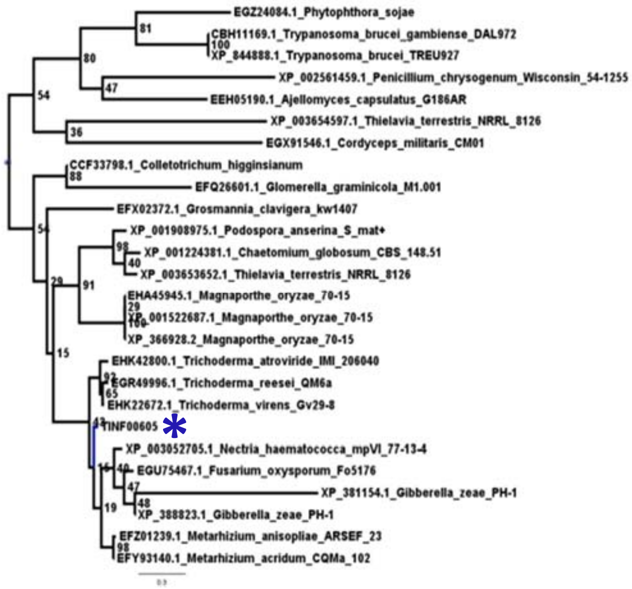

Gene35 TINF00458

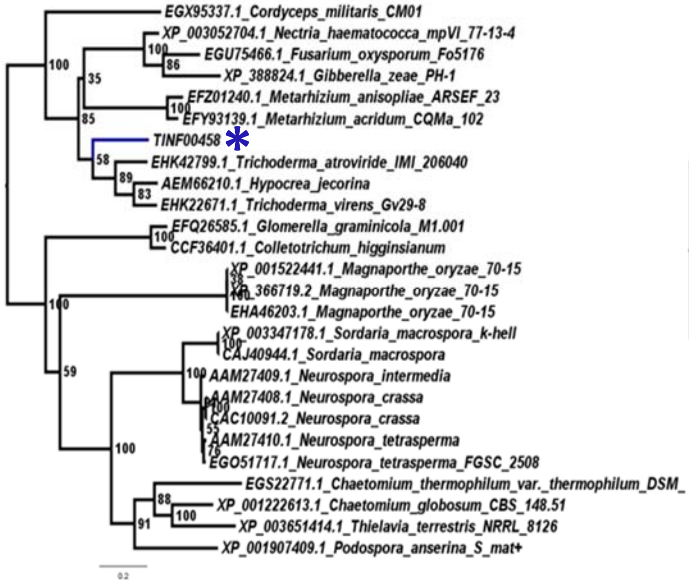

Gene36 TINF00432

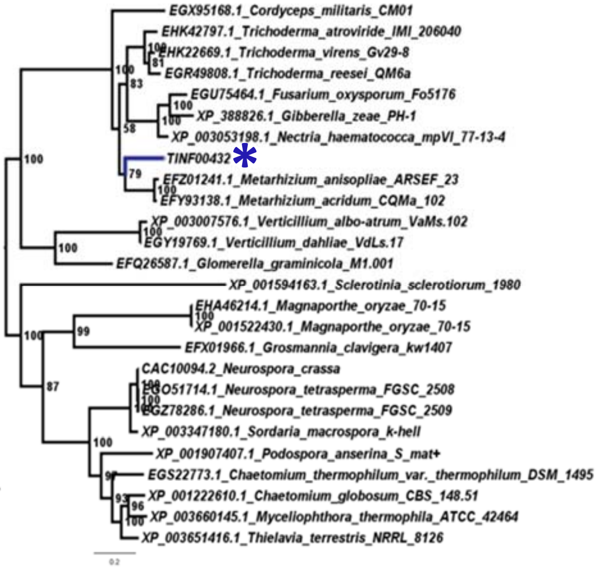

Gene37 TINF00266

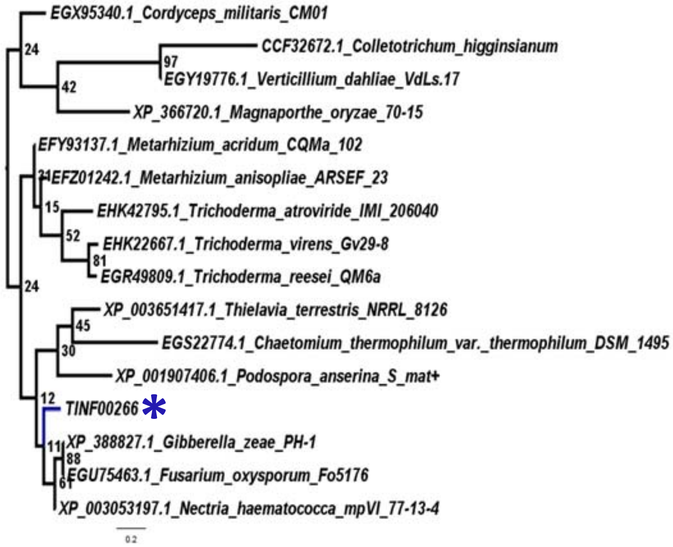

Gene38 TINF00548

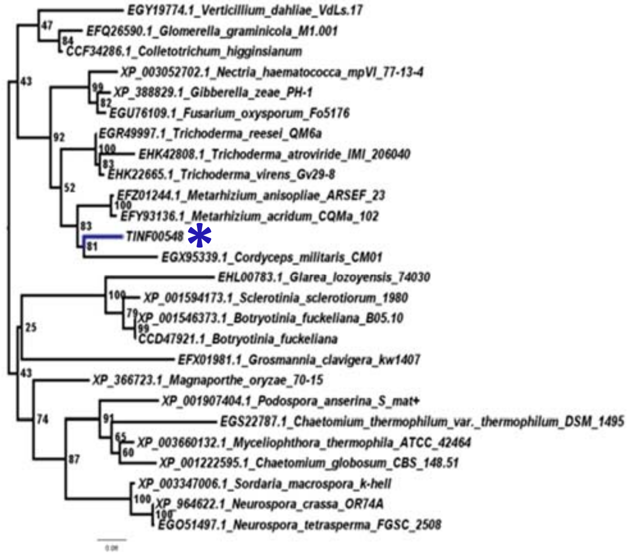

Gene39 TINF00185

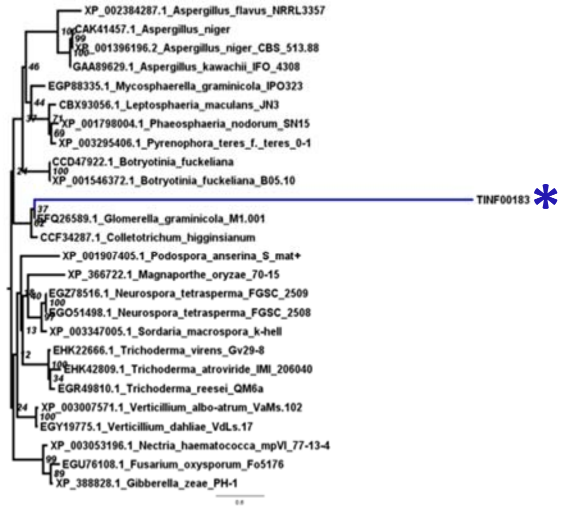

Gene40 TINF00554

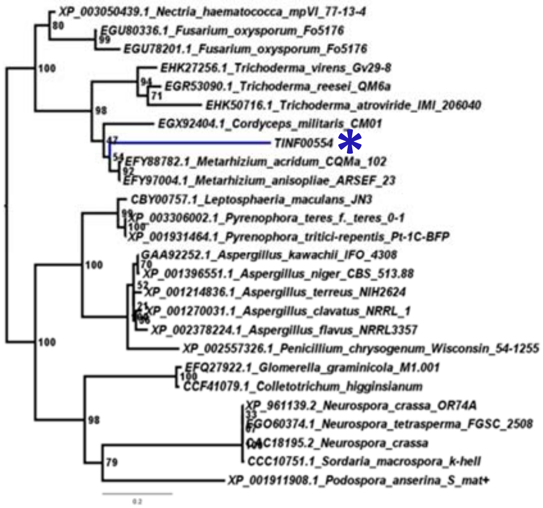

Gene41 TINF00588

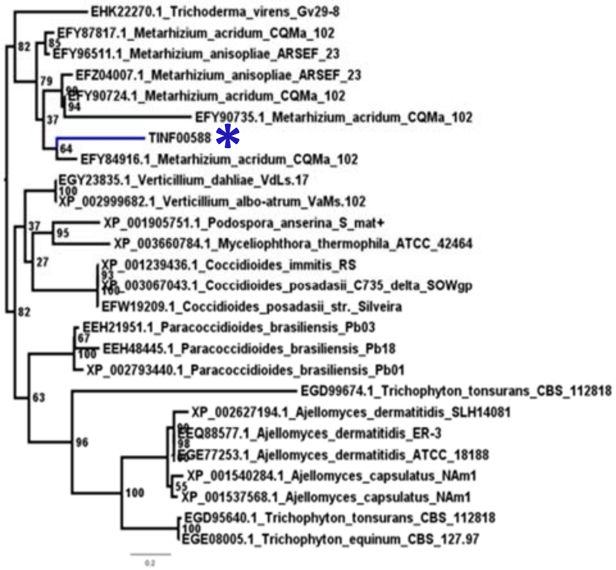

Gene42 TINF00492

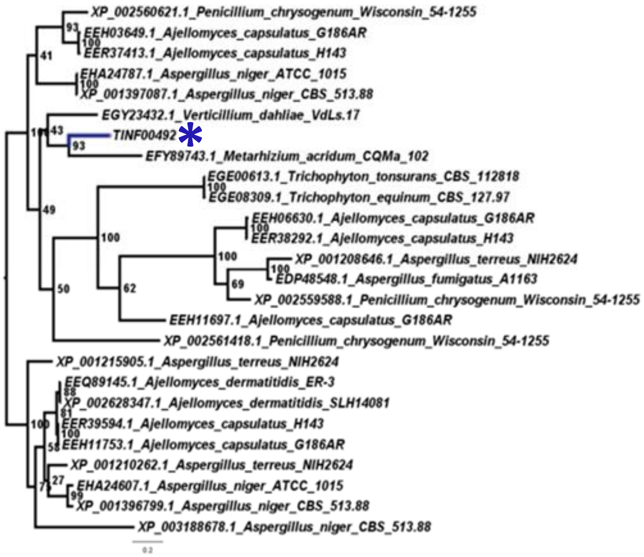

Supplement: Figure S8 — Maximum likelihood phylogenies of top 25 BLAST hits to each gene in the simA cluster plus 10 genes flanking the antiSMASH predicted cluster from the 5′ to 3′ end. Most T. inflatum genes (branches shown in blue) that are located outside of the RNA-Seq defined metabolite cluster have single copy orthologs in other hypocrealean taxa, while those inside the cluster mostly lack orthologs in other hypocrealean taxa. (PDF) [file pgen.1003496.s008.pdf]
