# Supplementary material for: The Genome of Tolypocladium inflatum: Evolution, Organization, and Expression of the Cyclosporin Biosynthetic Gene Cluster
Source: PLoS Genet. 2013 Jun 20;9(6):e1003496. doi: 10.1371/journal.pgen.1003496 (PMC3688495; doi:10.1371/journal.pgen.1003496)
Supplement: Table S1 — A) Numbers of major classes of fungal repeat elements in the T. inflatum genome characterized by RepeatMasker. The T. inflatum genome contains a large number of DNA hAT transposons. B) Number of CPA element and Restless transposons found in other hypocrealean taxa. (DOCX) [file pgen.1003496.s009.docx]

**Table S1| Repeat Elements**

1. Repeat Elements in *T. inflatum* characterized by RepeatMasker version 3.2.8 using the fungal species library (library version 20090604) and and crossmatch 0.990329 with the default settings.

| Repeat Element | Coverage of Repeat in bp | Numbers of Repeats* |
| --- | --- | --- |
| DNA-hAT | 12967 | 62 |
| DNA-MuDR | 6697 | 9 |
| DNA-Mariner | 7340 | 13 |
| LTR-Copia | 4336 | 12 |
| LTR-Gypsy | 42665 | 26 |
| LINE | 32441 | 63 |
| Other Non-LTR | 524 | 3 |
| Unclassified | 1452 | 7 |
| Simple Repeats | 151399 | 2743 |
| Satellites | 97 | 1 |
| small RNA | 6510 | 33 |
| Low Complexity DNA | 110388 | 2318 |

1. Distribution of *CPA* element and *Restles*s across hypocrealean taxa

|  | *T. inflatum* | *M. robbertsii* | *M. acridum* | *C. militaris* | *Tr. virens* | *Tr. atroviride* | *Tr. reeseii* | *F. graminearum* | *F. oxysporum* | *F. verticillioides* | *N. haematococca* |
| --- | --- | --- | --- | --- | --- | --- | --- | --- | --- | --- | --- |
| CPA | 12 | 5 | 5 | 1 | 1 | 0 | 2 | 0 | 15 | 0 | 27 |
| *Restless* | 26 | 11 | 0 | 1 | 1 | 0 | 0 | 0 | 55 | 0 | 8 |
| *Restless* delta2 | 1 | 0 | 0 | 0 | 0 | 0 | 0 | 0 | 0 | 0 | 0 |
| *Restless* delta3 | 2 | 1 | 1 | 0 | 0 | 0 | 1 | 1 | 1 | 1 | 1 |
| *Restless* delta4 | 3 | 0 | 0 | 0 | 0 | 0 | 0 | 0 | 0 | 0 | 0 |
| *Restless*  Delta 6 | 0 | 1 | 0 | 0 | 0 | 0 | 0 | 0 | 0 | 0 | 0 |

*While RepeatMasker aims to report most repeat elements fragmented by insertions or deletions as a single element, some may represent partial elements.
