# Supplementary material for: The Genome of Tolypocladium inflatum: Evolution, Organization, and Expression of the Cyclosporin Biosynthetic Gene Cluster
Source: PLoS Genet. 2013 Jun 20;9(6):e1003496. doi: 10.1371/journal.pgen.1003496 (PMC3688495; doi:10.1371/journal.pgen.1003496)
Supplement: Table S2 — Results from CAFÉ analyses of gene family expansions and contractions of CAZys, P450s, and proteases across the fourteen hypocrealean taxa and nodes in the phylogeny (Figure 3) are shown across the top and color coded according to ecology or predicted ancestral ecology (red = animal associated, blue = fungal associated, green = plant associated). Numbers of genes found in each taxa are listed in each cell and taxa or nodes that have significant expansions (E) or contractions (C) at p<0.01are shaded with gray or hatched lines respectively. (DOCX) [file pgen.1003496.s010.docx]

**Table S2**| **CAFÉ analyses**. Significantly expanded or contracted families of proteases, carbohydrate active enzymes (CAZys), and cytochrome P450s in hypocrealean taxa.

Ultrametric tree used for CAFÉ analyses: (((Ti:158,(Ma:19,Mb:19):139):13,(Cm:165,(Ta:145,(Tv:76,Tr:76):69):20):6):7,((Fg:60,(Fo:13,Fv:13):47):65,Nh:125):53)

| **Gene Family** | **Family p-value**  **> .05** | ***T. inflatum*  (Ophiocordycipitaceae)** | ***M. acridum*** | ***M. robertsii*** | ***C. militaris* (Cordycipitaceae)** | ***Tr. atroviride*** | ***Tr. virens*** | ***Tr. reeseii*** | ***N. haematococca*** | ***F. graminearum*** | ***F. oxysporum*** | ***F. verticillioides*** | ***Nectriaceae (*Plant Pathogens-node 3)** | ***Metarhizium* spp. (Clavicipitaceae – node 7)** | ***Trichoderma spp.* (Fungal Pathogen*s -* node6*)*** | ***T. inflatum/Metarhizium* spp. (node 4)** |
| --- | --- | --- | --- | --- | --- | --- | --- | --- | --- | --- | --- | --- | --- | --- | --- | --- |
|  |  | Insect Pathogens | | | | Fungal Pathogens | | | Plant Pathogens | | | | Nodes in Figure 3A | | | |
| **Proteases** | | | | | | | | | | | | | | | | |
| All proteases (Merops) | 0 | 345  C | 398  E | 487  E | 405  ----- | 440  ----- | 473  E | 325  C | 608  E | 455  E | 616  E | 499  E | E |  |  |  |
| Secreted proteases (SignalP) | 0 | 96 | 118  E | 174  E | 115 | 98 | 74 | 69 | 157  E | 124 | 146  E | 121  E |  | E |  |  |
| **Serine proteases** |  |  |  |  |  |  |  |  |  |  |  |  |  |  |  |  |
| S33 | 0 | 26 | 30 | 30 | 22  C | 62 | 69  E | 33  C | 65 | 50 | 70  E | 47  C |  |  |  |  |
| S08 | 0 | 18 | 35  E | 51 | 21 | 44 | 38 | 20 | 39 | 34 | 34 | 30 |  |  |  |  |
| S15 | 0.002 | 2 | 0  C | 2 | 1 | 3 | 2 | 2 | 18  E | 4 | 10 | 13 | E |  |  |  |
| S59 | 0.001 | 1 | 1 | 1 | 1 | 1 | 1 | 1 | 1 | 1 | 10  E | 1 |  |  |  |  |
| S09 | 0 | 32 | 34 | 40 | 39 | 47 | 68  E | 35  C | 122  E | 70 | 111  E | 88 | E |  |  |  |
|  |  |  |  |  |  |  |  |  |  |  |  |  |  |  |  |  |
| S01 | 0 | 2 | 14  E | 35  E | 12 | 3 | 4 | 3 | 8 | 6 | 10 | 6 |  | E |  |  |
| **Metalloproteases** |  |  |  |  |  |  |  |  |  |  |  |  |  |  |  |  |
| M10 | 0.021 | 1 | 0 | 3  E | 0 | 0 | 2 | 0 | 5 | 4 | 2 | 1 |  |  |  |  |
| M43 | 0 | 7 | 5  E | 17  E | 8 | 3 | 1 | 1 | 5 | 2 | 2 | 3 |  |  |  |  |
| M16 | 0 | 0 | 0 | 0 | 0 | 0 | 0 | 0 | 0 | 0 | 1  E | 0 |  |  |  |  |
| **Aspartic proteases** | |  |  |  |  |  |  |  |  |  |  |  |  |  |  |  |
| A11 | 0 | 8  E | 0 | 11  E | 0 | 0 | 0 | 1 | 1 | 1 | 13  E | 0  C |  |  |  |  |
| **Carboxypeptidases** | |  |  |  |  |  |  |  |  |  |  |  |  |  |  |  |
| C02 | 0 | 3 | 1 | 1 | 2 | 3 | 4 | 3 | 6 | 3 | 15  E | 4 |  |  |  |  |
|  |  |  |  |  |  |  |  |  |  |  |  |  |  |  |  |  |
| **Cytochrome P450s** | |  |  |  |  |  |  |  |  |  |  |  |  |  |  |  |
| All p450s (Nelsons p450 Database) | 0 | 71  C | 101  E | 127  E | 62  C | 74 | 129  E | 78  C | 177  E | 127 | 186  E | 141  E |  |  |  |  |
| **P450 Families** |  |  |  |  |  |  |  |  |  |  |  |  |  |  |  |  |
| CYP567 | 0.012 | 1 | 0 | 3  E | 3 | 0 | 0 | 0 | 1 | 0 | 3 | 2 |  |  |  |  |
| CYP634 | 0.016 | 0 | 0 | 0 | 0 | 0 | 1 | 0 | 1 | 1 | 5  E | 1 |  |  |  |  |
| CYP5133 | 0.013 | 0 | 0 | 0 | 0 | 0 | 0 | 0 | 0 | 0 | 4  E | 0 |  |  |  |  |
| CYP548 | 0.004 | 3 | 7 | 6 | 1 | 3 | 2 | 2 | 2 | 2 | 8  E | 3 |  |  |  |  |
| CYP6002 | 0.038 | 0 | 1 | 0 | 0 | 0 | 0 | 0 | 0 | 0 | 2  E | 0 |  |  |  |  |
| CYP682 | 0.015 | 2 | 0 | 3  E | 3  E | 1 | 0 | 0 | 0 | 0 | 1 | 0 |  |  |  |  |
| **CAZymes** |  |  |  |  |  |  |  |  |  |  |  |  |  |  |  |  |
| Carbohydrate Binding Modules (CBM) | 0 | 18 | 22 | 26 | 21 | 21 | 20 | 17 | 44 | 41 | 45 | 40 | E |  |  |  |
| **Carbohydrate Esterases (CE)** | 0 | 35  C | 29  C | 39 | 38 | 54 | 64 | 41 | 93 | 87 | 106  E | 98  E | E |  |  |  |
| Glycoside Hydrolases (GH) | 0 | 165  C | 184  C | 213  C | 187 | 287 | 323  E | 223  C | 390  E | 296  E | 418  E | 340  E | E | C |  | C |
| Glycosyl Transferases (GT) | 0 | 117 | 122 | 139 | 114 | 123 | 131 | 112 | 161  E | 130 | 160  E | 138  C |  |  |  |  |
| Polysaccharide Lyases (PL) | 0 | 2 | 2 | 3 | 3 | 8 | 6 | 5 | 35  E | 20 | 23 | 21 | E |  |  |  |
| **Carbohydrate Binding Modules(CBM)** | | |  |  |  |  |  |  |  |  |  |  |  |  |  |  |
| CBM21 | 0 | 1 | 1 | 1 | 1 | 1 | 1 | 1 | 2 | 1 | 9  E | 1 |  |  |  |  |
| **Carbohydrate Esterases (CE)** | |  |  |  |  |  |  |  |  |  |  |  |  |  |  |  |
| CE1 | 0.021 | 3 | 3 | 2 | 3 | 5 | 7 | 5 | 3 | 8 | 10  E | 5 |  |  |  |  |
| CE10 | 0 | 16 | 9  C | 17 | 15 | 22 | 30 | 16 | 45 | 35 | 45 | 40 | E |  |  |  |
| CE3 | 0.033 | 2 | 1 | 1 | 1 | 3 | 2 | 1 | 7 | 3 | 4 | 8  E |  |  |  |  |
| **Glycoside Hydrolases (GH)** | |  |  |  |  |  |  |  |  |  |  |  |  |  |  |  |
| GH18/CBM18 | 0.01 | 1 | 2 | 4 | 5 | 6 | 4 | 0  C | 8 | 4 | 7 | 5 |  |  |  |  |
| GH18/CBM18/CBM50 | 0.036 | 4 | 1  C | 5 | 4 | 2 | 8 | 5 | 5 | 5 | 4 | 5 |  |  |  |  |
| GH28 | 0.002 | 4 | 3 | 3 | 4 | 9 | 12 | 6 | 18  E | 9 | 18  E | 12 |  |  |  |  |
| GH3 | 0 | 9 | 7 | 9 | 10 | 15 | 19 | 13 | 42  E | 23 | 35  E | 28 | E |  |  |  |
| GH32 | 0.03 | 2 | 2 | 1 | 2 | 3 | 3 | 1 | 5 | 5 | 10  E | 6 |  |  |  |  |
| GH35 | 0.039 | 0 | 0 | 0 | 0 | 0 | 0 | 0 | 1 | 0 | 4 | 1 |  |  |  |  |
| GH38 | 0.041 | 1 | 1 | 5  E | 1 | 1 | 2 | 1 | 1 | 1 | 2 | 1 |  |  |  |  |
| GH4 | 0.002 | 16 | 18 | 24  E | 15 | 17 | 28 | 12 | 11 | 21 | 13 | 12 |  |  |  |  |
| GH43 | 0 | 1  C | 1  C | 1  C | 3 | 12 | 10 | 6 | 36  E | 17 | 31  E | 22 | E | C |  |  |
| GH76 | 0.034 | 7 | 15 | 14 | 12 | 11 | 12 | 13 | 13 | 12 | 20  E | 13 | E | E |  |  |
| GH78 | 0 | 2 | 1 | 1 | 1 | 4 | 4 | 2 | 13 | 8 | 17  E | 10 |  |  |  |  |
| GH79 | 0.003 | 2 | 1 | 2 | 1 | 4 | 4 | 4 | 1 | 0 | 6  E | 2 |  |  |  |  |
| **Glycosyl Transferases (GT)** | |  |  |  |  |  |  |  |  |  |  |  |  |  |  |  |
| GT1 | 0.008 | 10 | 13 | 14 | 13 | 9 | 10 | 9 | 22 | 18 | 22  E | 14  C |  |  |  |  |
| GT34 | 0.021 | 8 | 7 | 10 | 5 | 8 | 11 | 5 | 18  E | 11 | 12 | 9 |  |  |  |  |
| **Polysaccharide Lyases (PL)** | | | | | | | | | | | | | | | | |
| PL1 | 0.007 | 0 | 0 | 0 | 0 | 2 | 0 | 0 | 13  E | 9 | 11 | 11 |  |  |  |  |

Gene families (column 1) with family-wide p-values < .05 (column 2) and numbers in individual species (columns 3-13) or individual nodes from Hypocrealean phylogeny in Figure 3A (columns 14-17) significantly expanded (E/shaded grey) or contracted (C/hatched) at p-value <0.01.
