# Supplementary material for: The Genome of Tolypocladium inflatum: Evolution, Organization, and Expression of the Cyclosporin Biosynthetic Gene Cluster
Source: PLoS Genet. 2013 Jun 20;9(6):e1003496. doi: 10.1371/journal.pgen.1003496 (PMC3688495; doi:10.1371/journal.pgen.1003496)
Supplement: Table S3 — Table of identified T. inflatum core secondary metabolites including NRPSs, PKSs, NRPS-like, PKS-like, and DMAT enzymes. (DOCX) [file pgen.1003496.s011.docx]

**Table S3| Secondary metabolite core genes in *T. inflatum***

| **Gene ID** | **Contig** | **SMURF** | **anti- SMASH** | **Custom**  **HMMER** | **Domain Structure** | **Clade/Known Homologs** | **Chemical Product if Known** |
| --- | --- | --- | --- | --- | --- | --- | --- |
| **NRPS** | | | | | | | |
| TINF00065 | 3 |  |  | X | A-T | ChNPS11/Gliotoxin |  |
| TINF00159 | 4 | X | X | X | A-T-C-A-M-T-C-A-M-T-C-A-M-T-C-A-M-T-C-A-T-C-A-M-T-C-A-M-T-C-A-T-C-A-M-T-C-A-T-C | Cyclosporin | Cyclosporin |
| TINF01764 | 30 | X | X | X | A-T-C-dA-T-C | NPS6 |  |
| TINF02556 | 35 | X | X | X | A-T-C-A-T-C-A-T-C-A-T-C | Ergot Alkaloids |  |
| TINF03094 | 37 | X | X | X | A-T-C-A-T-NAD | NPS4/PesA |  |
| TINF03193 | 37 | X | X | X | A-T-C |  |  |
| TINF04771 | 41 | X | X | X | A-T-C-A-T-C-A-T-NAD | NPS8/PerA |  |
| TINF05969 | 47 | X | X | X | T-C-(A-T-C)x11 | Peptaibol |  |
| TINF06175 | 47 | X | X | X | A-T-C-dA-T-C | NPS6 |  |
| TINF07827 | 55 | X | X | X | (ATC)x13-T-C | Peptaibol |  |
| TINF07876 | 55 | X | X | X | KS-(A-T-C-)x8 | Peptaibol |  |
| TINF08996 | 70 | X | X | X | A-T-C-A-T-C-T-C-A-T-C-T-C-T-C | NPS2 | Ferrichrome |
| TINF09441 | 73 | X | X | X | A-T-C-A-T-E-C-A-T | NPS8/PerA |  |
| TINF09615 | 78 | X |  | X | C-T-A-T-C |  |  |
|  |  |  |  |  |  |  |  |
| **NRPS-like** | | | | | | | |
| TINF09755 | 88 | X |  | X | A-T-NAD-Adh | ChNPS10 |  |
| TINF06128 | 47 |  |  | X | A | Ingroup/ChNPS12_1 |  |
| TINF05741 | 47 |  |  | X | A | Ingroup/ChNPS12_2 |  |
| TINF02624 | 35 | X | X |  | A-T-NAD | Ochratoxin |  |
| TINF02476 | 35 |  | X |  | A |  |  |
| TINF04438 | 41 | X | X |  | A-T |  |  |
| TINF08209 | 62 | X |  |  | A-T-NAD |  |  |
| TINF08448 | 66 |  | X | X | A-T-NAD | AAR |  |
| TINF09066 | 70 |  | X |  | A |  |  |
| TINF08599 | 69 |  | X |  | A-TR | Ochratoxin |  |
| TINF05358 | 43 | X |  |  | C-A-T-KS | NPS-like |  |
| **NPS-PKS Hybrid** | | | | | | | |
| TINF01692 | 30 | X |  | X | KS-MT-KR-D-T-A-T-C-T-C |  |  |
| TINF04403 | 41 | X | X | X | T-MT-KS-D-KR-T-C-A-T-NAD |  |  |
| TINF07495 | 51 | X | X | X | KS-MT-D-KR-T-C-A-T-NAD |  |  |
| TINF07675 | 51 | X | X | X | KS-MT-D-KR-T-C-A-T-NAD-MT |  |  |
| **PKS** | | | | | | | |
| TINF00235 | 4 | X | X | X | KS-D-KR-T |  |  |
| TINF00267 | 4 | X | X | X | KS-MT-KR-T |  |  |
| TINF01724 | 30 | X | X | X | KS-MT-KR-T-A-C-T-C-A-T-C- |  |  |
| TINF02578 | 35 | X | X | X | KS-MT-KR-T |  |  |
| TINF02842 | 35 | X | X | X | KS-KR |  |  |
| TINF03960 | 39 | X | X | X | KS-T-MT |  |  |
| TINF03867 | 39 | X | X | X | KS-T-KR-NAD |  |  |
| TINF05238 | 43 | X | X | X | KS-T-MT-NAD |  |  |
| TINF05098 | 43 | X | X | X | KS-T-MT |  |  |
| TINF06105 | 47 | X | X | X | KS-MT-KR-T |  |  |
| TINF07720 | 54 | X | X | X | KS-MT-KR |  |  |
| TINF07778 | 54 | X | X | X | KS-KR-T |  |  |
| TINF07706 | 54 | X | X | X | KS |  |  |
| TINF07709 | 54 | X |  | X | KS-KR-T |  |  |
| TINF08217 | 62 | X | X | X | KS-TE |  |  |
| TINF08809 | 70 | X | X | X | KS-MT-D-KR-T |  |  |
| TINF08990 | 70 | X | X | X | KS-KR-T |  |  |
| TINF09333 | 70 | X | X | X | KS-KR-MT-KR-T |  |  |
| TINF02226 |  |  |  | X | KS-KR-T |  |  |
| TINF09890 |  |  |  | X | KS-T-MT |  |  |
| PKS-like | | | | | | | |
| TINF09694 | 82 | X | X | X | KS |  |  |
| TINF00970 |  |  |  | X | KS-KR |  |  |
| TINF06035 |  |  |  | X | KS-MT |  |  |
| TIN06787 | 50 | X |  |  | No domains detected in HMMER searches |  |  |
| TINF09888 | 92 | X |  |  | No domains detected in HMMER searches |  |  |
| **DMAT synthetase** | | | | | | | |
| TINF04722 | 41 | X | X |  |  |  |  |

Secondary metabolite genes in *T. inflatum* identified by one of three methods: SMURF analysis pipeline (column 3), antiSMASH analysis pipeline (column 4), and custom pipeline using hmmer searches (column 5).
