# Supplementary material for: The Genome of Tolypocladium inflatum: Evolution, Organization, and Expression of the Cyclosporin Biosynthetic Gene Cluster
Source: PLoS Genet. 2013 Jun 20;9(6):e1003496. doi: 10.1371/journal.pgen.1003496 (PMC3688495; doi:10.1371/journal.pgen.1003496)
Supplement: Table S4 — NRPSs in T. inflatum and other hypocrealean taxa with A-domains that group with known NRPSs (top row) in larger phylogeny (Figure S3). The number of modules (M) present in each NRPS is listed after each gene. (DOCX) [file pgen.1003496.s012.docx]

| **Table S4\| Homologs of known NRPS synthetases in *T. inflatum* and other hypocrealean taxa.** | | | | | | | | |  |  |  |
| --- | --- | --- | --- | --- | --- | --- | --- | --- | --- | --- | --- |
|  | Ergot Alkaloids | Peptaibols | *simA* clade | *perA-*like Clade | Ch NPS2 | Ch NPS6 | Ch NPS8 | Ch NPS10 |  |  |  |
| **Characterized Fungal NRPS genes in each clade** | ***cpps2,cpps3* (1M) *cpps1/cpps4* (3M) (*Claviceps purpurea*)** | ***tex1 (*18M)** | ***simA (Tolypocladium inflatum) (*11M)**  ***dtxA (Metarhizium robertsii) (6M)***  ***esyn1 (Fusarium equiseti) (2M)*** | ***perA (*2M)**  ***(Epichoë festucae)*** | ***Ch NPS2 (4M)***  **(*Cochliobolus heterostrophus*)** | ***Ch NPS6 (1M)***  **(*Cochliobolus heterostrophus*)** | ***Ch NPS8 (2M)***  **(*Cochliobolus heterostrophus*)** | ***Ch NPS10 (1M)***  **(*Cochliobolus heterostrophus*)**  ***MAA* (1M)**  **(*Leptosphaeria maculans*)** |  |  |  |
| ***T. inflatum*** | TINF02556 (4M) | TINF05969 (11M)  TINF07827 (13M)  TINF07876 (8 M) | TINF00159 (11M) = *simA* | TINF04771 (3M) | TINF08996 (3M) | TINF01764 (1M)  TINF06175 (1M) | TINF09441 (3M)  TINF04771 (3M) | TINF09755 (1M) |  |  |  |
| ***F. graminearum*** | FGSG11395 (2M)  FGSG11319 (Partial A domain) | None | FG11989 (1M) | none | FGSG05372 (3M)  FGSG11026 (1M) | FGSG03747(1M) | FGSG02394 (2M) | FGSG06507 (1M) |  |  |  |
| ***F. oxysporum*** | FOXG04898 (2M) | none | FOXG11847 (2M) | none | FOXG06448 (3M)  FOXG17422 (3M)  FOXG17714 (1M) | FOXG09785 (1M) | none | FOXG02458 (1M) |  |  |  |
| ***F. verticillioides*** | none | none | FVEG09993 (2M) | FVEG06502 (1M)  FVEG06496(2M) | FVEG12503 (3M)  FVEG04296 (2M) | FVEG08697 (1M) | none | FVEG05643 (1M) |  |  |  |
| ***N. haematococca*** | none | none | Nh106280 (5M)- M5 of 5 groups with *simA* | Nh10628 (5M) –  M1-4 of 5 groups with *perA* | Nh103597 (3M)  Nh86967 (3M) | Nh95699 (1M)  Nh53641 (1M) | none | Nh68854 (1M) |  |  |  |
| ***C. militaris*** | none | none | CM07302 (1M) | none | CCM00722 (1M) CCM01705 (3M)  CCM04725 (1M)  CCM09341 (1M) | CCM06702 (1M) | CCM03255 (4M) | CCM02691 (1M) |  |  |  |
| ***M. robertsii*** | MAA06742 (1M)=*cpps2*  MAA06744 (1M)=*cpps3*  MAA06559 (2M)  MAA09689 (7M) | none | MAA10043 (6M = *dtxA* - M5-6 of 6 group with *simA* clade  MAA996513 (1M) –M1 of 2 w/ESYN M2 | MAA09844 (3)  MAA10043 (6M) =*dtxA -* M1-4 of 6 group w/*perA* | MAA01890 (3M) | MAA05334 (1M) | MAA10410 (2M)  MAA09772 (2M)  MAA09844 (3M)  MAA09953 (8M)  MAA10410 (1M) | MAA00714 (1M) |  |  |  |
| ***M. acridum*** | MAC0698 (1M)=*cpps2*  MAC06980 (1M)=*cpps3*  MAC08899 (7M) | none | MAC05180 (1M) - M1 of 2 w/*esyn* M2 | MAC95387 (3M)  MAC01861 (M8) | MAC07977 (3M) | MAC01861 (1M) | MAC02762 (2M)  MAC04406 (4M)  MAC05398 (3M) | MAC06316 (1M) |  |  |  |
| ***Tr. reeseii*** | none | Tr123786 (14M)  Tr23171 (18M) | Tr60751 (1M) -M1 of 2 w/ESYN M2 | none | Tr69946 (3M) | Tr67189 (1M)  Tr71005 (1M) | none | none |  |  |  |
| ***Tr. atroviride*** | none | Ta317938 (19M)  Ta10579 (1M)  Ta47989 (1M)  Ta48550 (1M)  Ta39904 (2M)  Ta46079 (1M) | Ta30015 (2M) | none | Ta31890 (3M) | Ta39887 (1M) | Ta211347 (5M) | Ta228361 (1M) |  |  |  |
| ***Tr. virens*** | none | Tv66940 (18M)  Tv10003 (14M)  Tv69362 (8M) | Tv46961 (2M) | Tv52861(5M) | Tv85582 (3M) | Tv44273 (1M) | Tv52861 (5M) | Tv39004 (1M) |  |  |  |

Note: For each gene, M refers to the number of A-T-C modules found in the NRPS. In some cases (E.g. MAA10043, MAA996513 ) some modules group with one known NRPS while other modules from the same gene group with another known fungal NRPS.
