# Supplementary material for: The Genome of Tolypocladium inflatum: Evolution, Organization, and Expression of the Cyclosporin Biosynthetic Gene Cluster
Source: PLoS Genet. 2013 Jun 20;9(6):e1003496. doi: 10.1371/journal.pgen.1003496 (PMC3688495; doi:10.1371/journal.pgen.1003496)
Supplement: Table S5 — Table of q-values, fold change, and RPKM values at six time points (days 2, 4, 6, 8, 10, 12) in the cyclosporin time course experiment. Genes in the RNA-Seq defined cluster are shaded green. (DOCX) [file pgen.1003496.s013.docx]

| **Table S5\| Q-values, log_2_ fold change, and relative expression levels of genes in and surrounding the *simA* cluster under inducing (SM) and control (SDB) conditions.** | | | | | | | |
| --- | --- | --- | --- | --- | --- | --- | --- |
| 1. Q-values for comparisons of SM to SDB at each time point with values < 0.005 indicated with an *. Genes in the RNA-Seq defined cluster are shaded green. | | | | | | | |
| gene | t1-q-value | t2-q-value | | t3-q-value | t4-q-value | t5-q-value | t6-q-value |
| TINF00496 | 5.49E-01 | 1.00E+00 | | 9.61E-02 | 5.66E-01 | 2.23E-01 | 7.91E-01 |
| TINF00268 | 2.51E-01 | 1.00E+00 | | 3.11E-01 | 7.01E-01 | 4.90E-01 | 7.99E-01 |
| TINF00352 | 2.58E-02 | 1.00E+00 | | *9.97E-13 | 5.00E-01 | 1.20E-02 | 5.53E-01 |
| TINF00373 | 5.83E-01 | 1.00E+00 | | 2.57E-01 | 8.06E-01 | 2.89E-02 | 7.70E-01 |
| TINF00467 | 6.10E-01 | 1.00E+00 | | 7.26E-03 | 7.37E-01 | 3.88E-02 | 8.22E-01 |
| TINF00459 | 4.88E-01 | 1.00E+00 | | 5.11E-02 | 7.45E-01 | 7.05E-01 | 8.27E-01 |
| TINF00234 | 3.31E-01 | 1.00E+00 | | 3.21E-01 | 7.30E-01 | 2.73E-01 | 8.18E-01 |
| TINF00233 | 5.53E-01 | 1.00E+00 | | *4.80E-06 | 4.34E-01 | 7.04E-01 | 5.62E-01 |
| TINF00513 | 7.09E-01 | 1.00E+00 | | 2.22E-01 | 6.05E-01 | 2.86E-01 | 7.96E-01 |
| TINF00291 | 5.61E-01 | 1.00E+00 | | 3.28E-02 | 5.58E-01 | 1.22E-01 | 7.51E-01 |
| TINF00177 | 6.49E-01 | 1.00E+00 | | 2.26E-01 | 6.75E-01 | 5.26E-01 | 9.03E-01 |
| TINF00355 | 6.95E-01 | 1.00E+00 | | 3.91E-02 | 7.97E-01 | 6.80E-01 | 8.74E-01 |
| TINF00502 | 3.73E-01 | 1.00E+00 | | 3.71E-01 | 7.94E-01 | 5.02E-01 | 7.97E-01 |
| TINF00596 | 6.13E-01 | 1.00E+00 | | 3.28E-02 | 7.96E-01 | 5.99E-01 | 8.53E-01 |
| TINF00408 | 2.54E-01 | 1.00E+00 | | *4.09E-03 | 3.48E-01 | 7.23E-02 | 7.46E-01 |
| TINF00464 | 3.84E-01 | 1.00E+00 | | 2.66E-01 | 3.84E-01 | 1.54E-01 | 7.04E-01 |
| TINF00183 | 5.31E-01 | 1.00E+00 | | *1.08E-03 | 6.15E-01 | 3.79E-01 | 8.78E-01 |
| TINF00557 | 3.28E-01 | 1.00E+00 | | 1.35E-01 | 7.30E-01 | 2.64E-01 | 8.78E-01 |
| TINF00159 | 2.62E-02 | 1.00E+00 | | *4.58E-125 | *2.12E-29 | *1.18E-50 | *1.07E-23 |
| TINF00247 | 6.43E-01 | 1.00E+00 | | *1.09E-145 | *2.37E-32 | *7.39E-59 | *2.75E-28 |
| TINF00586 | 2.15E-01 | 1.00E+00 | | *1.45E-29 | *1.57E-04 | *2.99E-14 | *1.60E-05 |
| TINF00536 | 7.21E-01 | 1.00E+00 | | *2.40E-14 | 2.81E-02 | *2.24E-07 | *6.13E-02 |
| TINF00426 | 6.46E-01 | 1.00E+00 | | *7.33E-15 | *1.75E-04 | *3.32E-11 | *2.75E-04 |
| TINF00174 | 5.18E-01 | 1.00E+00 | | *7.43E-101 | *7.60E-24 | *3.91E-44 | *3.92E-24 |
| TINF00267 | 1.67E-01 | 1.00E+00 | | *5.78E-188 | *3.42E-38 | *5.48E-72 | *7.37E-33 |
| TINF00377 | 5.42E-01 | 1.00E+00 | | *8.13E-157 | *1.16E-39 | *1.13E-68 | *6.87E-36 |
| TINF00470 | 9.77E-02 | 1.00E+00 | | *1.92E-192 | *8.11E-39 | *3.93E-72 | *2.02E-34 |
| TINF00351 | 3.26E-01 | 1.00E+00 | | *7.60E-196 | *2.67E-34 | *4.40E-71 | *2.77E-36 |
| TINF00195 | 0.00E+00 | 1.00E+00 | | *9.96E-88 | *2.02E-39 | *1.04E-50 | *5.76E-30 |
| TINF00141 | 0.00E+00 | 1.00E+00 | | *7.45E-54 | *4.59E-32 | *2.38E-39 | *1.77E-22 |
| TINF00394 | *1.36E-05 | 1.00E+00 | | *9.06E-93 | *5.10E-20 | *3.36E-42 | *7.06E-14 |
| TINF07874 | 0.00E+00 | 1.00E+00 | | *3.14E-69 | *2.41E-14 | *3.73E-31 | *2.37E-18 |
| TINF00620 | 0.00E+00 | 1.00E+00 | | 0.00E+00 | 0.00E+00 | 0.00E+00 | 0.00E+00 |
| TINF00605 | 4.86E-01 | 1.00E+00 | | *2.93E-05 | 8.06E-01 | 6.20E-01 | 9.03E-01 |
| TINF00458 | 5.98E-01 | 1.00E+00 | | 2.42E-01 | 4.73E-01 | 4.51E-01 | 9.03E-01 |
| TINF00432 | 3.97E-01 | 1.00E+00 | | 1.68E-02 | 3.65E-01 | 4.99E-01 | 8.52E-01 |
| TINF00266 | 5.31E-01 | 1.00E+00 | | 2.28E-02 | 5.86E-01 | 3.15E-02 | 8.08E-01 |
| TINF00548 | 3.10E-01 | 1.00E+00 | | 8.16E-02 | 8.00E-01 | 7.02E-01 | 8.39E-01 |
| TINF00185 | 0.00E+00 | 1.00E+00 | | 0.00E+00 | 0.00E+00 | 0.00E+00 | 0.00E+00 |
| TINF00554 | 0.00E+00 | 1.00E+00 | | 1.78E-01 | 7.74E-01 | 4.75E-01 | 9.03E-01 |
| TINF00588 | 0.00E+00 | 1.00E+00 | | *1.54E-04 | 1.85E-01 | 2.09E-02 | 7.80E-02 |
| TINF00492 | 1.57E-01 | 1.00E+00 | | 8.72E-02 | 7.75E-01 | 5.53E-02 | 1.50E-01 |
|  |  |  | |  |  |  |  |
| 1. Log_2_ transformed fold change of expression levels from control SDB to inducing SM media – measured as mean normalized count in SM/mean normalized count in SDB | | | | | | | |
| gene | t1 log_2_ foldchange | | t2 log_2_ foldchange | t3 log_2_ foldchange | t4 log_2_ foldchange | t5 log_2_ foldchange | t6 log_2_ foldchange |
| TINF00496 | 0.3267 | | 0.1074 | 0.3266 | -0.4898 | -0.5798 | -0.4898 |
| TINF00268 | 0.7426 | | 0.2423 | 0.1457 | -0.3201 | -0.3766 | -0.3201 |
| TINF00352 | -1.0467 | | -0.2564 | -1.5438 | -0.5723 | -1.1098 | -0.5723 |
| TINF00373 | -0.2980 | | -0.1338 | -0.2269 | -0.0038 | -1.1442 | -0.0038 |
| TINF00467 | -0.1962 | | 0.0205 | -0.6258 | -0.2071 | -0.9726 | -0.2071 |
| TINF00459 | 0.4078 | | 0.0276 | 0.4094 | 0.1945 | -0.0302 | 0.1945 |
| TINF00234 | 0.5726 | | 0.0605 | 0.0779 | 0.2292 | 0.5112 | 0.2292 |
| TINF00233 | 0.2739 | | 0.5874 | 1.3386 | -0.5490 | -0.0109 | -0.5490 |
| TINF00513 | -0.0348 | | -0.2184 | -0.3014 | -0.3132 | -0.7425 | -0.3132 |
| TINF00291 | -0.4386 | | 0.0539 | -0.6670 | -0.5040 | -0.8342 | -0.5040 |
| TINF00177 | 0.1890 | | 0.5406 | 0.2788 | 0.2147 | 0.3642 | 0.2147 |
| TINF00355 | 0.1218 | | -0.0218 | 0.4688 | 0.0699 | 0.0399 | 0.0699 |
| TINF00502 | 0.5850 | | 0.0272 | -0.0401 | -0.0998 | -0.2554 | -0.0998 |
| TINF00596 | 0.2255 | | -0.1214 | 0.5176 | -0.0698 | -0.1560 | -0.0698 |
| TINF00408 | 0.6829 | | -0.3539 | -0.6968 | -0.7186 | -0.8713 | -0.7186 |
| TINF00464 | 0.5910 | | -0.2733 | 0.1584 | 0.7060 | 0.7298 | 0.7060 |
| TINF00183 | 0.3167 | | 0.0682 | 0.8041 | 0.4088 | 0.3620 | 0.4088 |
| TINF00557 | 0.5758 | | 0.2775 | 0.2909 | -0.2083 | 0.5023 | -0.2083 |
| TINF00159 | 1.5042 | | 0.2630 | 7.2084 | 7.3965 | 7.6902 | 7.3965 |
| TINF00247 | 0.3440 | | -0.8885 | 8.7613 | 7.9145 | 9.0069 | 7.9145 |
| TINF00586 | 0.7211 | | 0.3207 | 2.4812 | 2.2343 | 3.1814 | 2.2343 |
| TINF00536 | 0.0584 | | -0.0808 | 1.8228 | 1.3697 | 2.2402 | 1.3697 |
| TINF00426 | 0.1570 | | -0.3291 | 1.8959 | 2.1560 | 2.8597 | 2.1560 |
| TINF00174 | 0.7655 | | -0.6067 | 7.9259 | 6.3872 | 7.7279 | 6.3872 |
| TINF00267 | 1.2451 | | -0.8742 | 9.5906 | 9.1897 | 10.1150 | 9.1897 |
| TINF00377 | 0.3479 | | -1.5759 | 9.6222 | 9.7095 | 10.5362 | 9.7095 |
| TINF00470 | 1.9143 | | -0.6000 | 10.2450 | 9.3450 | 10.5042 | 9.3450 |
| TINF00351 | 1.2479 | | -0.6049 | 10.0393 | 8.4714 | 10.1792 | 8.4714 |
| TINF00195 | 0.0000 | | -1.3440 | 10.8788 | 12.0126 | 9.6257 | 12.0126 |
| TINF00141 | 0.0000 | | -0.3785 | 7.8580 | 8.8685 | 8.1708 | 8.8685 |
| TINF00394 | 2.5673 | | 0.6137 | 5.8690 | 5.4234 | 6.8839 | 5.4234 |
| TINF07874 | 0.0000 | | -0.2824 | 8.2538 | 4.8503 | 6.6360 | 4.8503 |
| TINF00620 | 0.0000 | | 0.0000 | 0.0000 | 0.0000 | 0.0000 | 0.0000 |
| TINF00605 | -2.4594 | | 0.5305 | 2.3219 | 0.0544 | 0.2224 | 0.0544 |
| TINF00458 | -0.1926 | | -0.1699 | 1.0875 | -1.7004 | 1.1375 | -1.7004 |
| TINF00432 | 0.4636 | | 0.1656 | 0.5911 | 0.7148 | 0.2439 | 0.7148 |
| TINF00266 | -0.3589 | | -0.1177 | -0.5234 | -0.4661 | -1.0151 | -0.4661 |
| TINF00548 | 0.6321 | | -0.2197 | 0.3694 | -0.0431 | -0.0285 | -0.0431 |
| TINF00185 | 0.0000 | | 0.0000 | 0.0000 | 0.0000 | 0.0000 | -0.0431 |
| TINF00554 | 0.0000 | | 0.0000 | 1.5850 | 0.6781 | -1.2630 | 0.6781 |
| TINF00588 | 0.0000 | | 0.0000 | 2.1926 | 1.2035 | 1.2602 | 1.2035 |
| TINF00492 | 1.0297 | | 0.8278 | 0.4705 | 0.2436 | 1.0649 | 0.2436 |
|  |  | |  |  |  |  |  |
| 1. Relative expression levels in SDB media at each time point – average RPKM across 3 biological replicates | | | | | | | |
| gene | t1-SDB_RPKM | t2-SDBRPKM | | t3-SDB_RPKM | t4-SDB_RPKM | t5-SDB_RPKM | t6-SDB_RPKM |
| TINF00496 | 25.07 | 30.16 | | 33.03 | 42.20 | 46.97 | 42.61 |
| TINF00268 | 11.00 | 12.95 | | 13.15 | 10.05 | 12.26 | 15.38 |
| TINF00352 | 1977.68 | 1785.00 | | 1609.68 | 1571.18 | 1798.28 | 960.52 |
| TINF00373 | 6.26 | 6.93 | | 7.29 | 10.29 | 10.67 | 9.19 |
| TINF00467 | 28.49 | 27.25 | | 32.39 | 39.82 | 39.77 | 33.50 |
| TINF00459 | 30.38 | 31.74 | | 31.05 | 29.45 | 31.17 | 29.89 |
| TINF00234 | 1676.67 | 1405.42 | | 996.13 | 695.72 | 712.67 | 676.92 |
| TINF00233 | 2.14 | 2.97 | | 3.44 | 6.28 | 4.53 | 7.52 |
| TINF00513 | 6.24 | 7.38 | | 8.04 | 5.43 | 5.39 | 6.62 |
| TINF00291 | 5.40 | 4.53 | | 8.84 | 14.65 | 16.54 | 18.06 |
| TINF00177 | 1.38 | 2.51 | | 4.67 | 3.72 | 4.91 | 4.33 |
| TINF00355 | 47.54 | 53.48 | | 60.02 | 81.28 | 81.73 | 99.46 |
| TINF00502 | 7.95 | 8.79 | | 8.62 | 9.48 | 10.16 | 12.21 |
| TINF00596 | 69.14 | 63.75 | | 61.94 | 92.55 | 104.06 | 111.38 |
| TINF00408 | 115.80 | 93.38 | | 62.95 | 55.24 | 60.78 | 62.60 |
| TINF00464 | 31.62 | 32.33 | | 29.22 | 20.57 | 21.56 | 25.65 |
| TINF00183 | 27.13 | 29.38 | | 31.18 | 24.64 | 28.67 | 36.94 |
| TINF00557 | 47.06 | 55.82 | | 47.24 | 26.39 | 28.76 | 26.63 |
| TINF00159 | 0.04 | 0.52 | | 0.08 | 0.11 | 0.09 | 0.11 |
| TINF00247 | 0.91 | 18.20 | | 0.98 | 1.17 | 0.95 | 1.04 |
| TINF00586 | 229.99 | 614.84 | | 427.54 | 290.91 | 393.57 | 247.57 |
| TINF00536 | 19.08 | 20.06 | | 16.86 | 11.95 | 12.77 | 11.95 |
| TINF00426 | 78.58 | 79.91 | | 62.83 | 42.92 | 45.73 | 49.21 |
| TINF00174 | 0.55 | 6.09 | | 0.48 | 0.63 | 0.61 | 0.38 |
| TINF00267 | 0.13 | 7.06 | | 0.20 | 0.22 | 0.16 | 0.18 |
| TINF00377 | 0.42 | 20.97 | | 0.58 | 0.57 | 0.46 | 0.66 |
| TINF00470 | 0.51 | 34.35 | | 0.64 | 0.72 | 0.53 | 0.72 |
| TINF00351 | 0.83 | 49.66 | | 0.89 | 1.31 | 0.82 | 0.93 |
| TINF00195 | 0.00 | 10.83 | | 0.16 | 0.16 | 0.62 | 0.48 |
| TINF00141 | 0.00 | 0.37 | | 0.04 | 0.07 | 0.08 | 0.03 |
| TINF00394 | 1.59 | 32.01 | | 6.02 | 2.80 | 2.52 | 2.37 |
| TINF07874 | 0.00 | 1.06 | | 0.05 | 0.15 | 0.15 | 0.06 |
| TINF00620 | 0.00 | 0.00 | | 0.00 | 0.00 | 0.00 | 0.00 |
| TINF00605 | 0.15 | 0.12 | | 0.18 | 0.42 | 0.38 | 0.42 |
| TINF00458 | 0.18 | 0.16 | | 0.16 | 0.22 | 0.08 | 0.17 |
| TINF00432 | 9.37 | 10.43 | | 9.78 | 9.31 | 9.70 | 12.65 |
| TINF00266 | 71.69 | 92.79 | | 83.49 | 130.67 | 100.72 | 87.58 |
| TINF00548 | 15.97 | 17.97 | | 15.65 | 16.58 | 16.05 | 20.08 |
| TINF00185 | 0.00 | 0.00 | | 0.00 | 0.00 | 0.00 | 0.00 |
| TINF00554 | 0.00 | 0.00 | | 0.05 | 0.11 | 0.28 | 0.31 |
| TINF00588 | 0.00 | 0.00 | | 0.88 | 3.95 | 5.38 | 5.67 |
| TINF00492 | 1.14 | 3.15 | | 6.30 | 3.63 | 3.73 | 3.13 |
| 1. Relative expression levels in SM media at each time point – Average RPKM across 3 biological replicates | | | | | | | |
|  | t1_SMRPKM | t2-SMRPKM | | t3-SM_RPKM | t4-SM_RPKM | t5-SM_RPKM | t6-SM_RPKM |
| TINF00496 | 30.45 | 32.95 | | 42.27 | 29.84 | 31.49 | 32.36 |
| TINF00268 | 19.03 | 15.53 | | 14.43 | 8.12 | 9.77 | 12.21 |
| TINF00352 | 918.12 | 1557.32 | | 559.28 | 1063.86 | 815.32 | 547.80 |
| TINF00373 | 5.24 | 6.41 | | 6.33 | 10.09 | 4.85 | 6.65 |
| TINF00467 | 24.86 | 28.29 | | 21.32 | 33.89 | 20.31 | 28.88 |
| TINF00459 | 40.09 | 32.71 | | 41.62 | 33.62 | 30.90 | 26.34 |
| TINF00234 | 2560.68 | 1474.19 | | 1049.14 | 848.08 | 1023.85 | 828.50 |
| TINF00233 | 2.54 | 4.54 | | 8.76 | 4.23 | 4.54 | 4.64 |
| TINF00513 | 5.63 | 6.40 | | 6.56 | 4.10 | 3.52 | 4.79 |
| TINF00291 | 3.94 | 4.79 | | 5.47 | 10.10 | 9.25 | 12.43 |
| TINF00177 | 1.53 | 3.52 | | 5.73 | 4.34 | 6.05 | 4.50 |
| TINF00355 | 51.50 | 53.69 | | 83.95 | 84.74 | 82.91 | 90.86 |
| TINF00502 | 11.93 | 9.25 | | 8.43 | 8.79 | 8.31 | 9.81 |
| TINF00596 | 79.68 | 60.36 | | 87.39 | 87.88 | 91.98 | 96.95 |
| TINF00408 | 185.84 | 74.61 | | 39.97 | 33.44 | 33.13 | 43.44 |
| TINF00464 | 47.97 | 27.38 | | 31.93 | 33.34 | 35.05 | 36.07 |
| TINF00183 | 34.14 | 30.85 | | 54.56 | 31.79 | 38.43 | 34.37 |
| TINF00557 | 72.14 | 67.52 | | 58.37 | 22.85 | 42.81 | 29.46 |
| TINF00159 | 0.11 | 0.61 | | 11.50 | 18.24 | 17.27 | 8.79 |
| TINF00247 | 1.13 | 9.52 | | 382.34 | 300.70 | 480.45 | 219.49 |
| TINF00586 | 380.53 | 765.20 | | 2335.54 | 1381.96 | 3539.12 | 1418.29 |
| TINF00536 | 19.70 | 19.04 | | 59.38 | 30.20 | 61.77 | 30.04 |
| TINF00426 | 87.23 | 64.03 | | 228.10 | 194.66 | 334.15 | 216.76 |
| TINF00174 | 0.84 | 4.03 | | 103.65 | 55.15 | 115.07 | 52.74 |
| TINF00267 | 0.31 | 3.76 | | 148.08 | 134.71 | 180.81 | 69.10 |
| TINF00377 | 0.56 | 6.97 | | 469.51 | 488.62 | 690.86 | 436.68 |
| TINF00470 | 1.49 | 22.08 | | 748.67 | 474.84 | 829.18 | 362.46 |
| TINF00351 | 1.95 | 32.18 | | 971.64 | 461.66 | 1012.96 | 705.01 |
| TINF00195 | 0.00 | 4.25 | | 327.47 | 719.27 | 561.47 | 397.80 |
| TINF00141 | 0.00 | 0.27 | | 10.87 | 35.34 | 27.39 | 9.61 |
| TINF00394 | 9.41 | 48.38 | | 344.96 | 125.33 | 317.25 | 55.44 |
| TINF07874 | 0.00 | 0.88 | | 15.94 | 4.72 | 14.76 | 6.04 |
| TINF00620 | 0.00 | 0.00 | | 0.00 | 0.00 | 0.00 | 0.00 |
| TINF00605 | 0.04 | 0.20 | | 0.77 | 0.39 | 0.41 | 0.49 |
| TINF00458 | 0.12 | 0.14 | | 0.32 | 0.08 | 0.18 | 0.15 |
| TINF00432 | 13.07 | 11.72 | | 14.83 | 15.06 | 11.71 | 11.64 |
| TINF00266 | 53.66 | 88.42 | | 56.91 | 91.81 | 50.93 | 72.36 |
| TINF00548 | 25.10 | 15.54 | | 20.33 | 16.13 | 15.68 | 17.20 |
| TINF00185 | 0.00 | 0.00 | | 0.00 | 0.00 | 0.00 | 0.00 |
| TINF00554 | 0.00 | 0.00 | | 0.13 | 0.18 | 0.14 | 0.40 |
| TINF00588 | 53.66 | 0.00 | | 3.68 | 8.91 | 12.75 | 15.37 |
| TINF00492 | 2.54 | 5.23 | | 8.74 | 4.00 | 8.11 | 7.79 |
