# Supplementary material for: The Genome of Tolypocladium inflatum: Evolution, Organization, and Expression of the Cyclosporin Biosynthetic Gene Cluster
Source: PLoS Genet. 2013 Jun 20;9(6):e1003496. doi: 10.1371/journal.pgen.1003496 (PMC3688495; doi:10.1371/journal.pgen.1003496)
Supplement: Table S6 — Table of q-values, fold change, and RPKM values at six time points (days 2, 4, 6, 8, 10, 12) in analyses of SDB, cuticle, and hemolymph media. Genes in the RNA-Seq defined cluster are shaded green. (DOCX) [file pgen.1003496.s014.docx]

| **Table S6\| Q-values, log_2_ transformed fold change and relative expression levels in comparisons of SDB vs cuticle medium and SDB vs hemolymph medium** | | | |
| --- | --- | --- | --- |
| 1. Q-values for SDB vs Cuticle and SDB vs Hemolymph comparisons. Significant values (q-value<0.05) marked with and *. Some genes were not detected as expressed in one or more conditions. These comparisons are marked as n/a and were tabulated as zero in Figures 6 and 9. Genes in the RNA-Seq defined cluster are shaded green. | | | |
| gene | SDB vs Cuticle q-value | SDB vs Hemolymph q-value |  |
| TINF00496 | 9.75E-01 | 6.14E-01 |  |
| TINF00268 | 9.91E-01 | 2.42E-01 |  |
| TINF00352 | 7.45E-01 | 4.01E-01 |  |
| TINF00373 | 9.08E-01 | 6.29E-02 |  |
| TINF00467 | 9.08E-01 | 1.55E-01 |  |
| TINF00459 | 7.85E-01 | 2.90E-01 |  |
| TINF00234 | 5.62E-01 | 4.46E-01 |  |
| TINF00233 | 7.10E-01 | 6.14E-01 |  |
| TINF00513 | 9.19E-01 | 5.31E-01 |  |
| TINF00291 | 9.50E-01 | 5.91E-01 |  |
| TINF00177 | 5.70E-01 | 3.27E-01 |  |
| TINF00355 | 9.10E-01 | 4.85E-01 |  |
| TINF00502 | 1.00E+00 | 1.17E-01 |  |
| TINF00596 | 5.70E-01 | 4.38E-01 |  |
| TINF00408 | 1.00E+00 | 4.65E-01 |  |
| TINF00464 | 2.23E-01 | 5.07E-01 |  |
| TINF00183 | 8.61E-01 | 4.34E-01 |  |
| TINF00557 | 9.80E-01 | 4.11E-01 |  |
| TINF00159 | 5.70E-01 | 5.96E-01 |  |
| TINF00247 | 1.00E+00 | 5.17E-01 |  |
| TINF00586 | 1.00E+00 | *9.89E-04 |  |
| TINF00536 | 8.14E-01 | 3.95E-01 |  |
| TINF00426 | 9.03E-01 | 5.79E-01 |  |
| TINF00174 | 8.58E-01 | 6.14E-01 |  |
| TINF00267 | *1.72E-02 | *7.04E-04 |  |
| TINF00377 | n/a | *4.36E-02 |  |
| TINF00470 | *9.79E-04 | 2.46E-01 |  |
| TINF00351 | 6.41E-01 | 2.92E-01 |  |
| TINF00195 | n/a | n/a |  |
| TINF00141 | n/a | n/a |  |
| TINF00394 | 3.08E-01 | *1.19E-03 |  |
| TINF07874 | n/a | 4.01E-01 |  |
| TINF00620 | n/a | n/a |  |
| TINF00605 | n/a | 4.16E-01 |  |
| TINF00458 | n/a | n/a |  |
| TINF00432 | 8.91E-01 | 2.97E-01 |  |
| TINF00266 | 5.99E-01 | 8.84E-02 |  |
| TINF00548 | 7.79E-01 | 3.62E-01 |  |
| TINF00185 | n/a | n/a |  |
| TINF00554 | n/a | n/a |  |
| TINF00588 | n/a | n/a |  |
| TINF00492 | 4.13E-01 | 2.86E-01 |  |
|  |  |  |  |
| **gene** | Cuticle log_2_ foldchange | Hemolymph log_2_ foldchange |  |
| TINF00496 | -0.1690 | -0.0271 |  |
| TINF00268 | 0.1622 | 0.6747 |  |
| TINF00352 | 0.4730 | -0.3783 |  |
| TINF00373 | 0.4399 | -1.4281 |  |
| TINF00467 | -0.3228 | -0.8406 |  |
| TINF00459 | 0.5910 | 0.5564 |  |
| TINF00234 | -1.0674 | -0.3993 |  |
| TINF00233 | 0.9535 | -0.0636 |  |
| TINF00513 | 0.6674 | 0.3049 |  |
| TINF00291 | -0.5525 | 0.0375 |  |
| TINF00177 | -1.5502 | 0.6554 |  |
| TINF00355 | -0.4175 | -0.3165 |  |
| TINF00502 | -0.0816 | -1.0571 |  |
| TINF00596 | 0.8494 | -0.3817 |  |
| TINF00408 | -0.0604 | 0.2612 |  |
| TINF00464 | -1.8182 | 0.2113 |  |
| TINF00183 | 0.4776 | 0.3148 |  |
| TINF00557 | -0.1813 | 0.3632 |  |
| TINF00159 | 1.1069 | 0.0156 |  |
| TINF00247 | -0.2895 | -0.7370 |  |
| TINF00586 | -0.1091 | 1.8160 |  |
| TINF00536 | 0.4938 | -0.4286 |  |
| TINF00426 | 0.4142 | -0.1255 |  |
| TINF00174 | 1.0875 | -0.4150 |  |
| TINF00267 | 2.9608 | 2.7748 |  |
| TINF00377 | 0.0000 | 1.9635 |  |
| TINF00470 | 3.2542 | 0.9069 |  |
| TINF00351 | 1.0841 | 0.7279 |  |
| TINF00195 | 0.0000 | 0.0000 |  |
| TINF00141 | 0.0000 | 0.0000 |  |
| TINF00394 | 2.6439 | 3.5850 |  |
| TINF07874 | 0.0000 | 0.9069 |  |
| TINF00620 | 0.0000 | 0.0000 |  |
| TINF00605 | 0.0000 | 1.1699 |  |
| TINF00458 | 0.0000 | 0.0000 |  |
| TINF00432 | -0.4537 | -0.6609 |  |
| TINF00266 | 0.8786 | -1.2355 |  |
| TINF00548 | -0.6992 | -0.5516 |  |
| TINF00185 | 0.0000 | 0.0000 |  |
| TINF00554 | 0.0000 | 0.0000 |  |
| TINF00588 | 0.0000 | 0.0000 |  |
| TINF00492 | 0.0000 | 0.0000 |  |
| 1. Expression levels of genes in *simA* cluster in SDB, cuticle, and hemolymph – Average RPKM across three biological replicates | | | |
|  | SDB_RPKM | Cuticle_RPKM | HEMO_RPKM |
| TINF00496 | 35.75 | 34.71 | 37.56 |
| TINF00268 | 10.69 | 12.34 | 17.69 |
| TINF00352 | 603.39 | 973.90 | 545.70 |
| TINF00373 | 14.57 | 18.64 | 5.43 |
| TINF00467 | 52.79 | 47.21 | 32.35 |
| TINF00459 | 20.41 | 30.36 | 29.68 |
| TINF00234 | 96.80 | 53.90 | 90.01 |
| TINF00233 | 2.34 | 4.57 | 2.07 |
| TINF00513 | 1.93 | 4.97 | 3.42 |
| TINF00291 | 2.39 | 1.49 | 2.47 |
| TINF00177 | 2.58 | 0.61 | 3.58 |
| TINF00355 | 33.74 | 23.68 | 26.41 |
| TINF00502 | 19.23 | 17.09 | 9.44 |
| TINF00596 | 50.39 | 84.94 | 36.21 |
| TINF00408 | 26.51 | 27.36 | 33.02 |
| TINF00464 | 9.72 | 2.71 | 14.46 |
| TINF00183 | 27.23 | 36.35 | 31.85 |
| TINF00557 | 18.46 | 17.55 | 28.14 |
| TINF00159 | 0.14 | 0.36 | 0.18 |
| TINF00247 | 0.79 | 0.57 | 0.40 |
| TINF00586 | 96.46 | 101.91 | 392.69 |
| TINF00536 | 28.57 | 42.71 | 23.76 |
| TINF00426 | 36.50 | 52.86 | 36.52 |
| TINF00174 | 0.40 | 1.19 | 0.33 |
| TINF00267 | 0.13 | 1.18 | 1.19 |
| TINF00377 | 0.65 | 0 | 3.23 |
| TINF00470 | 1.20 | 14.87 | 2.82 |
| TINF00351 | 2.44 | 6.99 | 5.37 |
| TINF00195 | 0 | 0 | 0 |
| TINF00141 | 0 | 0 | 0 |
| TINF00394 | 0.16 | 1.76 | 3.91 |
| TINF07874 | 0.17 | 0 | 0.25 |
| TINF00620 | 0 | 0 | 0 |
| TINF00605 | 0.07 | 0 | 0.14 |
| TINF00458 | 0 | 0 | 0 |
| TINF00432 | 5.28 | 4.14 | 3.70 |
| TINF00266 | 16.50 | 34.18 | 7.45 |
| TINF00548 | 7.75 | 5.85 | 6.05 |
| TINF00185 | 0 | 0 | 0 |
| TINF00554 | 0 | 0 | 0 |
| TINF00588 | 0 | 0 | 0 |
| TINF00492 | 0.70 | 0.17 | 0 |
